# Chronic integrated stress response causes dysregulated cholesterol synthesis in white matter disease

**DOI:** 10.1172/jci.insight.188459

**Published:** 2025-07-15

**Authors:** Karin Lin, Nina Ly, Rejani B. Kunjamma, Ngoc Vu, Bryan King, Holly M. Robb, Eric G. Mohler, Janani Sridar, Qi Hao, José Zavala-Solorio, Chunlian Zhang, Varahram Shahryari, Nick van Bruggen, Caitlin F. Connelly, Bryson D. Bennett, James J. Lee, Carmela Sidrauski

**Affiliations:** 1 Calico Life Sciences LLC, South San Francisco, California, USA.; 2 AbbVie, North Chicago, Illinois, USA.

**Keywords:** Cell biology, Metabolism, Neuroscience, Cholesterol, Demyelinating disorders, Mouse models

## Abstract

Maladaptive integrated stress response (ISR) activation is observed in human diseases of the brain. Genetic mutations of eIF2B, a critical mediator of protein synthesis, cause chronic pathway activation resulting in a leukodystrophy, but the precise mechanism is unknown. We generated N208Y eIF2B-α mice and found that this metabolite binding mutation led to destabilization of eIF2B-α, a systemic ISR, and neonatal lethality. 2BAct, an eIF2B activator, rescued lethality and significantly extended the lifespan of this severe model, underscoring its therapeutic potential in pediatric disease. Continuous treatment was required for survival, as withdrawal led to ISR induction in all tissues and rapid deterioration, thereby providing a model to assess the impact of the ISR in vivo by tuning drug availability. Single nuclei RNA-seq of the CNS identified astrocytes, oligodendrocytes, and ependymal cells as the cell types most susceptible to eIF2B dysfunction and revealed dysfunctional maturation of oligodendrocytes. Moreover, ISR activation decreased cholesterol biosynthesis, a process critical for myelin formation and maintenance. As such, persistent ISR engagement may contribute to pathology in other demyelinating diseases.

## Abstract 2

Neonatal lethal Vanishing White Matter model is rescued by eIF2B activator and reveals dysregulated cholesterol synthesis as a consequence of chronic integrated stress response induction.

## Graphical abstract

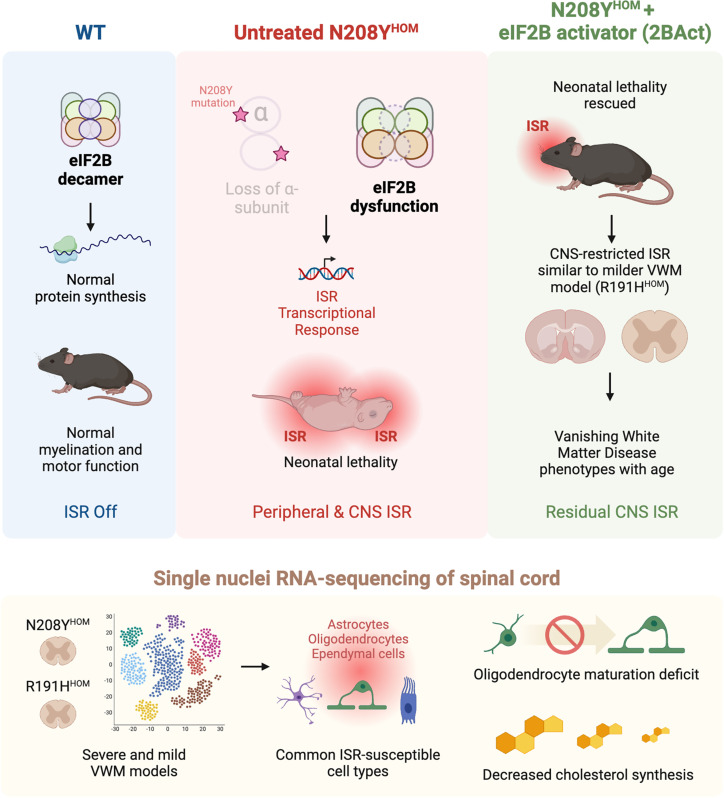

## Introduction

The integrated stress response (ISR) is a conserved signaling pathway triggered by a variety of insults that include endoplasmic reticulum (ER) stress, mitochondrial stress, amino acid deprivation, and viral infection (1). Four kinases with distinct sensor domains are activated by these diverse inputs and converge on the phosphorylation of eukaryotic initiation factor 2 (eIF2), a GTPase responsible for initiating mRNA translation. This phosphorylation converts eIF2 from a substrate to an inhibitor of its guanine nucleotide exchange factor (GEF), eIF2B, reducing its enzymatic activity and leading to a decrease in bulk protein synthesis (2, 3). Concomitantly, translation of a subset of stress-associated mRNAs is increased, including the transcription factors ATF4 and ATF5, which upregulate expression of many ISR target genes, including secreted molecules with broad effects such as FGF21 and GDF15 (4, 5). ISR activation broadly remodels the metabolome and lipidome in cells playing an important role in cell fate, differentiation, and tissue and organismal homeostasis (6, 7). Thus, understanding the role of ISR activation in various disease contexts remains an important goal in the field.

The essential stress sensor and key ISR effector eIF2B is a highly complex and regulated translation initiation factor. It is a decamer composed of a central regulatory core (α_2_β_2_δ_2_), with homology to a sugar metabolizing enzyme, flanked by 2 catalytic subcomplexes (γε) (8, 9). Mutations in any of its 5 subunits can reduce complex stability and GEF activity in vitro (10). In humans, these mutations are associated with vanishing white matter (VWM) disease, a rare recessive leukodystrophy characterized by the progressive disappearance of white matter (11). Early onset childhood disease, which features rapid progression, severe motor disabilities, and early death, is the most common form, whereas adolescent and adult-onset disease is milder, slower progressing, and features cognitive decline (11). More than 60% of patients are compound heterozygotes for mutations in the same subunit of eIF2B and disease progression likely inversely scales with the level of residual eIF2B activity (12). Pathogenic VWM mutations (R484W eIF2B-δ, R191H eIF2B-ε, and R132H eIF2B-ε) in mice likewise manifest as neurological symptoms, and chronic ISR activation is restricted to the central nervous system (CNS) in these mutants (13–15).

There is currently no treatment that targets the underlying cause of VWM disease. We previously showed that preventive treatment of homozygous R191H eIF2B-ε mice (hereafter referred to as R191H^HOM^) with an orally bioavailable small molecule eIF2B activator, 2BAct, prevents the development of VWM pathophysiology, fully suppresses the ISR, and normalizes the brain transcriptome and proteome (13), revealing its potential in modifying VWM etiology. eIF2B activators bind to the interface of the core subcomplex, stabilizing the decamer, which is the most active form of the enzyme (16–19). Full complex assembly is normally regulated by the eIF2B-α regulatory subunit (20), which forms a homodimer (α_2_) that glues the 2 halves of the complex together promoting decamer formation (10, 21). We discovered that sugar phosphates, a natural class of metabolites, bind to eIF2Bα and further stabilize the decamer, thus boosting complex activity (22). A single amino acid substitution (N208Y) in the sugar phosphate binding pocket of the α subunit abolished metabolite binding and GEF activity enhancement (22). Notably, in humans, the N208Y mutation (622A→T) in the *EIF2B1* locus has been reported as a compound heterozygous in a single VWM patient (23) but the cellular and physiological consequences of modulating sugar phosphate binding of eIF2B have not been determined.

Here, we report the cellular and organismal effects of this metabolite binding VWM-associated mutation. We demonstrate that the N208Y substitution on eIF2B-α results in a drastic decrease in the level of eIF2B-α protein across tissues in homozygous N208Y knock-in mice (hereafter referred to as N208Y^HOM^), leading to ISR induction in all tissues and neonatal lethality. Remarkably, treatment with 2BAct allowed a lifespan of approximately 11 months. Continuous drug availability was required to sustain life as its withdrawal led to ISR induction across tissues, fast accumulation of the circulating factors GDF15 and FGF21, and rapid physiological deterioration. We found that a subset of CNS glial cells had a chronic ISR and these same cell types were also susceptible to the milder R191H eIF2B-ε mutation. Single cell transcriptomic analysis further revealed a defect in oligodendrocyte maturation and identified cholesterol synthesis as a common pathway downregulated in stressed cells. The ability to pharmacologically modulate residual eIF2B function in the severe N208Y^HOM^ model allows controlled activation of the ISR across peripheral organs as well, allowing us to show that rewiring of cholesterol homeostasis is a metabolic output of the ISR shared across multiple tissues.

## Results

### Mutations that reduce sugar phosphate binding decreased levels of eIF2B-α in cells and induced the ISR.

**Figure 1:**
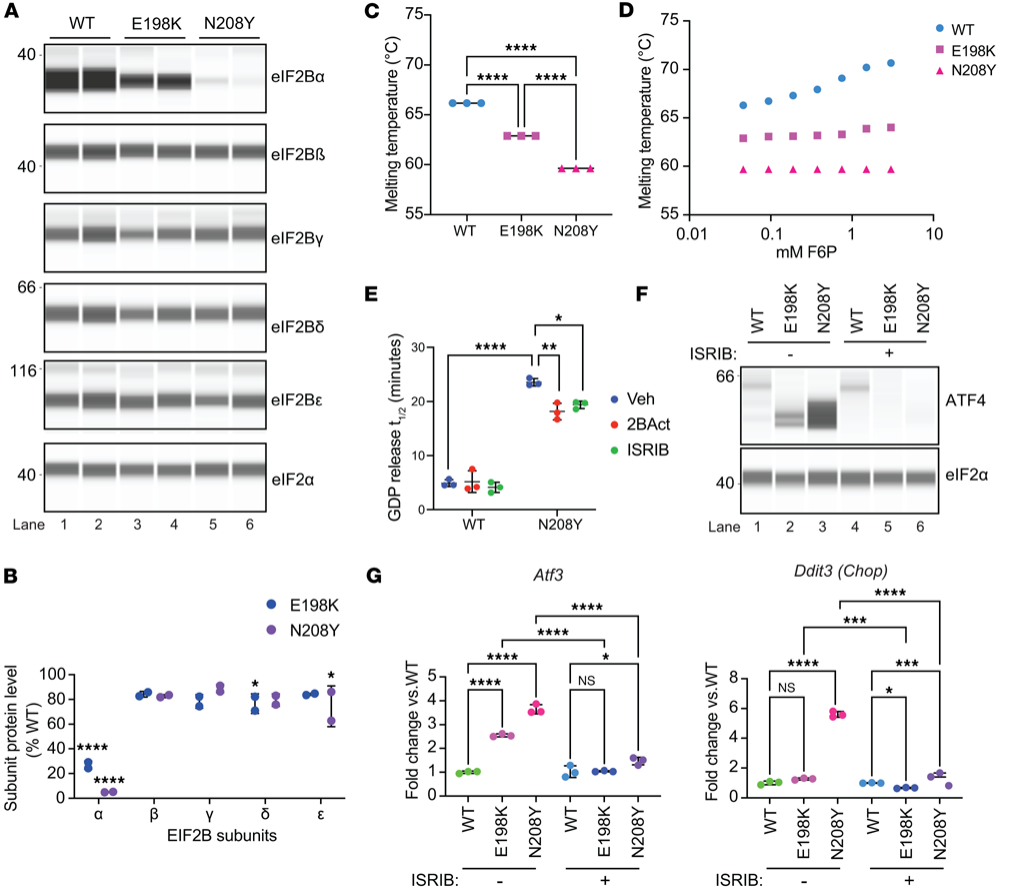
Mutations that impair sugar phosphate binding lead to a decrease in the level of eIF2B-α, reduced GEF activity, and induction of the ISR. (**A**) Immunoblot analysis of eIF2B subunits of MIN-6 WT, E198K or N208Y cells maintained in 200 nM ISRIB. Data shown are representative of 2 clones per genotype. (**B**) Quantification of the eIF2B subunit level shown in **A**. eIF2B subunit expression was normalized to eIF2-α and expressed as % of WT. Note reduced level of eIF2B-α in the mutants as compared with WT cells. Significance is shown for comparisons of each mutant to WT. *n* = 2. Error bars are SD. Two-way ANOVA, Holm-Sidak post-hoc. (**C** and **D**) Calculated midpoint thermal unfolding of recombinant human WT, E198K, or N208Y eIF2B-α using NanoDSF without sugar phosphate (**C**) and with F6P (**D**). *n* = 3. Error bars (too small to be visible in the graph) are SD. SD: WT (0.0001), E198K (0.0002), N208Y (0.0006). One-way ANOVA, Tukey post-hoc. (**E**) In vitro GEF activity is measured as the half-life of fluorescently labeled GDP release from purified human eIF2 when added to WT or N208Y MIN-6 cell lysates in the absence or presence of 500 nM 2BAct or ISRIB. *n* = 3. Error bars are SD. Two-way ANOVA, Tukey post-hoc. (**F**) Immunoblot of ATF4 protein and eIF2-α with or without ISRIB withdrawal for 24 hours. (**G**) Transcript levels of ISR target genes, *Atf3* and *Ddit3*, with or without ISRIB withdrawal for 24 hours. *n* = 3 of one representative clone. Error bars are SD. One-way ANOVA, Tukey post-hoc. *P < 0.05, **P < 0.01,***P < 0.001, ****P < 0.0001.

To determine the impact of sugar phosphate binding mutations on the stability and activity of eIF2B in cells, we introduced the VWM-associated N208Y mutation or a E198K mutation (which partially impairs fructose-6-phosphate [F6P] binding) (22) into mouse MIN-6 β cells (Supplemental Figure 1A; supplemental material available online with this article; https://doi.org/10.1172/jci.insight.188459DS1). We observed a striking reduction in the level of eIF2B-α in both binding mutants, whereas the levels of other complex subunits were only subtly affected (Figure 1, A and B), with the magnitude of eIF2Bα reduction being proportional to the decrease in F6P binding. N208Y abolished F6P binding and resulted in a 95% decrease in the level of eIF2B-α in cells, whereas E198K retained the ability to bind sugar phosphates, although with a 10-fold decrease in affinity (22) and resulted in a 74% reduction in subunit abundance (Figure 1B). Western blot of purified N208Y and E198K eIF2B-α dimers confirmed that sugar phosphate binding mutations did not disrupt the antibody epitope interaction (Supplemental Figure 1B).

To evaluate whether reduction of eIF2B-α in cells was due to an inherent instability of these sugar phosphate binding mutant proteins, we performed nano differential scanning fluorimetry (NanoDSF) of purified recombinant WT, N208Y, and E198K eIF2B-α and determined their midpoint of thermal unfolding (Tm). In the absence of F6P, both mutant proteins showed a significant reduction in thermal stability compared with WT eIF2B-α (WT: Tm = 66.2°C, E198K: Tm = 62.9°C, and N208Y: Tm = 59.6°C, Figure 1C). As previously reported (22), addition of F6P further increased the stability of WT eIF2B-α. However, F6P did not enhance the intrinsic stability of purified E198K or N208Y eIF2B-α (Figure 1D). Glucose-1-phosphate (a negative control, as only 5’ and 6’ sugar phosphates can bind and activate eIF2B), as expected, did not increase the stability of any of the proteins (Supplemental Figure 1C). Thus, the E198K and N208Y mutations have a significant effect on the intrinsic stability of eIF2B-α and prevent further stabilization by metabolite binding in vitro, implicating metabolite binding in maintaining the stability of eIF2B-α in cells.

Given eIF2B’s essential role in catalyzing GTP exchange of eIF2, we next measured the GEF activity of endogenous eIF2B complexes from lysates of either N208Y or WT MIN6 cells cultured without the eIF2B activator, ISRIB (18). As shown in Figure 1E, in the absence of an eIF2B activator, mutant lysates showed a dramatic reduction in GEF activity (slower GTP exchange) compared with WT (23.6 minutes **±** 0.681 vs 4.9 minutes **±** 0.649). Functionally, this resulted in increased ATF4 protein and induction of the ISR transcriptional targets *Atf3* and *Chop* (Figure 1, F and G) in N208Y cells. Consistent with a less severe reduction of eIF2B-α, the increase in ATF4 protein and ISR target genes was smaller in E198K cells (Figure 1, F and G). Both ISRIB and 2BAct at saturating concentrations (500 nM) boosted the GEF activity of N208Y lysates, though not back to WT levels (Figure 1E). Notably, this small rescue of GEF activity by ISRIB was sufficient to attenuate ATF4 protein levels as well as *Atf3* and *Chop* transcript induction (Figure 1, F and G). Not surprisingly, ISRIB also attenuated the ISR in the milder E198K mutant (Figure 1, F and G). The large fold change in ISR activation markers in N208Y cells indicated that this is a particularly severe VWM-associated pathogenic allele.

### Homozygous N208Y eIF2B-α mutation is neonatal lethal in mice.

**Figure 2:**
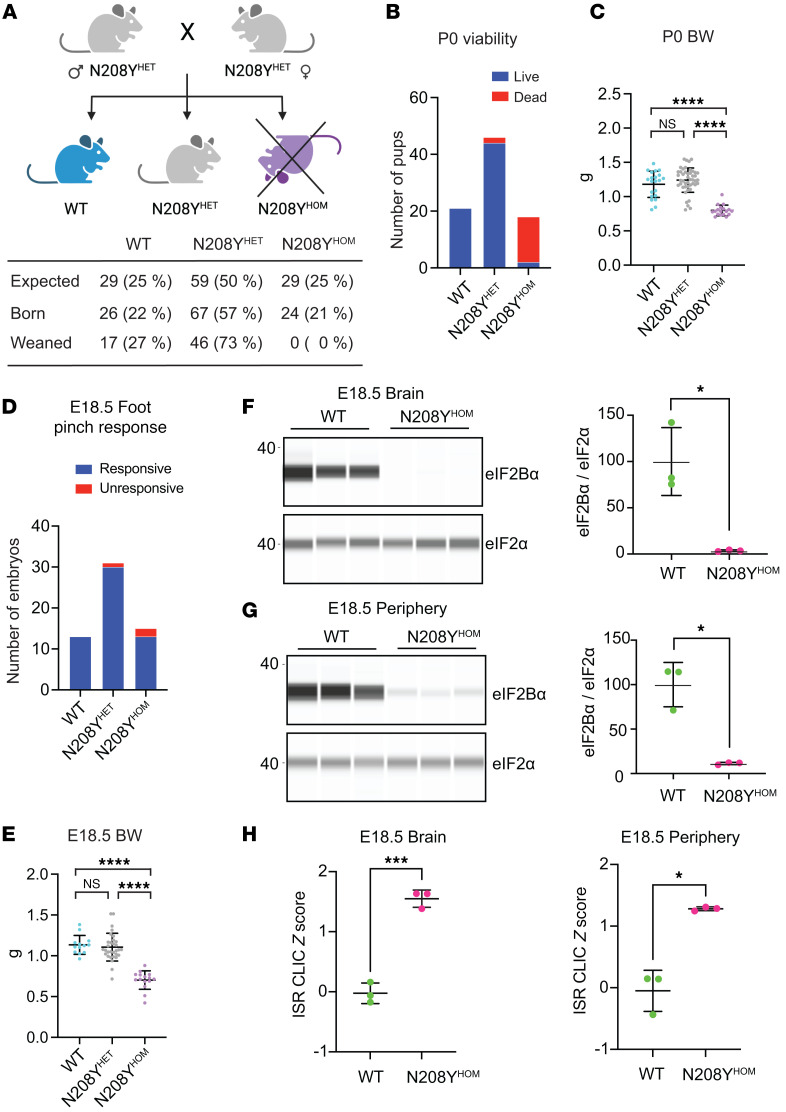
N208Y^HOM^ mice exhibit neonatal lethality and reduced body weight. (**A**) Breeding strategy and number of pups born and weaned for each genotype. (**B** and **C**) Number of P0 pups found dead or alive (**B**) and their body weight measurements (**C**) for each genotype. WT, *n* = 21; N208Y^HET^, *n* = 46, N208Y^HOM^, *n* = 18 from 13 litters. (**D** and **E**) Foot pinch responsiveness (**D**) and body weight measurements (**E**) of E18.5 embryos for each genotype. WT, *n* = 13; N208Y^HET^, *n* = 31, N208Y^HOM^, *n* = 15 from 7 litters. (**C** and **E**) Kruskal-Wallis test with Dunn’s multiple comparisons test. (**F** and **G**) Immunoblot of eIF2B-α subunit in E18.5 brain (**F**) and pooled peripheral organ (**G**) lysates from WT and N208Y^HOM^. Graphs show quantification of eIF2B-α bands, normalized to eIF2-α expression and represented as % of WT expression. *n* = 3 biological replicates, Error bars are SD. Welch’s *t* test. (**H**) Average z-score of ISR CLIC genes calculated from nCounter gene expression profiling of brain and pooled peripheral organs from E18.5 embryos. *n* = 3 biological replicates, Error bars are SD. Welch’s *t*-test *P < 0.05, ***P < 0.001, ****P < 0.0001.

To investigate the impact of impaired sugar phosphate binding in vivo, we generated a N208Y mutant mouse strain via targeted mutagenesis (Supplemental Figure 2A). Heterozygous *Eif2b1^N208Y/+^* (hereafter referred to as N208Y^HET^) mice were viable and grew and bred normally. Breeding of N208Y^HET^ mice yielded pups of all three genotypes at the expected mendelian ratio at birth, but no viable N208Y^HOM^ pups remained at weaning (Figure 2A). Daily monitoring revealed that the majority of WT and N208Y^HET^ mice were alive at postnatal day 0 (P0), whereas only 2 out of 18 N208Y^HOM^ newborns were found alive after birth, and all died within 24 hours (Figure 2B and Supplemental Figure 2B). Body weight measurements revealed that N208Y^HOM^ pups were significantly smaller than N208Y^HET^ or WT counterparts (Figure 2C).

To further characterize the impact of the mutated eIF2B-α subunit, we isolated embryonic day 18.5 (E18.5) embryos, the terminal embryonic stage right before birth. We assessed the viability of the embryo via foot pinch test and found that 86.7 % of N208Y^HOM^ embryos (13 out of 15) responded (Figure 2D and Supplemental Figure 2C), suggesting that most N208Y^HOM^ mice are born alive but perish shortly after birth. Despite body weight measurements of E18.5 N208Y^HOM^ embryos being significantly lower than their N208Y^HET^ and WT littermates (Figure 2E), histopathological features were surprisingly indistinguishable between genotypes at both E18.5 and P0 (Supplemental Figure 2D). Moreover, similar to MIN-6 cells, eIF2B-α levels were drastically reduced in both the brain and peripheral tissues of N208Y^HOM^ embryos (Figure 2, F and G). A concomitant significant increase in the transcripts of ISR pathway genes (a panel of 95 ISR target genes identified by CLIC (Clustering by inferred coexpression) analysis described in Wong 2019 (13), with the addition of *Fgf21*) was detected in both the CNS and periphery (Figure 2H and Supplemental Table 1), indicating widespread ISR activation in utero. Chronic ISR induction in N208Y^HOM^ embryos likely leads to reduced body weight, but the lack of embryonic lethality and absence of gross morphological defects, despite near absence of the essential eIF2B-α protein and significant ISR activation, was unanticipated.

We have thus generated a mouse model that enables in vivo assessment of the impact of a severe loss-of- function mutations in eIF2B. These results indicate that the N208Y mutation in eIF2B-α destabilizes the subunit in both central and peripheral tissues, leading to early, in utero, systemic ISR pathway activation and in neonatal lethality.

### eIF2B activator drug 2BAct rescues neonatal lethality of N208Y^HOM^ mice.

**Figure 3:**
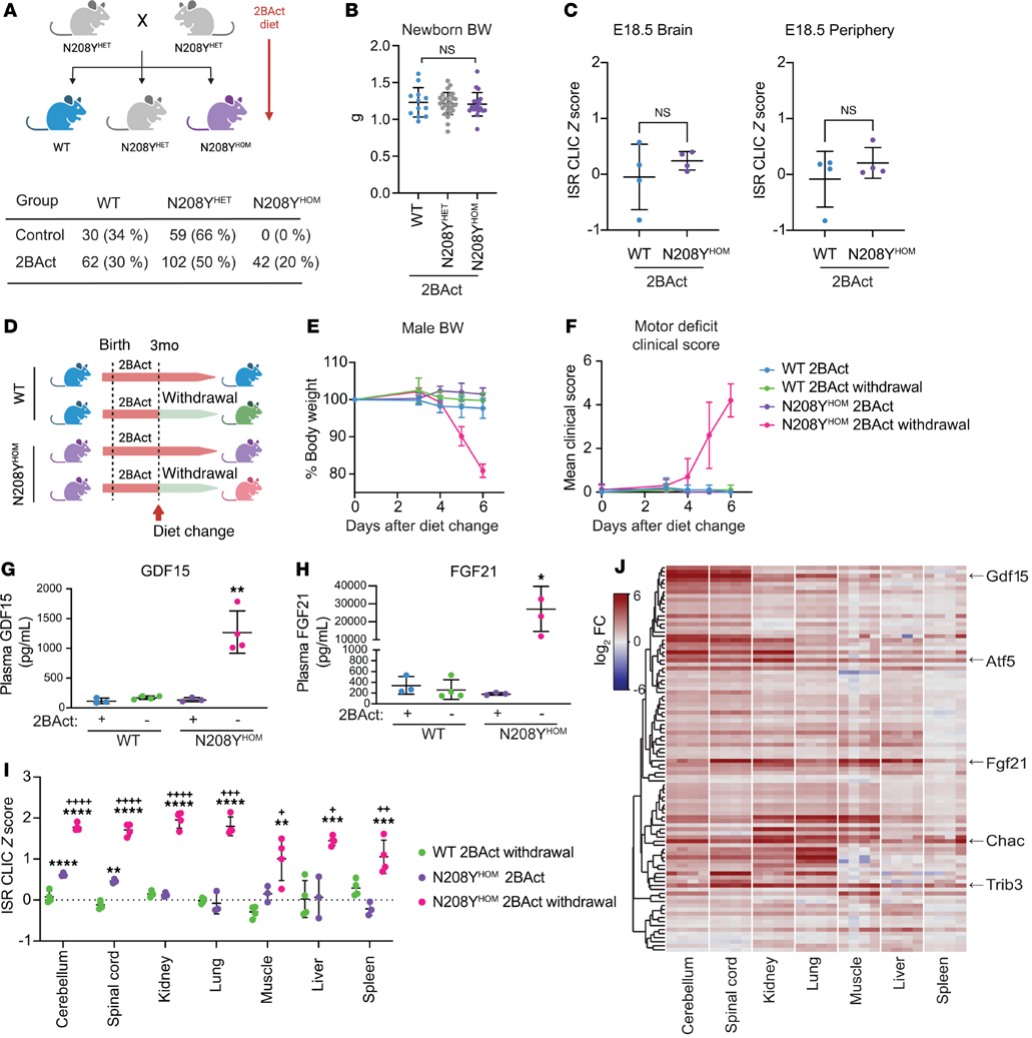
2BAct rescues early lethality of N208Y^HOM^ mice characterized by a systemic integrated stress response. (**A**) Breeding strategy with 2BAct medicated diet and postweaning pup counts by genotype. (**B**) Body weights of newborn pups treated with 2BAct. WT (*n* = 12), N208Y^HET^ (*n* = 27), and N208Y^HOM^ (*n* = 20). Kruskal-Wallis, Dunn’s post hoc test. (**C**) Average ISR CLIC gene z-scores from brain and pooled peripheral organs at E18.5 with 2BAct. *n* = 3–4, Error bars are SD. Welch’s *t* test. (**D**) Schematic of 2BAct withdrawal strategy of 2BAct-rescued WT and N208Y^HOM^ mice. (**E** and **F**) Body weight change (**E**) and mean motor deficit clinical score (**F**) of 4-month-old male mice after 2BAct withdrawal. WT +2BAct, *n* = 5; WT withdrawn, *n* = 5; N208Y^HOM^ +2BAct, *n* = 4; N208Y^HOM^ withdrawn, *n* = 5. (**G** and **H**) ELISA of plasma GDF15 (**G**) and FGF21 (**H**) 3 days after 2BAct withdrawal from 4-month-old male WT and N208Y^HOM^ mice. Error bars are SD. Welch’s *t* test. (**I**) Average z-score of ISR CLIC genes in multiple tissues from 4-month-old male N208Y^HOM^ +2BAct mice or WT and N208Y^HOM^ mice following 2BAct withdrawal, normalized to WT +2BAct littermates. *n* = 3–4, error bars are SD. One-way ANOVA, Dunnett’s post hoc vs WT +2BAct for comparison between WT +2BAct and N208Y^HOM^ +2BAct or N208Y^HOM^ withdrawn. Student’s *t* test for comparison between N208Y^HOM^ +2BAct and N208Y^HOM^ withdrawn, ^+^*P* < 0.05, ^++^*P* < 0.01, ^+++^*P* < 0.001, ^++++^*P* < 0.0001. (**J**) Heatmap of ISR CLIC genes across tissues from 4-month-old male N208Y^HOM^ withdrawn mice normalized to WT +2BAct littermates. (**I** and **J**) WT +2BAct, *n* = 3; WT withdrawn, *n* = 4; N208Y^HOM^ +2BAct, *n* = 3; N208Y^HOM^ withdrawn, *n* = 4. *P < 0.05, **P < 0.01, ***P < 0.001, ****P < 0.0001.

2BAct was sufficient to fully attenuate the CNS-restricted ISR, restore body weight gain, prevent motor deficits and loss of myelin with age in R191H^HOM^ mice (13), a milder disease model harboring a mutation in the catalytic subunit eIF2B-ε. We therefore sought to test whether 2BAct could rescue early lethality in a model where ISR activation was expanded to peripheral tissues. To this end, we provided either 2BAct-supplemented (30 mg/kg) or control diet to N208Y^HET^ breeders and maintained their respective diets during the gestational and nursing periods, as well as to their offspring after weaning. As expected, the control diet group did not yield N208Y^HOM^ pups after weaning (χ^2^ test *P* < 1 × 10^–6^). In contrast, we observed a remarkable rescue in the group fed with 2BAct. N208Y^HOM^ mice represented 20% of viable pups at weaning, which is not significantly different from the expected Mendelian ratio (χ^2^ test *P* = 0.142) (Figure 3A) and differences in P0 body weight were no longer observed across genotypes (Figure 3B). Notably, 2BAct was able to fully attenuate the ISR in E18.5 N208Y^HOM^ embryos (Figure 3C and Supplemental Figure 3A) even though eIF2B-α protein levels were not rescued in the brain and peripheral tissues of treated mice (Supplemental Figure 3B). The ability of 2BAct to rescue neonatal lethality caused by near absence of the α-subunit suggests that its mechanism of action as a molecular stapler of the eIF2B complex can sufficiently boost the activity of the octameric eIF2B complex (β_2_δ_2_γ_2_ε_2_) in vivo. This efficacy underscores the applicability of eIF2B activator drugs for VWM mutations that cause early onset disease and lethality in humans.

### 2BAct withdrawal led to systemic ISR activation and fast physiological decline in adult N208Y^HOM^ mice.

In untreated R191H^HOM^ mice, ISR activation was detected only in the CNS, despite the mutation being present in all cells (13). We sought to determine in which tissues the ISR was upregulated, and the impact of 2BAct, in adult animals harboring the more severe N208Y mutation. To this end, we switched mice from the 2BAct-containing to control diet at 3 to 4 months of age and assessed the physiological consequences by daily monitoring of body weight and motor performance using the clinical scoring system described in Traka (24) (Figure 3D). Starting at Day 4, 2BAct-withdrawn N208Y^HOM^ mice showed rapid decline, losing more than 15% of their body weight within 2 days (Figure 3E and Supplemental Figure 3C). This coincided with the onset of ataxia and tremor, which quickly worsened (Figure 3F), establishing that 2BAct is required to maintain a physiological state that is compatible with life. Given the rapid onset of motor deficits upon drug withdrawal, we first investigated whether demyelination could contribute to the decline. Histological and IHC analyses of spinal cords from 3-month-old female 2BAct-maintained N208Y^HOM^ mice showed an increase in OLIG2-positive oligodendrocyte lineage cell number, but no differences in myelination (Luxol Fast Blue staining) were detected at this young age (Supplemental Figure 3, D and E). After 7 days of drug withdrawal, there was no detectable change in GFAP, OLIG2, or myelin staining, suggesting that the onset of motor deficits was not due to rapid demyelination (Supplemental Figure 3, D and E).

We next measured the level of plasma GDF15 and FGF21, 2 secreted factors and known targets of the ISR that have wide metabolic and systemic effects (25–27). No difference in the level of circulating GDF15 and FGF21 was observed in 2BAct-maintained N208Y^HOM^ mice, but withdrawal of drug from the diet led to significant accumulation of both, a 9.3- and 146-fold increase in GDF15 and FGF21, respectively (Figure 3, G and H), indicating systemic activation of the pathway. To evaluate the status of the ISR across organs, we collected CNS (cerebellum and spinal cord) and several peripheral tissues (kidney, lung, muscle, liver, and spleen) from WT and N208Y^HOM^ mice maintained on 2BAct or 3 days after switching to a control diet and assessed gene expression. No significant change in ISR pathway activation, as measured by the CLIC z-score, was observed in any tissue in 2BAct-withdrawn WT mice when compared with their maintained counterparts (Supplemental Figure 3F). In contrast, ISR gene expression was significantly increased in all tissues surveyed when drug was withdrawn from N208Y^HOM^ animals (Figure 3I), highlighting the ability of 2BAct to continuously boost eIF2B activity and suppress the pathway systemically in adult mutant mice.

This N208Y^HOM^ model offers an unprecedented opportunity to interrogate the effects of acute ISR activation, in both CNS and peripheral tissues in vivo, by tuning the availability of 2BAct. To this end, we observed heterogeneity in the pattern of induction of the 96 individual ISR CLIC genes across the tissues surveyed (Figure 3J). *Atf5*, *Chac1*, and *Trib3* were significantly elevated in all tissues and may constitute a core ISR module (Figure 3J and Supplemental Table 2). *Gdf15* was highly induced in both CNS tissues but to a lesser extent in peripheral tissues. *Fgf21* was highly upregulated in the spinal cord (> 98-fold) but modestly in the cerebellum (> 7-fold) and was also significantly induced in all peripheral tissues upon removal of 2BAct. The unique pattern of ISR target gene induction likely reflects heterogeneity in the expression of transcriptional coactivators and corepressors that modulate the effect of ISR-dependent transcription factors such as ATF4 and ATF5 across tissues.

### 2BAct-treated N208Y^HOM^ mice have a chronic CNS-restricted ISR and lose myelin with age.

Although 2BAct rescued early lethality and attenuated the ISR across tissues, it did not fully suppress the response in the cerebellum and spinal cord of N208Y^HOM^ mice (Figure 3I). CNS-restricted ISR activation is a characteristic of R191H^HOM^ untreated mice, starting at 4 weeks of age (13, 28). To evaluate potential causes of partial ISR attenuation by 2BAct in the CNS, we first assessed compound exposure and found no significant difference in free unbound 2BAct between WT and N208Y^HOM^ mice in tissues nor in plasma (Supplemental Figure 4A). Although exposure of 2BAct is lower in CNS versus peripheral tissues, drug concentration in the brain was similar to that measured in R191H^HOM^ mice (approximately 15-fold above the reported EC_50_) (13) which fully suppressed the ISR in this milder VWM model. We also confirmed that the EC_50_ of 2BAct in N208Y cells (27.8 nM) (Supplemental Figure 4, B and C) is similar to that reported in WT cells (33 nM) (13). These results indicated that incomplete ISR suppression is not due to lack of saturation of the target eIF2B by drug in the CNS.

We then measured the level of eIF2B subunits and, as in the N208Y^HOM^ embryos, observed a drastic reduction in the levels of eIF2B-α protein in adult mutant mice in all tissues (> 90% reduction in brain, liver, and lung, as well as a 70% reduction in kidney, Supplemental Figure 4, D and E). The comparable reduction of eIF2B-α in the brain, liver, and lung indicated that differential eIF2B-α levels are unlikely to explain partial drug response in the CNS. However, cell type–specific variation in eIF2B-α abundance could contribute to ISR activation. These results indicated that incomplete ISR suppression in the CNS of mutant mice is not due to lack of saturation of the target eIF2B by drug but rather an intrinsic disposition of this tissue to eIF2B hypomorphic activity.

**Figure 4:**
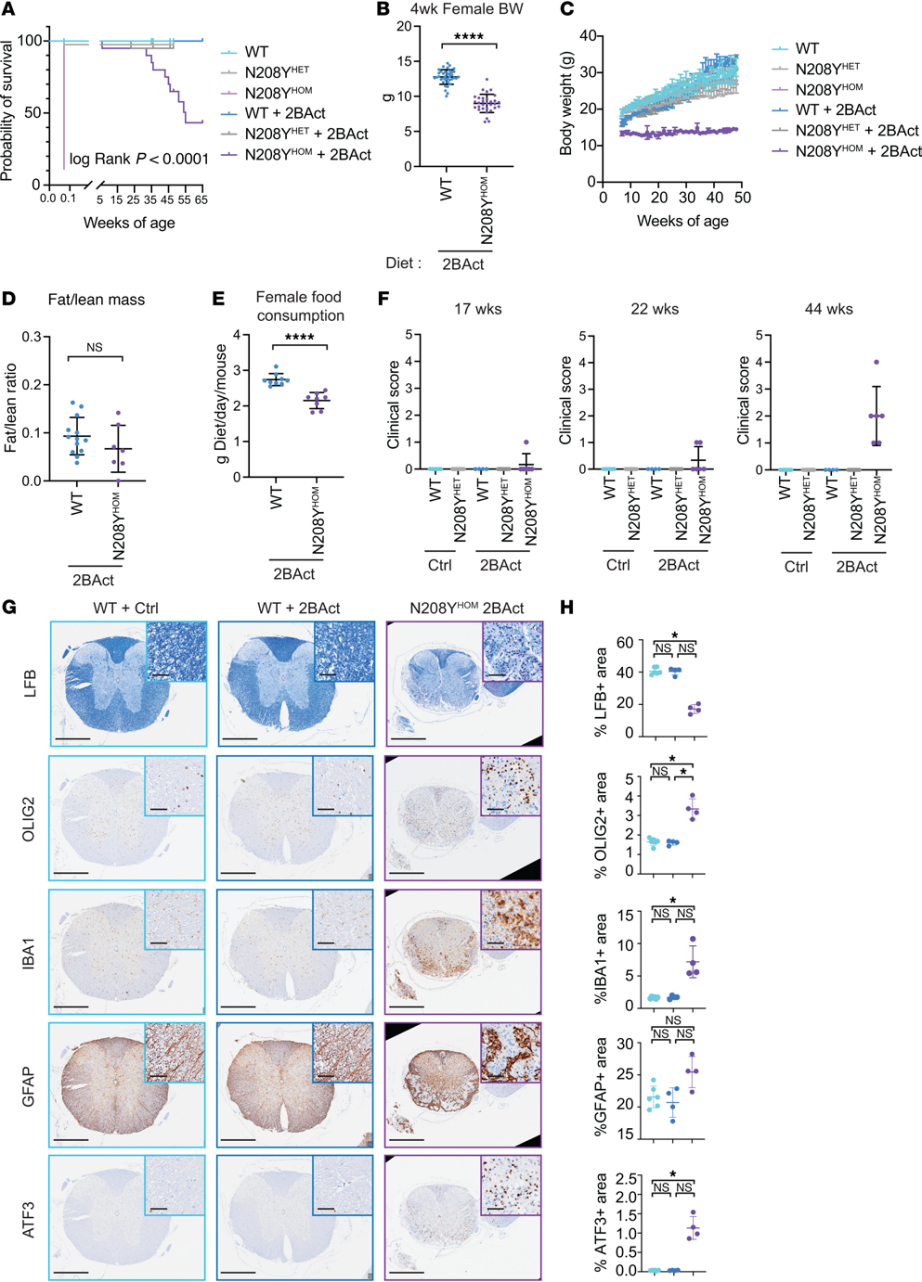
2BAct-treated N208Y^HOM^ mice display VWM phenotypes with age. (**A**) Kaplan-Meier survival: WT, *n* = 37; N208Y^HET^, *n* = 80; N208Y^HOM^, *n* = 18; WT +2BAct, *n* = 19; N208Y^HET^ +2BAct, *n* = 20, N208Y^HOM^ +2BAct, *n* = 20. *P* < 0.0001, Log-rank (Mantel-Cox) test. (**B**) Body weight of 4-week-old female WT (*n* = 38) and N208Y^HOM^ (*n* = 31) mice with 2BAct. Student’s *t* test. (**C**) Body weight of WT, N208Y^HET^, and N208Y^HOM^ mice ± 2BAct. Female WT, *n* = 3; N208Y^HET^, *n* = 13; WT +2BAct, *n* = 2; N208Y^HET^ +2BAct, *n* = 5; N208Y^HOM^ +2BAct, *n* = 2. *P* = 1.1 **×** 10^–9^ for N208Y^HOM^ +2BAct vs N208Y^HET^ +2BAct, *P* = 7.75 **×** 10^–8^ for N208Y^HOM^ +2BAct vs WT +2BAct, not significant for N208Y^HET^ +2BAct vs WT +2BAct, Linear mixed model (both sexes) with a post hoc test. (**D**) Fat/Lean mass ratio of 3-month-old female WT (*n* = 13) and N208Y^HOM^ (*n* = 7) mice (EchoMRI). Student’s *t* test. (**E**) Female food consumption at 5–6 weeks of age. WT +2BAct, *n* = 9; N208Y^HOM^ +2BAct, *n* = 8. Error bars are SD. Student’s *t* test. (**F**) Motor deficit clinical scores of WT, N208Y^HET^, and N208Y^HOM^ male and female mice ± 2BAct at 17, 22, and 44 weeks of age. WT, *n* = 9; N208Y^HET^, *n* = 27, WT +2BAct, *n* = 4, N208Y^HET^ +2BAct, *n* = 14; N208Y^HOM^ +2BAct, *n* = 6. (**G** and **H**) Representative Luxol Fast Blue staining and OLIG2, IBA1, GFAP, and ATF3 IHC images of the thoracic spinal cord (**G**) and quantification (**H**). WT +Ctrl, *n* = 6; WT +2BAct, *n* = 4; N208Y^HOM^ +2BAct, *n* = 4. Scale bars: 500 μm (50 μm for insets). Error bars are SD. Kruskal-Wallis test, Dunn’s post-hoc. *P < 0.05, **P < 0.01, ***P < 0.001, ****P < 0.0001.

Given the persistent CNS ISR in 2BAct-maintained N208Y^HOM^ mice, we longitudinally monitored animals to evaluate the development of VWM-associated disease phenotypes. We observed that 95% of 2BAct-fed N208Y^HOM^ mice were alive beyond 32 weeks of age and 43% survived to 65 weeks of age (Figure 4A), demonstrating the continued ability of 2BAct to boost the function of this severe allele, significantly extending lifespan. Despite the dramatic effect observed over untreated N208Y^HOM^, mice which die right after birth, 2BAct-maintained mice did not live as long as WT animals. Further characterization revealed that they had significantly lower body weight as early as 4 weeks (Figure 4B and Supplemental Figure 4F) and failed to gain additional weight with age (Figure 4C and Supplemental Figure 4G). In contrast, untreated and 2BAct-treated N208Y^HET^ and WT littermates gained weight during this time period (7–50 weeks). Echo-MRI showed a significant reduction in both fat and lean mass in 3-month-old 2BAct-treated N208Y^HOM^ mice (Supplemental Figure 4H), but the fat-to-lean ratio was not different between groups (Figure 4D). Measurement of daily food consumption indicated that drug-treated mutant mice eat significantly less than their WT counterparts (Figure 4E and Supplemental Figure 4I). Therefore, 2BAct-treated N208Y^HOM^ mice are born a normal size (Figure 3B) but fail to gain weight along the normal trajectory and this is, at least partly, due to reduced food consumption. We found that expression of *Gdf15* remained significantly elevated (40-fold increase) in both the cerebellum and spinal cord of 2BAct-maintained N208Y^HOM^ mice (Supplemental Figure 4J). GDF15 is known to impact body weight by reducing food intake (27), and its increased expression could be the principal contributor to the reduction in food intake and body weight. Notably, GDF15 levels in cerebrospinal fluid (CSF) correlated with ISR induction in R191H^HOM^ mice and are also elevated in the CSF of VWM patients (25).

We again utilized the clinical scoring system (24) to track motor performance and observed that by 44 weeks all mutant mice exhibited ataxia with clinical scores ranging from 1 to 4 (Figure 4F). IHC analysis of spinal cords and brains from 11-month-old 2BAct-treated N208Y^HOM^ mice (only 53.3 % survived to this age, Figure 4A) showed a significant reduction of the white matter, as well as increased inflammation and gliosis in both CNS tissues (Figure 4, G and H and Supplemental Figure 5, A–D).

In summary, although 2BAct significantly extended the lifespan of N208Y^HOM^ mice, it did not fully suppress the ISR in the CNS nor prevent development of pathology with age, indicating incomplete rescue of this severe allele. Notably, the phenotype of 2BAct-treated N208Y^HOM^ mice closely resembled that of untreated R191H^HOM^ mice, a later onset mouse model of VWM (13). The shared findings across models, along with the clinical observation that eIF2B partial loss-of-function mutations primarily manifest as a neurological disease in humans, strongly suggest that the CNS is particularly sensitive to eIF2B dysfunction.

### VWM mutations in 2 distinct eIF2B subunits elicit similar transcriptional changes in the CNS.

Given the phenotypic similarities between VWM disease mouse models, we sought to profile the transcriptome to identify common dysregulated pathways. To this end, we performed bulk RNA-seq on the cerebellum from 4-month-old N208Y^HOM^ mice before and after drug removal, as well as from untreated 5-month-old R191H^HOM^ mice. As expected, 2BAct did not affect gene expression in WT mice (Supplemental Figure 6A). However, we found 710 differentially expressed genes (DEGs) in the cerebellum of 2BAct-treated N208Y^HOM^ (log_2_ fold change > 0.5 and *q* < 0.05) and 210 DEGs in R191H^HOM^ relative to their respective WT controls (Supplemental Table 3). Fold changes of DEGs in either R191H^HOM^ or 2BAct-treated N208Y^HOM^ were well correlated (*P* = 7.40 × 10^–240^, R^2^ = 0.65, Supplemental Figure 6B). ISR target genes were upregulated in both mutant mice, but the effect sizes trended higher in 2BAct-treated N208Y^HOM^ mice (Supplemental Figure 6B red circles, and Supplemental Figure 6C, compare 1st and 2nd columns), consistent with its faster disease progression.

Withdrawal of 2BAct further increased expression of the ISR CLIC signature in N208Y^HOM^ mice (*P*_adj_ = 0.0013, Supplemental Figure 6C compare 2nd and 3rd columns, and Supplemental Table 4), and led to differential expression of many additional genes (*n* = 2,958 with *q* < 0.05, Supplemental Figure 6D and Supplemental Table 3) and gene sets (Supplemental Figure 6, E and F, and Supplemental Table 5, Materials and Methods). Gene sets that were upregulated in all 3 comparisons with WT (R191H^HOM^, 2BAct-maintained N208Y^HOM^, and 2BAct-withdrawn N208Y^HOM^) related to ribosome biogenesis, 3′UTR mediated translational regulation and rRNA metabolic process (Supplemental Figure 6E and Supplemental Table 5), and downregulated gene sets related to neuronal synapse structure or function. These pathways likely represent ISR-driven changes that underpin a similar phenotypic decline.

Unique gene sets that were significantly changed only in 2BAct-withdrawn N208Y^HOM^ included downregulation of oxidative phosphorylation, respiratory electron transport, and gluconeogenesis (Supplemental Figure 6F, Supplemental Table 5), suggesting that more pronounced ISR activation leads to reduction of key energetic functions in brain cells. This broad transcriptional response in the CNS, together with rapid activation of the ISR in all peripheral tissues, drives the rapid physiological decline observed upon drug removal.

### Single nuclei transcriptomic analysis revealed that oligodendrocytes, astrocytes, and ependymal cells are prone to ISR activation in both VWM models.

**Figure 5:**
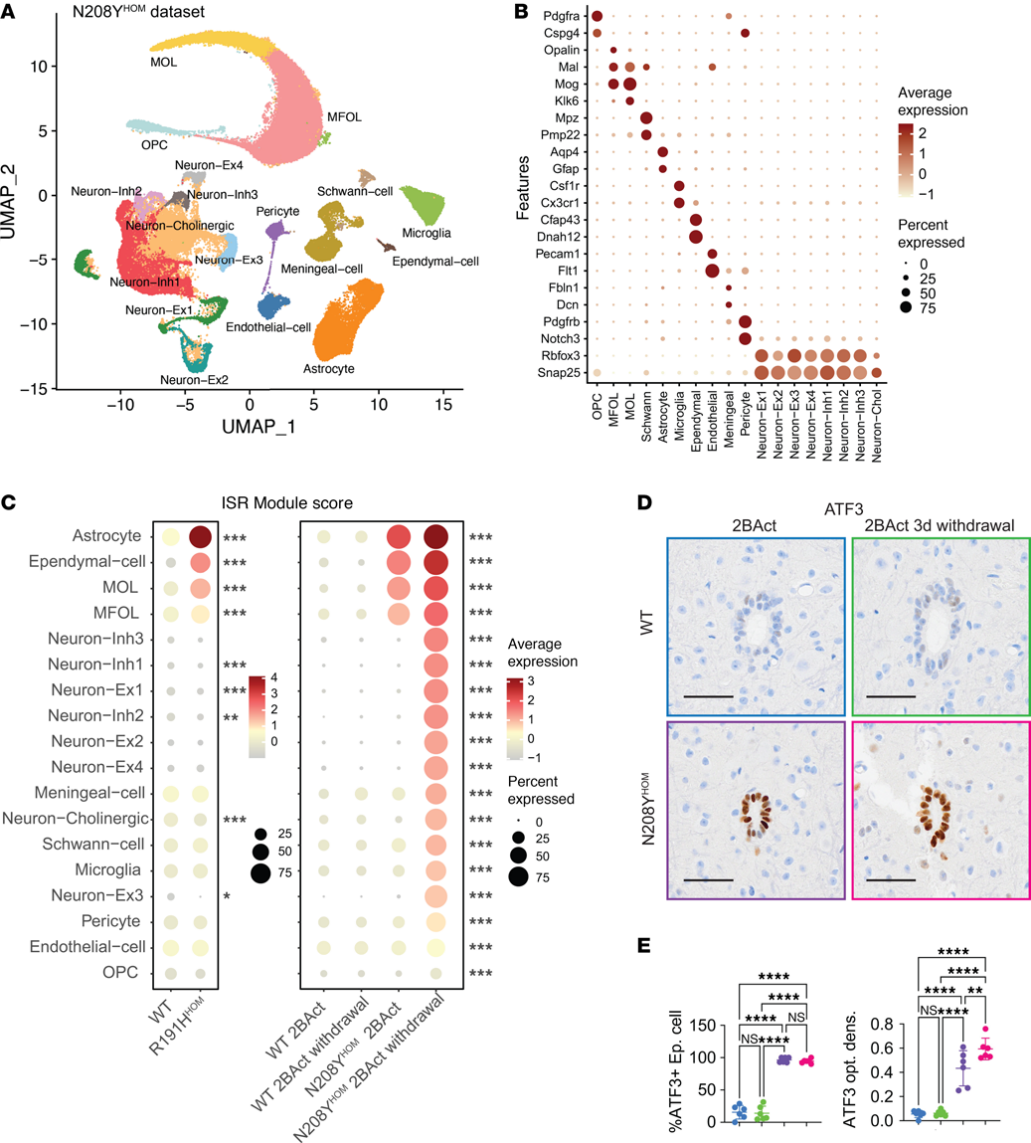
Single nuclei RNA-seq of spinal cord identifies common ISR-susceptible cell types in R191H^HOM^ and N208Y^HOM^ mouse models. (**A**) Uniform manifold approximation and projection (UMAP) embedding of 115,289 single nuclei isolated from cervical thoracic spinal cord of male, 3-month-old WT and N208Y^HOM^ mice maintained on 2BAct or 3 days after withdrawal (*n* = 3 per group). Colors indicate the 18 clusters identified through graph-based clustering. OPC, oligodendrocyte progenitor cell; MFOL, myelin-forming oligodendrocytes; MOL, mature oligodendrocytes; Ex, excitatory; Inh, inhibitory; Chol, cholinergic. (**B**) Dot plot expression of marker genes for major cell types in the spinal cord from the N208Y dataset. The color intensity represents average expression for each cluster and the size of dot shows the percent of nuclei in each cluster expressing the gene. (**C**) Dot plot of ISR CLIC module score expression across cell types in untreated WT and R191H^HOM^ mice and WT and N208Y^HOM^ mice maintained on 2BAct or 3 days after withdrawal. Cell types are ordered from most to least abundant. Kruskal-Wallis test. (**D**) Representative IHC images of ATF3 staining around the central canal of the thoracic spinal cord from 4-month-old male mice. WT +2BAct, *n* = 6; WT withdrawn, *n* = 6; N208Y^HOM^ +2BAct, *n* = 6; N208Y^HOM^ withdrawn, *n* = 6. Scale bar: 50 μm. (**E**) Quantification results of **D** as percent of ependymal cells that are ATF3-positive (left) and ATF3 optical density (right). One-way ANOVA, Holm-Šídák post hoc. *P < 0.05, **P < 0.01, ***P < 0.001, ****P < 0.0001.

To further understand the vulnerability of the CNS toward chronic ISR activation and to reveal cell type–specific responses to pathway induction, we performed single nuclei RNA-seq (snRNA-seq) on spinal cords of 3-month-old WT and N208Y^HOM^ mice maintained on 2BAct or 3 days after drug withdrawal. Spinal cords were chosen, since striking demyelination occurs in this organ in VWM models and this tissue provides a more comprehensive cell type representation than neuron-enriched cerebellar tissue. After filtering low-quality nuclei and doublets, we grouped 115,289 nuclei into 18 transcriptional clusters using graph-based clustering and visualized them in low dimensional space with UMAP (Figure 5A). We annotated clusters based on the expression of well-established cell type markers (Figure 5B) (29, 30), and identified oligodendrocyte progenitor cells (OPC), myelin-forming oligodendrocytes (MFOL), mature oligodendrocytes (MOL), Schwann cells, astrocytes, microglia, ependymal cells, endothelial cells, meningeal cells, pericytes, and 8 neuronal clusters further defined by neurotransmitter marker genes (Supplemental Figure 7C). In addition, we performed snRNA-seq on spinal cords from 2.5-month-old, untreated WT and R191H^HOM^ animals and recapitulated the same 18 transcriptional clusters from 50,731 post-QC nuclei (Supplemental Figure 7, A–C) via projection onto the N208Y^HOM^ UMAP structure.

To assess ISR activation levels in cells of each cluster, we used the ISR CLIC gene set to calculate an ISR module score at the single-cell level. In line with previous analyses (13), astrocytes and oligodendrocytes displayed upregulation of the ISR in the spinal cord of R191H^HOM^ mice (Figure 5C). In addition, we identified ependymal cells (ciliated epithelial glia that line the central canal) as another cell type with increased ISR expression in this VWM model. Notably, the same cell types (astrocytes, both MFOLs, MOLs, and ependymal cells) exhibited an enhanced ISR in 2BAct-maintained N208Y^HOM^ mice compared with WT mice (Figure 5C), revealing the cell types culpable for the residual CNS ISR. Upon 3 days of drug withdrawal, the ISR pathway was further increased in these cell types and activated in almost all other spinal cord cell types (Figure 5C). The ability of 2BAct to fully suppress the ISR in most cell types, including all neuronal subtypes, further corroborates adequate drug exposure in the CNS and identifies four glial clusters as preferentially susceptible to ISR activation when eIF2B activity is diminished. Intriguingly, the specific genes significantly upregulated upon ISR activation differed across clusters in N208Y^HOM^ mice (Supplemental Figure 8, A and B). This is indicative of cell type specificity in the transcriptional program engaged upon pathway activation and could have functional implications in how different CNS cell types adapt to or malfunction upon stress.

To validate the transcriptomic findings in the newly identified ISR-prone ependymal cells, we performed IHC of the ISR-induced transcription factor ATF3 in the spinal cord of WT and N208Y^HOM^ mice maintained on 2BAct or 3 days after drug withdrawal. N208Y^HOM^ mice, both maintained on or withdrawn from 2BAct, displayed a significant increase in both the percentage of ependymal cells that were ATF3 positive and the optical density of the staining (Figure 5, D and E). The staining intensity further increased when N208Y^HOM^ animals were withdrawn from 2BAct (Figure 5E).

snRNA-seq analyses thus allowed us to identify astrocytes, oligodendrocytes, and ependymal cells as the main drivers of the CNS ISR in both VWM disease models, pointing toward a common mechanism leading to demyelination and motor deficits in 2BAct-treated N208Y^HOM^ and untreated R191H^HOM^ mice.

### N208Y^HOM^ mice exhibit dysfunctional oligodendrocyte maturation.

We next sought to assess how a chronic ISR could impact the cell type composition in the spinal cord and how further upregulation of this pathway upon 2BAct withdrawal could affect the function of various cell types. To this end, we visualized the UMAP by genotype and 2BAct treatment and observed that cells from WT and N208Y^HOM^ mice occupied different neighborhoods within some clusters, suggesting a transcriptional state shift. This was most evident for MFOLs, where a sizable portion of cells isolated from N208Y^HOM^ mice occupied a neighborhood space relatively devoid of WT cells (Supplemental Figure 9A, circles). Similarly, MOLs from both N208Y^HOM^ groups were also shifted compared with WT groups (Supplemental Figure 9A, rectangles). Cell type composition was assessed across groups (Supplemental Figure 9, B and C), and since MOLs were the only cell type in which relative proportions significantly differed (Supplemental Figure 9C, 1-way ANOVA), we focused on the oligodendrocyte lineage in the N208Y cohort for deeper analysis. Of note, no differences in cell type composition were detected in the milder R191H model compared with WT animals (Supplemental Figure 9, D and E).

**Figure 6:**
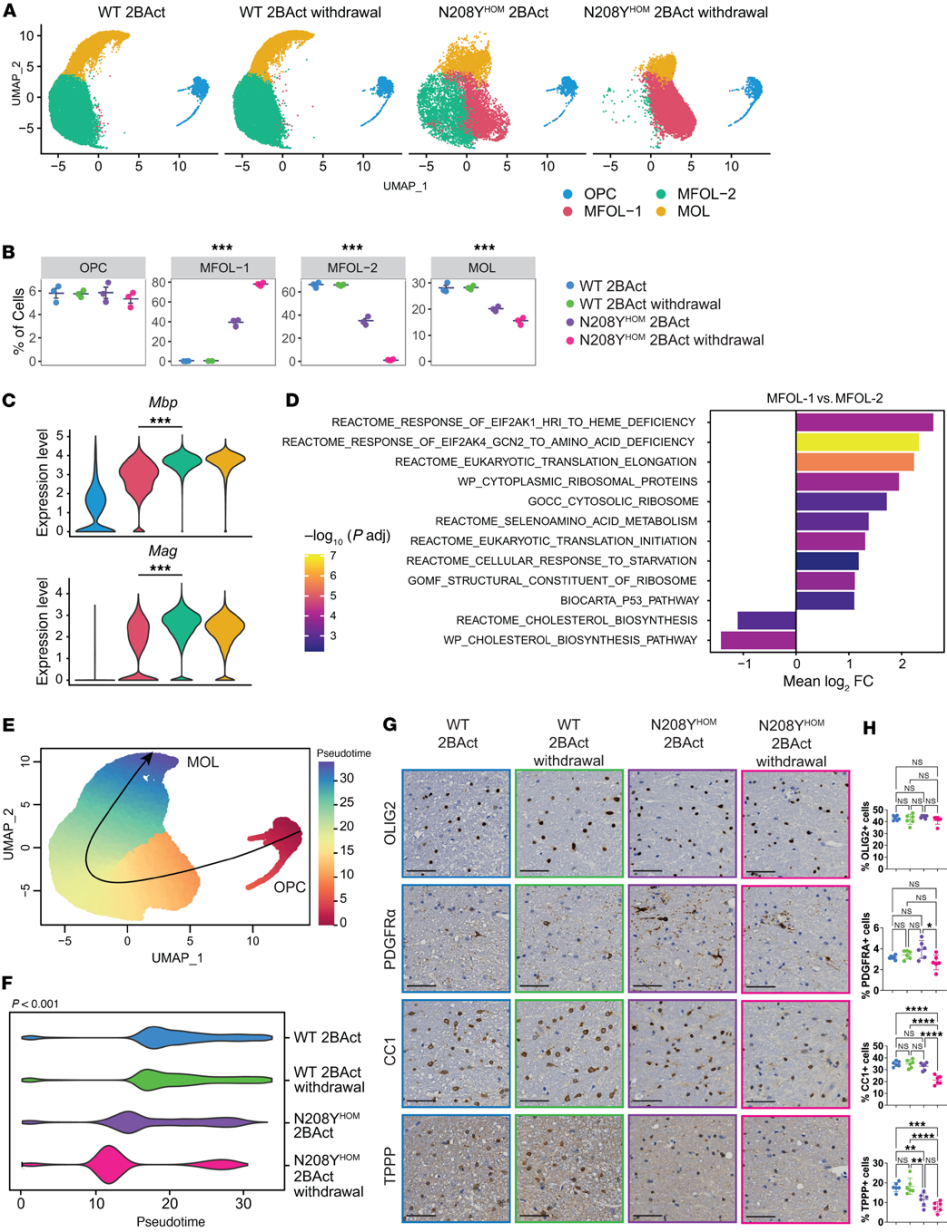
Oligodendrocyte maturation trajectory and cholesterol biosynthesis are affected in the spinal cord of N208Y^HOM^ mice. (**A**) UMAP of nuclei from the oligodendrocyte lineage from **C**, split by genotype and treatment. Colors indicate the 4 clusters identified after reclustering. OPC, oligodendrocyte precursor cell; MFOL, myelin-forming oligodendrocyte; MOL, mature oligodendrocyte. (**B**) Proportions of cell clusters after reclustering the oligodendrocyte lineage cells. One-way ANOVA. (**C**) Violin plots showing the expression levels of mature oligodendrocyte markers in reclustered cells. Wilcoxon Rank Sum test (MFOL-1 versus MFOL-2). (**D**) Gene set enrichment analysis of MFOL-1 versus MFOL-2 clusters. (**E**) Pseudotime visualization of oligodendrocyte lineage cells, with points colored by pseudotime and a principal curve used to represent the inferred trajectory of differentiation. (**F**) Violin plot showing the distribution of cells in each group along the pseudotime trajectory. Kruskal-Wallis test, *P* <0.001. (**G**) Representative IHC images of OLIG2, PDGFR-α, CC1, and TPPP staining in the spinal cord of 4-month-old male mice. WT +2BAct, *n* = 6; WT withdrawn, *n* = 6; N208Y^HOM^ +2BAct, *n* = 6; N208Y^HOM^ withdrawn, *n* = 6. Scale bar: 50 μm. (**H**) Quantification results of **G** as percent of positive cells among all cells in cervical and thoracic spinal cords. One-way ANOVA, Holm-Šídák post hoc. *P < 0.05, **P < 0.01, ***P < 0.001, ****P < 0.0001.

To enhance cell subtype resolution, we subsetted our data to the oligodendrocyte lineage cell types and performed reclustering. This analysis revealed four distinct populations: OPCs, 2 populations of myelin-forming oligodendrocytes (MFOL-1 and MFOL-2), and MOLs (Figure 6A), with differences between groups observed in the proportions of MFOL-1, MFOL-2, and MOL cells (Figure 6B, 1-way ANOVA). The enrichment of the MFOL-2 population in WT compared to N208Y^HOM^ mice suggests that this represents a healthy oligodendrocyte population important for normal physiology. 2BAct-withdrawal strongly decreased this population in mutant mice (Figure 6B). In contrast, the MFOL-1 population was only observed in N208Y^HOM^ mice and became the predominant subtype upon 2BAct-withdrawal (Figure 6B). As expected, the majority of ISR CLIC genes were expressed at significantly higher levels in MFOL-1 compared with MFOL-2 cells (Supplemental Figure 10A). Genes critical for myelin sheath formation (such as *Mbp* and *Mag)* were significantly downregulated in MFOL-1 cells (Figure 6C), suggesting that this population of N208Y^HOM^-associated oligodendrocytes is dysfunctional in production and/or maintenance of myelin. We then performed unbiased differential expression analysis on MFOL-1 versus MFOL-2 clusters to evaluate how ISR-high oligodendrocytes enriched in mutant mice differ from ISR-low cells enriched in WT mice. We identified 1,321 significantly upregulated genes and 1,094 significantly downregulated genes in MFOL-1 compared with MFOL-2 clusters (log_2_ fold change > 0.5 and FDR < 0.05) (Supplemental Table 6). GSEA identified an expected upregulation of ISR-related gene sets and a notable downregulation of genes involved in cholesterol biosynthesis in N208Y^HOM^- enriched MFOL-1 cells (Figure 6D), a critical process for myelin formation in the CNS (31). Similarly, this pathway was also downregulated in MFOLs from untreated R191H^HOM^ mice compared with WT mice (Supplemental Figure 10B), indicating the contribution of cholesterol metabolism to VWM etiology.

MOLs were detected at lower frequency in both 2BAct-maintained and -withdrawn N208Y^HOM^ spinal cords (Figure 6B), indicative of a potentially defective maturation trajectory that could contribute to observed spinal cord phenotypes. We thus inferred a lineage structure from OPCs to MOLs using the Slingshot algorithm (Figure 6E) to assess whether oligodendrocyte lineage cells from N208Y^HOM^ spinal cords might occupy distinct states along a maturation continuum compared with WT cells. Interestingly, beyond the OPC state (where cell proportions did not differ between groups and there was no detectable ISR), cells from 2BAct-maintained N208Y^HOM^ mice were found earlier in the trajectory compared with WT cells and did not reach the furthest points of the maturation continuum (Figure 6F). After 3 days of withdrawal of 2BAct in N208Y^HOM^ mice, we observed a bimodal distribution of cells along the trajectory, with the highest density of cells closer to the progenitor stage and a more severe loss of the most mature oligodendrocytes. To validate this finding, we performed IHC for well-established oligodendrocyte lineage markers, including OLIG2 (a pan-oligodendrocyte lineage marker), PDGFR-α (an OPC marker), and CC1 (a marker for myelin-forming and mature oligodendrocytes) (32), as well as Tubulin Polymerization Promoting Protein (TPPP), a Golgi outpost protein specifically expressed in myelinating oligodendrocytes (Figure 6, G and H) (33, 34). The ratio of OLIG2-positive oligodendrocytes relative to total cells remained constant across genotypes and regardless of 2BAct availability. Similarly, the percentage of PDGFR-α–positive cells was consistent across genotypes, although a slight decrease was observed upon 2BAct withdrawal in N208Y^HOM^ mice. Interestingly, while the ratio of CC1-positive myelin-forming and mature oligodendrocytes remained unchanged in WT mice regardless of 2BAct availability, it significantly decreased in N208Y^HOM^ mice following 2BAct withdrawal. Moreover, the ratio of TPPP-positive myelinating oligodendrocytes was significantly reduced in N208Y^HOM^ mice compared to WT, with a downward trend observed upon 2BAct withdrawal (Figure 6, G and H). Together, these results suggest that ISR induction impairs the normal progression of oligodendrocyte maturation.

To further investigate changes in the transcriptome, we conducted differential gene expression analysis on all cell types identified in the N208Y^HOM^ dataset (Supplemental Figure 10C). Astrocytes, MFOLs, and MOLs (cell types that had an ISR in the 2BAct-maintained group) displayed the largest number of DEGs in all 3 comparisons, suggesting a drastic rewiring of the transcriptome in cells that have a chronic stress response. GSEA of the comparison with the most DEGs (2BAct-withdrawn N208Y^HOM^ versus 2BAct-maintained WT) identified the unfolded protein response (UPR), Myc targets, oxidative phosphorylation, and cholesterol homeostasis as the common Hallmark gene sets differentially expressed across many cell types (Supplemental Figure 10D; see Supplemental Table 7 for full list of comparisons and gene sets tested). Upregulation of the UPR and Myc targets is not surprising, as they share many genes with the ISR CLIC gene list. Specific interrogation of the ATF6 and IRE1 arms of the UPR (35) in bulk spinal cord tissue did not show significant gene set enrichment for these pathways in 2BAct-withdrawn N208Y^HOM^ compared to 2BAct-maintained WT mice (Supplemental Table 8). In addition to MFOLs, MOLs, astrocytes, and several types of neurons also exhibited a decrease in genes related to cholesterol homeostasis, suggesting a common downregulation of this pathway by the ISR across cell types. Unregulated disruption of de novo cholesterol synthesis may prompt grave consequences for the CNS, the body’s most cholesterol-rich organ (36).

### ISR activation decreases cholesterol synthesis, leading to a reduction of free cholesterol in the CNS.

**Figure 7:**
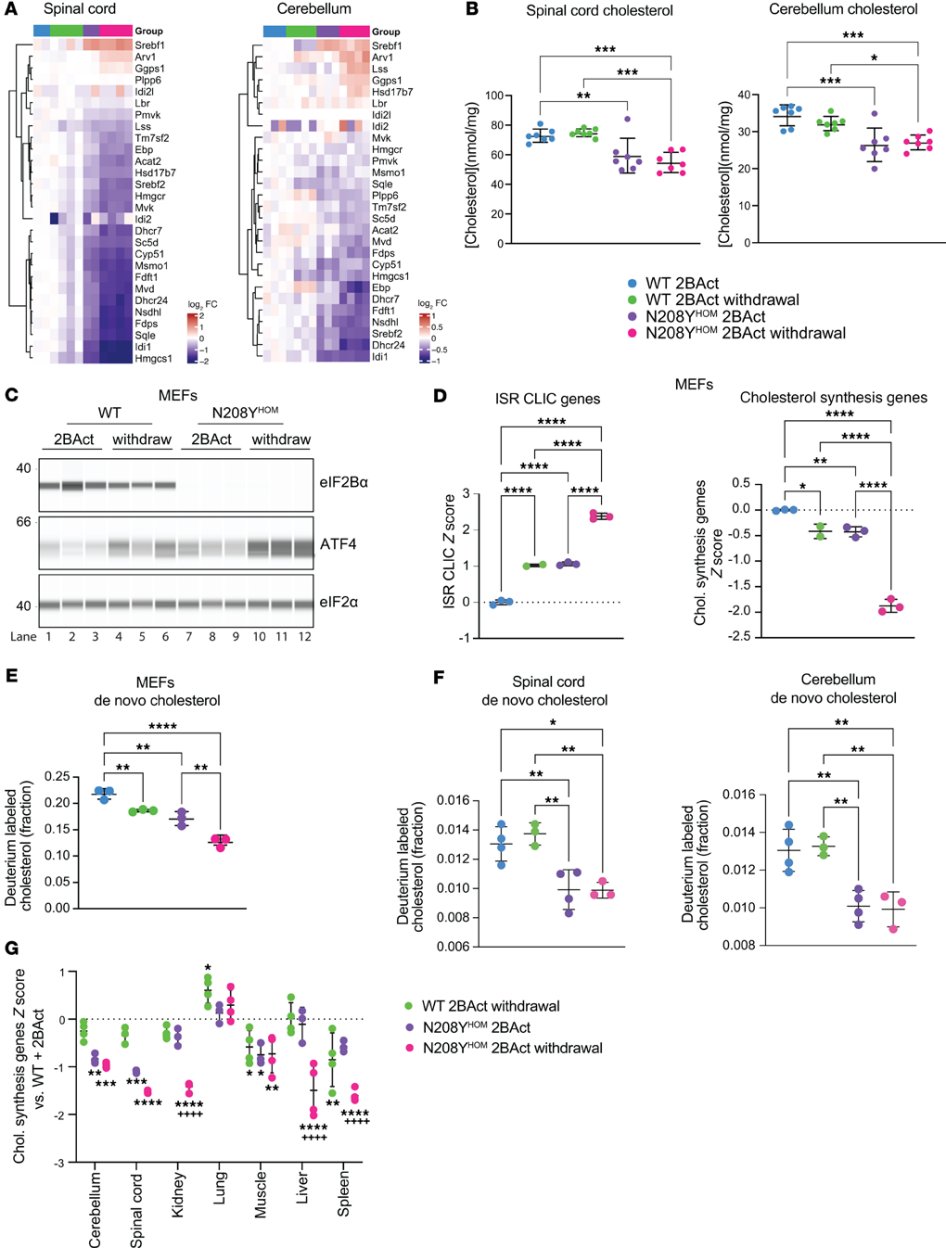
De novo cholesterol synthesis is reduced in MEFs and CNS tissues from N208Y^HOM^ mice. (**A**) Heatmap of cholesterol biosynthesis gene expression in spinal cord and cerebellum of 4-month-old male WT and N208Y^HOM^ mice on 2BAct or 3 days after withdrawal. Values are log_2_ fold change versus WT +2BAct. (**B**) Total free cholesterol in spinal cord and cerebellum of 5-month-old female WT or N208Y^HOM^ mice on 2BAct or after 2-day withdrawal. WT +2BAct, *n* = 7; WT +2BAct withdrawal, *n* = 7; N208Y^HOM^ +2BAct, *n* = 7; N208Y^HOM^ 2BAct withdrawal, *n* = 7. One-way ANOVA, Tukey post hoc. (**C**) Immunoblot for eIF2B-α, ATF4 and eIF2-α of WT or N208Y^HOM^ MEFs with 2BAct or after 24-hour withdrawal. (**D**) Average z-score of ISR CLIC genes (left) and cholesterol biosynthesis genes (from 7A, right) calculated from bulk RNA-seq analysis of WT or N208Y^HOM^ MEFs with 2BAct or after 6-hour withdrawal. *n* = 2–3, error bars represent SD. One-way ANOVA, Holm-Šídák post hoc. (**E**) Deuterium labeling of de novo synthesized cholesterol in WT or N208Y^HOM^ MEFs with 2BAct or after 24-hour withdrawal. Bars represent the sum of all ^2^H-incorporating isotopologues beyond natural abundance, error bars represent SD. *n* = 3. One-way ANOVA, Tukey post hoc. (**F**) Deuterium labeling of de novo synthesized cholesterol from spinal cord and cerebellum of WT (*n* = 4) or N208Y^HOM^ mice (*n* = 3) on 2BAct or after 3-day withdrawal. Bars represent the sum of all ^2^H-incorporating isotopologues beyond natural abundance, error bars represent SD. One-way ANOVA, Tukey post-hoc. (**G**) Average z-score of cholesterol biosynthesis genes in multiple tissues from 4-month-old male N208Y^HOM^ +2BAct mice or WT and N208Y^HOM^ mice following 2BAct withdrawal, normalized to WT +2BAct littermates. Two-way ANOVA, Holm-Šídák test. ^++++^*P* < 0.0001 for comparison between N208Y^HOM^ 2BAct maintained and withdrawal. *P < 0.05, **P < 0.01, ***P < 0.001, ****P < 0.0001 for all other comparisons. Error bars are SD.

In aggregate, the contribution of CNS susceptible cell types to impaired cholesterol regulation can be detected at the bulk RNA-seq level in both spinal cord and cerebellum of N208Y^HOM^ mice as a significant downregulation of cholesterol biosynthesis genes. This decrease is observed in the 2BAct-maintained state, characterized by a chronic ISR, and further reduced upon drug withdrawal (Figure 7A and Supplemental Table 9). We used mass spectrometry to quantify tissue cholesterol abundance and found a significant reduction in total free cholesterol in the spinal cord and cerebellum of 2BAct-maintained N208Y^HOM^ mice (Figure 7B), reflecting the continuous impairment of this biosynthetic pathway caused by lifelong ISR activation in the CNS. Free cholesterol was not further decreased after a 3-day drug washout, suggesting that acute downregulation of synthesis genes is not sufficient to further impact steady-state cholesterol levels in slow-turnover tissues such as the CNS (31).

To determine whether the ISR reduced de novo cholesterol synthesis, we generated primary mouse embryonic fibroblasts (MEFs) from WT and N208Y^HOM^ mice and measured the incorporation of deuterated water into newly synthesized cholesterol by LC-MS. Similar to MIN6 cells, N208Y^HOM^ MEFs had drastically reduced levels of eIF2B-α (Figure 7C), and increased ATF4 and ISR CLIC gene expression in the presence of 2BAct (Figure 7D and Supplemental Table 9). As seen in the transcriptomic analysis of the spinal cord, BiP, a UPR target, was not upregulated at the protein level in N208Y mutant cells nor was the negative regulator GADD34, both of which were induced by the ER stressor thapsigargin (Supplemental Figure 10E). Drug withdrawal further increased ISR induction and elicited downregulation of cholesterol synthesis genes in both WT (which have a basal ISR) and N208Y^HOM^ MEFs (Figure 7, C and D, calculated as an average z-score of genes in Figure 7A). Notably, deuterium labeling of cholesterol was significantly reduced in 2BAct-maintained N208Y^HOM^ MEFs as compared with 2BAct-maintained WT MEFs, and labeling was further reduced upon drug washout in both genotypes (Figure 7E), consistent with a reduction in de novo cholesterol synthesis upon ISR activation.

To assess whether the ISR suppresses cholesterol synthesis in vivo, we measured the incorporation of deuterium into cholesterol in the central nervous system of WT and N208Y^HOM^ mice. Mice received 6% deuterium-enriched water in drinking water for 14 days before tissue collection. Consistent with the finding in MEF cells, N208Y^HOM^ mice, both those maintained on 2BAct and those with 3 days of drug withdrawal, showed significantly reduced labeled cholesterol in the spinal cord and cerebellum compared with the WT controls (Figure 7F). Due to the slow turnover of cholesterol in the adult mouse brain (37, 38), the effects of 3 days of 2BAct withdrawal would be expected to be relatively modest in the CNS, consistent with these findings.

As acute ISR activation in peripheral organs upon drug removal is a feature of the N208Y^HOM^ model, we expanded our analysis of cholesterol biosynthetic enzyme transcripts to organs outside the CNS and found that downregulation of this pathway, calculated as an average z-score of genes in Figure 7A, was also observed in the kidney, liver, and spleen of N208Y^HOM^ mice upon 3 day drug withdrawal compared with 2BAct-maintained counterparts (Figure 7G). This suggests that rewiring of cholesterol homeostasis may be a ubiquitous metabolic output of ISR pathway activation, though organs such as the CNS that cannot rely on dietary uptake may be particularly susceptible to tissue dysfunction upon chronic ISR upregulation.

In summary, our results revealed that decreased expression of cholesterol synthesis enzymes is a transcriptional ISR output shared by the CNS and many peripheral tissues. In the CNS, chronic disruption of this biosynthetic pathway led to a reduction in de novo synthesis rates and total free cholesterol in N208Y^HOM^ mice, which is likely a key contributor to dysfunctional oligodendrocyte maturation, reduced myelination, and ensuing VWM etiology.

## Discussion

eIF2B is a stress sensor and effector of the ISR in all eukaryotic cells. Here, we determined the cellular and in vivo consequences of abolishing metabolite binding by generating cells and a mouse model carrying the N208Y eIF2B-α VWM-associated mutation. In both, we found a striking reduction in eIF2B-α levels, an essential regulatory subunit that, like 2BAct, promotes eIF2B decamer formation. The destabilizing impact of the analogous sugar phosphate binding mutation, N209Y, has also been observed in yeast (39).

To date, VWM mouse models have displayed CNS-restricted ISR activation and pathology, but we discovered that introduction of the N208Y eIF2B-α mutation elicits widespread stress induction that results in neonatal lethality. This early and severe phenotype is akin to early onset human VWM cases that present with antenatal and neonatal lethality. Apart from reduced body weight, we found no obvious defects in gross organ development in E18.5 N208Y^HOM^ embryos, a surprising result, given the proposed role of the ISR in cell fate determination (40–42). A deeper investigation of the maintenance of cell states during development could reveal a role for the ISR in differentiation and illuminate the cause of death of N208Y^HOM^ pups. Birth, a stressful event, likely leads to a spike in phosphorylation of eIF2, further inhibiting eIF2B in a system where its activity is already compromised.

The remarkable ability of 2BAct to rescue neonatal lethality and significantly extend lifespan demonstrates that its unique mechanism, a molecular stapler of the eIF2B complex, can sufficiently boost its residual activity in vivo. This result, combined with the complete rescue of VWM pathology in R191H^HOM^ mice (13), and enhancement of GEF activity of all mutations tested in vitro (10), underscores the efficacy that complex stabilization and activation provide in a disease driven by eIF2B hypomorphic alleles. Fosigotifator is an analog that is currently under investigation for the treatment of VWM disease (ClinicalTrials.gov NCT05757141). Fosigotifator is likely to have broad applicability across disease severity spanning pediatric disease, which has the highest incidence, to adolescent and adult-onset disease, which are the most prevalent, due to their slower progression. This is an important property, given the rarity of VWM disease.

Comprehensive profiling of 2 VWM mouse models with hypomorphic mutations in distinct subunits and level of dysfunction allowed us to identify vulnerable CNS cell types and mechanistic drivers of disease with high confidence. The phenotypic similarity of untreated R191H^HOM^ and 2BAct-maintained N208Y^HOM^ mice extended to their CNS bulk transcriptomes. Furthermore, molecular analysis of the spinal cord from both models at a single cell level revealed a striking shared cell type susceptibility, with astrocytes, oligodendrocytes, and ependymal cells being most prone to eIF2B dysfunction and chronic ISR activation. This is, to our knowledge, the first time that ependymal cells have been identified as exquisitely sensitive in VWM models of disease. Their localization at the CSF-neural tissue interface, as well as expression of glucose, fructose, metal ion, and water transporters (43, 44), confer them the unique capability to detect and bidirectionally regulate brain solutes and clear waste. How dysregulation of the nutrient sensing and metabolic regulation capacity of ependymal cells specifically relates to VWM pathology remains to be studied.

2BAct initially suppressed the ISR in both CNS and peripheral tissues of N208Y^HOM^ E18.5 embryos, rescuing their birth weight. However, continued availability of the drug did not sufficiently dampen the stress response in the CNS, and they eventually developed pathology, indicating onset of vulnerability to eIF2B dysfunction between late embryonic development and early adulthood. This period encompasses the generation of astrocytes from radial glial cells, the maturation of ependymal cells, generation of oligodendrocytes, and the early and peak phases of postnatal myelination (45–47). This time period also encapsulates the appearance of the ISR in untreated R191H^HOM^ mice. Thus, the inability of 2BAct to quell CNS ISR activation coincides with the appearance of the cell types we identified in both R191H and N208Y models as ISR prone. The reason for their susceptibility to reduced eIF2B function is not yet known, but may be due to unique metabolic states that impact complex stability and activity, and high synthetic burden.

Our single cell trajectory analysis also revealed that oligodendrocytes are in a less mature state which is consistent with findings in VWM patients (48). An increase in PDGFRα-positive OPCs in the white matter was reported in patients but we did not detect differences at this early stage of the oligodendrocyte lineage in N208Y^HOM^ mice. Whether the stunting along the maturation trajectory observed in N208Y^HOM^ mice is due to a stall in differentiation or preferential death of MOLs or a combination of both remains to be explored. Our results suggest that the inability of ISR-high oligodendrocytes to fully mature and the downregulation of cholesterol biosynthesis likely contribute to hypomyelination in VWM disease. De novo synthesis of cholesterol, a major constituent of myelin, is of critical importance in the CNS, which cannot uptake peripheral sources due to the blood brain barrier (31, 49). Thus, ISR-induced downregulation of cholesterol biosynthesis may confer a unique susceptibility to an organ that cannot otherwise obtain extrinsic sources of cholesterol for proper function. Moreover, the turnover of cholesterol in the CNS is very slow, which could account for delayed kinetics of disease onset (50).

Drug withdrawal elicited an ISR in nearly all CNS cell types. Phospho-eIF2-α–dependent repression of global translation leads to a decline in synaptic proteins and neuronal death in prion-diseased mice (51); therefore, synaptic transmission failure could be one mechanism contributing to the rapid system-wide physiological decline of N208Y^HOM^ mice when 2BAct is withdrawn. Indeed, we observed downregulation of synapse-related pathways in neuron clusters (Supplemental Table 7) and oxidative phosphorylation pathways in microglia and certain neuron subtypes (Supplemental Figure 10D). Cholesterol biosynthesis emerged as a shared pathway downregulated in multiple cell types and corroborated by free cholesterol and in vivo labeling measurements in CNS tissues, the most cholesterol-rich organ in the body. Cholesterol is an important structural component of cell membranes, a precursor of bioactive molecules such as steroid hormones and oxysterols, essential for synapse formation and function, and defects in its metabolism have been implicated in neurological disease reviewed in refs. 52–54. An inverse relationship between ISR activation and sterol synthesis has been previously described and thought to be regulated at the level of SREBP cleavage (55). Our findings in CNS tissues in vivo show downregulation at the transcript level of the master regulator of cholesterol metabolism Srebf2 (Figure 7A). The suppression of cholesterol synthesis enzymes also extended to peripheral tissues when drug was withdrawn, indicating that this is a common metabolic response when the ISR is engaged.

Understanding how ISR activation impacts various pathways and determining their contribution to physiology across cellular contexts is an important next step. ISR induction, including in N208Y MEFs in culture, was shown to be sufficient to remodel the metabolome, increasing levels of several amino acids and reducing the flux through the TCA cycle and mitochondrial respiration (6, 56). Pathway activation also broadly remodeled the lipidome and triggered lipid droplet biogenesis. These core outputs of the ISR are likely contributors to pathological states when persistently engaged, as is the case in VWM disease. Given that a wide variety of insults converge on eIF2-α phosphorylation and activation of the ISR, elucidating the impact of this stress response will reveal mechanisms of pathogenesis that are likely relevant to other diseases characterized by its chronic upregulation. The beneficial effect of pharmacological ISR attenuation has been reported in mouse models of 2 other leukodystrophies and many other pathological contexts (7, 57–61). While this preclinical study has some limitations, including the translatability of the N208Y mouse model and the static nature of molecular and IHC snapshots capturing oligodendrocyte maturation, it provides valuable insights into the cellular and organismal effects of the ISR. Whether these outputs of the ISR, such as cholesterol and lipid dysregulation, contribute to cerebrovascular diseases and other early onset or age-related white matter diseases remain another important therapeutic question (62–65).

The N208Y^HOM^ mouse described here provides a unique opportunity to temporally control activation of the ISR in vivo, without engagement of parallel pathways, by tuning the availability of 2BAct. It provides a genetic system capable of systemic ISR activation, thus expanding the tissues in which the effect of this stress response can be studied. Further analyses at the single-cell level across various time points and across tissues will help to elucidate components of the complex transcriptional network underlying the ISR. These additional analyses may also clarify the mechanism(s) underlying cell-type specific vulnerability to the ISR.

## Methods

### Sex as a biological variable.

Our study examined male and female animals, and similar findings are reported for both sexes.

### Statistics.

Prior to statistical testing, the normality of the data distribution for each group was assessed using Kolmogorov-Smirnov or Shapiro-Wilk (for smaller sample sizes) tests, with the F or Brown-Forsythe tests used to test for homoscedasticity. Student’s *t* test was used for comparisons between 2 groups for normally distributed data. For data with unequal variances, a Welch’s *t* test was used instead. One-way ANOVA with Holm-Šídák or Tukey post hoc comparisons was used for comparisons between 3 or more groups with normal distributions; otherwise, the Kruskal-Wallis test with Dunn’s post hoc comparisons was used for nonnormal distributions. *P* values less than 0.05 were considered significant.

### Study approval.

All animal experiments and methods in this manuscript were approved by the Institutional Animal Care and Use Committee of Calico Life Sciences, LLC. and Taconic Biosciences, Inc.

### Data availability.

All raw data from RNA-seq experiments are available with no restrictions in ArrayExpress (https://www.ebi.ac.uk/biostudies/arrayexpress) (E-MTAB-15246, E-MTAB-15247, E-MTAB-15273, E-MTAB-15295, E-MTAB-15300). Supplemental Methods, Figures, Tables, and Supporting data values—including all quantitative data points used in graphs—are provided in the Supplemental Materials.

## Author contributions

KL, NL, CFC, JJL, and CS wrote the original draft of the manuscript. KL, JJL, and CS conceptualized the project. KL, NL, VS, and JJL developed the methodology. KL, NL, RBK, NV, BK, HMR, JS, QH, JZS, CZ, VS, CFC, and JJL performed the investigation. KL, NL, CFC, and JJL visualized the project. EGM, QH, NVB, BDB, JJL, and CS supervised the project. KL, NL, CFC, JJL, and CS wrote the original draft of the manuscript.

## Supplementary Material

Supplemental data

Unedited blot and gel images

Supplemental table 1

Supplemental table 2

Supplemental table 3

Supplemental table 4

Supplemental table 5

Supplemental table 6

Supplemental table 7

Supplemental table 8

Supplemental table 9

Supporting data values

## Version 1 07/17/2025

In-Press Preview

## Version 2 08/22/2025

Electronic publication
