# Supplementary material for: Chronic integrated stress response causes dysregulated cholesterol synthesis in white matter disease
Source: JCI Insight. 2025 Jul 15;10(16):e188459. doi: 10.1172/jci.insight.188459 (PMC12406721; doi:10.1172/jci.insight.188459)
Supplement: Supplemental data [file jciinsight-10-188459-s208.pdf]

SUPPLEMENTARY MATERIALS

Chronic integrated stress response causes dysregulated cholesterol synthesis in white matter disease

Karin Lin, Nina Ly, Rejani B. Kunjamma, Ngoc Vu, Bryan King, Holly M. Robb, Eric G. Mohler, Janani Sridar, Qi Hao, José Zavala-Solorio, Chunlian Zhang, Varahram Shahryari, Nick van Bruggen, Caitlin F. Connelly, Bryson Bennett, James J. Lee, Carmela Sidrauski

Supplementary Figures S1- S10

Supplementary Tables S1- S9 (excel files attached separately, titles listed below)

Supplementary Key Resources Table

Supplementary Methods

Supplementary References

Supplementary Figures

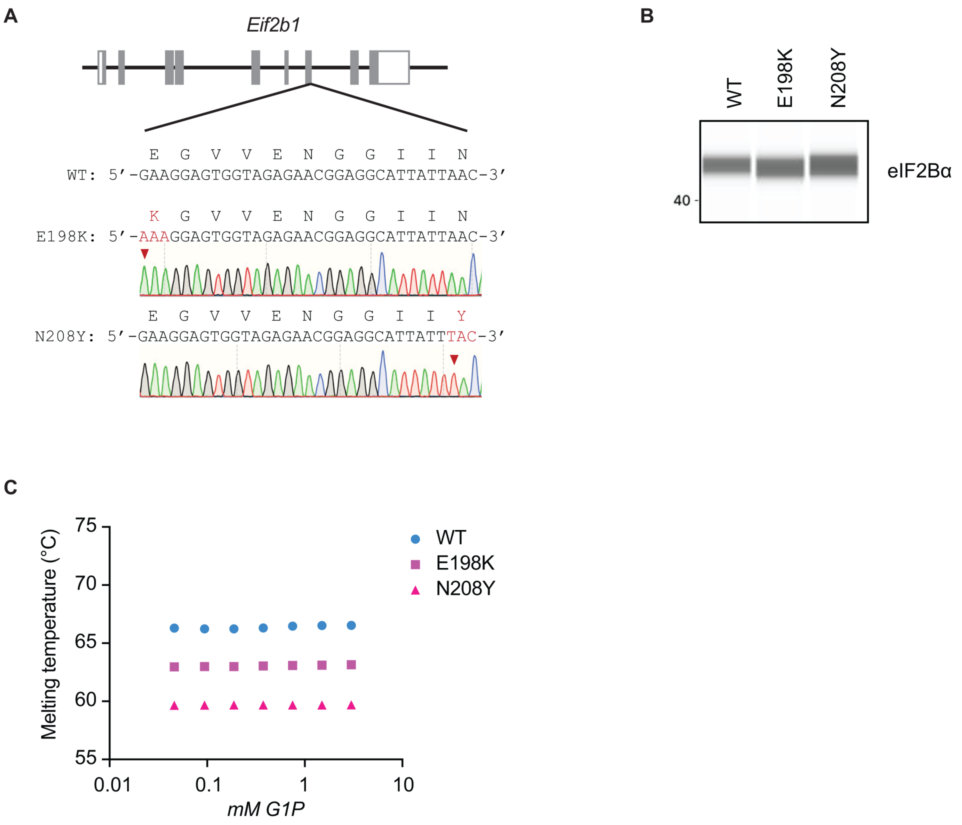

Figure Supplement 1

Figure Supplement 1.

(A) Schematic representation of mouse *Eif2b1* locus and nucleotide substitutions to generate E198K or N208Y mutated MIN-6 cell lines. Solid gray bars represent open reading frames, open bars represent UTRs. A representative sequencing result of a homozygous clone for each mutation is shown.

(B) Immunoblot of purified recombinant eIF2Bα WT, E198K or N208Y against the eIF2Bα antibody.

(C) Calculated midpoint thermal unfolding of recombinant human WT, E198K or N208Y eIF2Bα using NanoDSF with G1P. n=3. Error bars (too small to be displayed) are standard deviation.

A

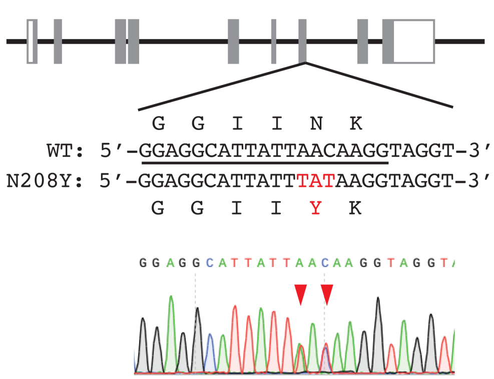

B

P0 Pups Viability

	WT	N208Y^{HET}	N208Y^{HOM}
Live	21 (100 %)	44 (95.7 %)	2 (11.1 %)
Dead	0 (0 %)	2 (4.3 %)	16 (88.9 %)

C

E18.5 Embryo Viability

Pinch response	WT	N208Y^{HET}	N208Y^{HOM}
Yes	13 (100 %)	30 (96.8 %)	13 (86.7 %)
No	0 (0 %)	1 (3.2 %)	2 (13.3 %)

D

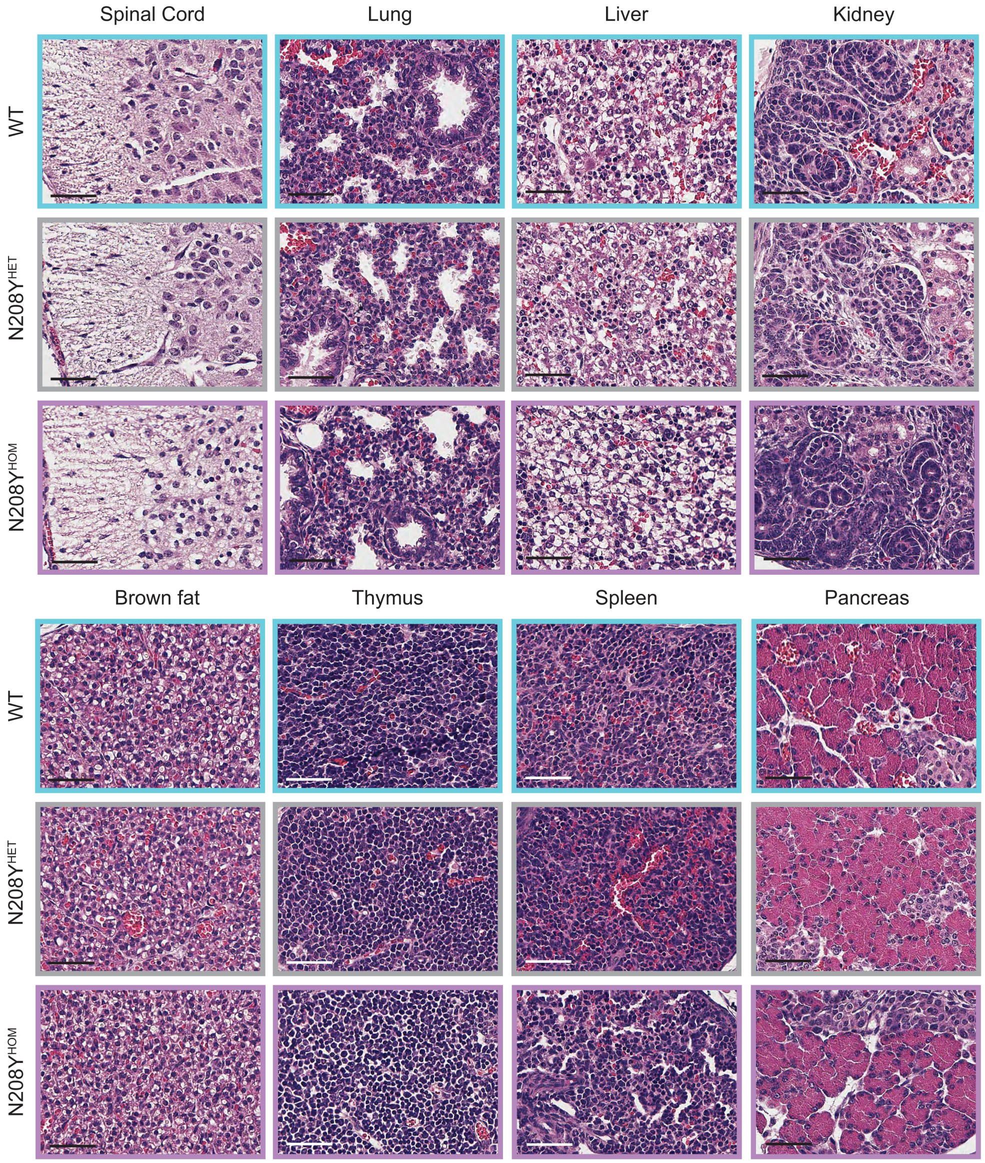

Figure Supplement 2

Figure Supplement 2.

(A) Schematic representation of mouse *Eif2b1* locus and nucleotide substitution to generate N208Y mutation. Solid gray bars represent open reading frames, Open bars represent UTRs. Underline indicates gRNA sequence used to target the locus. A representative sequencing result of the founder heterozygous mouse is shown. Red arrows indicate nucleotide substitution.

(B) Number of P0 pups found dead or alive in each genotype as shown in Figure 2B.

(C) Foot pinch responsiveness of E18.5 embryos for each genotype as shown in Figure 2D.

(D) Representative H & E images of spinal cord, lung, liver, kidney, brown fat, thymus, spleen, and pancreas from E18.5 embryos. Scale bars, 50 µm. Histopathological features of E18.5 embryos and P0 pups were evaluated by a veterinary pathologist. E18.5: WT, n = 4; $N208Y^{HET}$, n = 4, $N208Y^{HOM}$, n = 2; P0: WT, n = 4; $N208Y^{HET}$, n = 6, $N208Y^{HOM}$, n = 5.

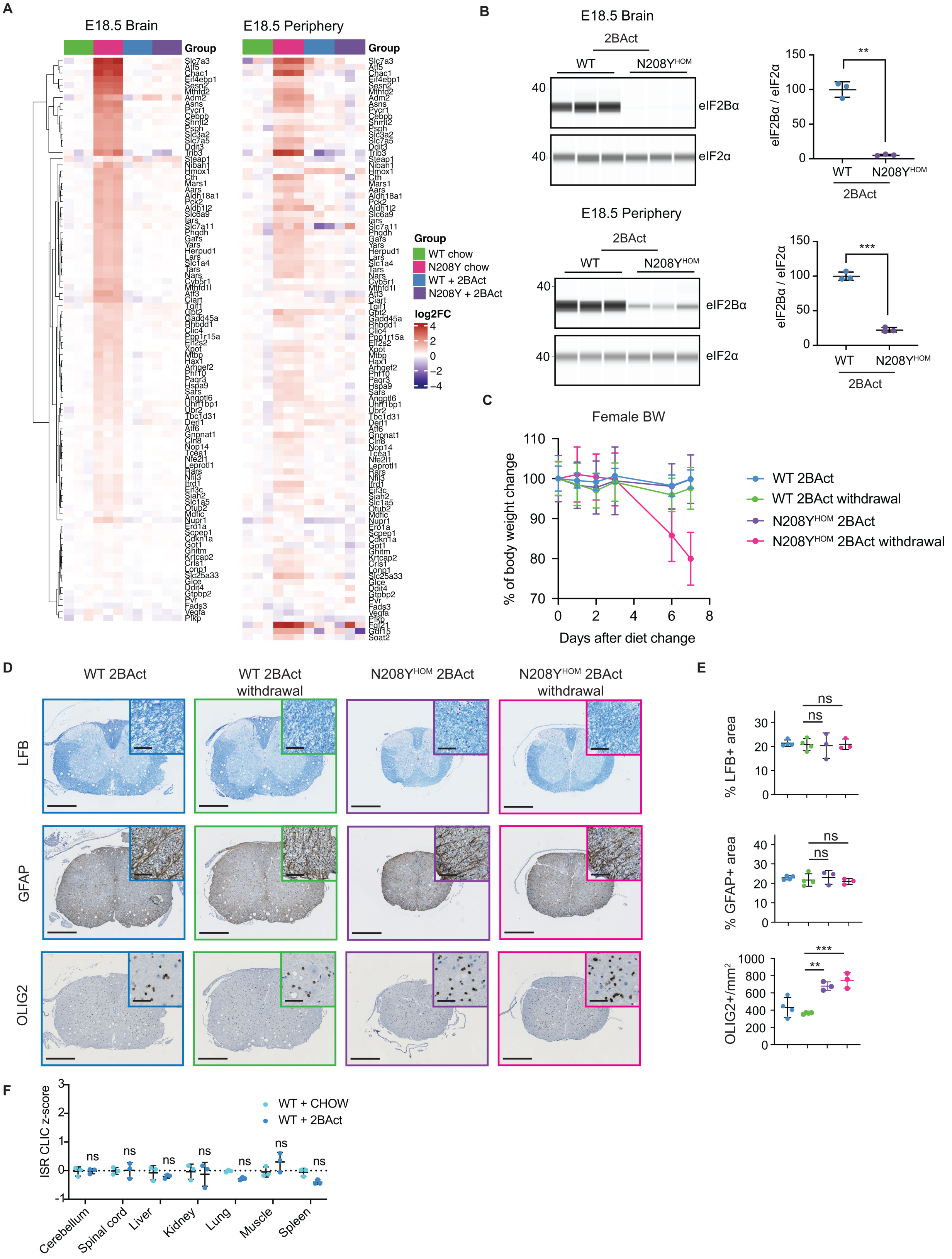

Figure Supplement 3

Figure Supplement 3.

(A) Heatmap of ISR CLIC genes from brain and pooled peripheral organs of E18.5 WT and N208Y^{HOM} embryos on chow diet or 2BAct. Heatmaps values are displayed as a $\log_2$ fold change versus the WT chow group.

(B) Immunoblot of eIF2Bα subunit in brain and pooled peripheral organ lysates from E18.5 WT and N208Y^{HOM} embryos with 2BAct. Graphs show quantification of eIF2Bα bands, normalized to eIF2α expression, and represented as % of WT expression. Error bars are standard deviation. Welch's t-test, ** $p< 0.01$, *** $p < 0.001$.

(C) Percentage of body weight change of 3-month-old female mice after 2BAct withdrawal. WT + 2BAct, n = 6; WT + 2BAct withdrawal, n = 7; N208Y^{HOM} + 2BAct, n = 3; N208Y^{HOM} + 2BAct withdrawal, n = 4

(D) Luxol fast blue and Immunohistochemical staining using antibodies specific to GFAP, and OLIG2 from spinal cords of 3-month-old females after 7-day 2BAct withdrawal. WT + 2BAct, n = 4; WT + 2BAct withdrawal, n = 4; N208Y^{HOM} + 2BAct, n = 3; N208Y^{HOM} + 2BAct withdrawal, n = 3. Scale bar 500 µm and 50 µm (inset)

(E) Quantification results of (D). One-way ANOVA with Holm-Sidak's multiple comparisons test. ** $p < 0.01$, *** $p < 0.001$, ns = not significant.

(F) Average z-score of the ISR CLIC genes calculated from nCounter gene expression profiling of various tissues from 4-month-old male mice WT + 2BAct-medicated diet normalized to WT + control det. WT + control diet, n = 3 and WT + 2BAct-medicated diet, n=3. Error bars are Standard deviation. Student's t-test; ns, not significant.

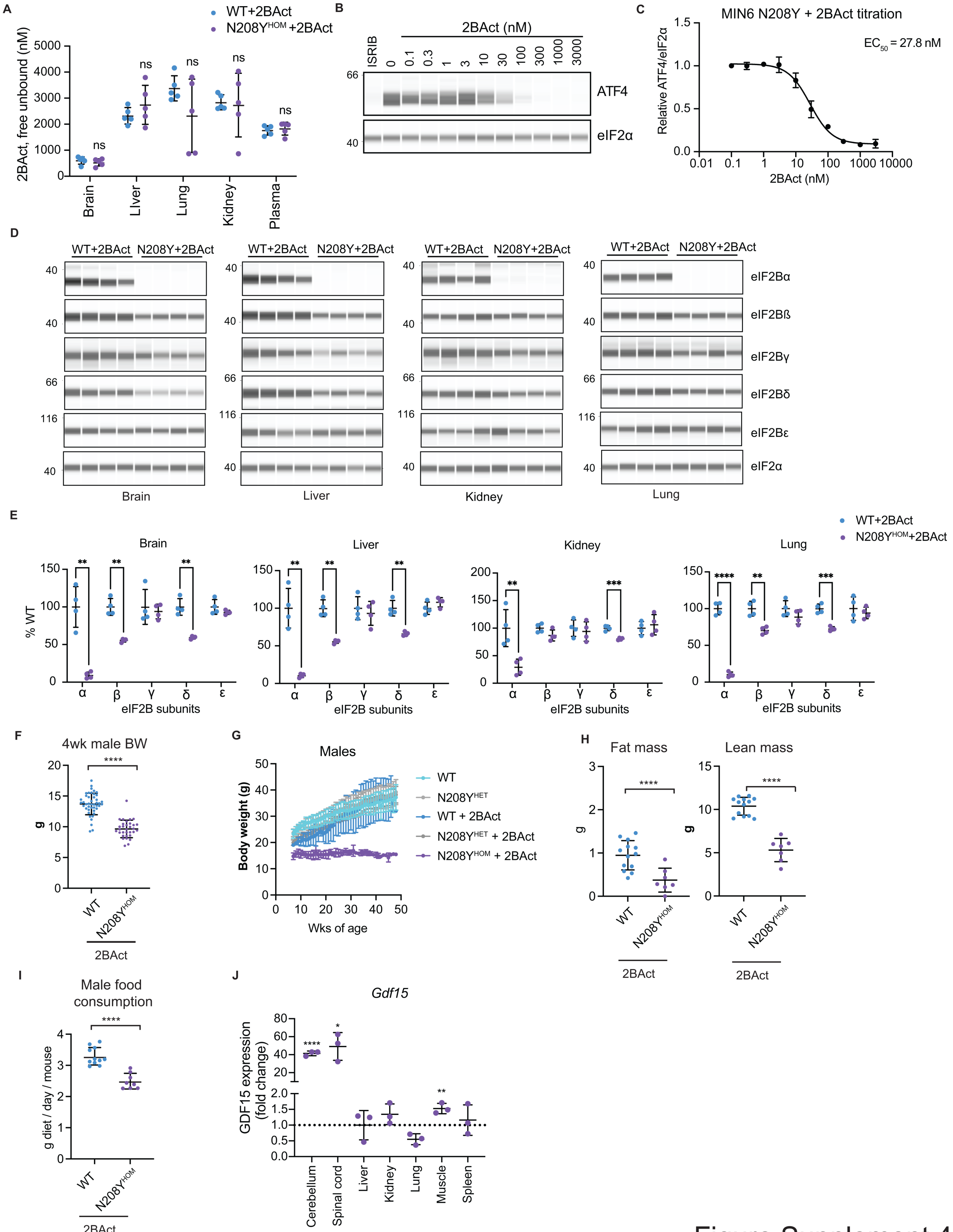

Figure Supplement 4

Figure Supplement 4.

(A) Tissue drug exposure of 3-month-old male mice. n=5. Error bars are Standard deviation. Two-way ANOVA with Holm-Sidak's multiple comparisons test. ns, not significant.

(B and C) 2BAct EC_{50} in MIN6 N208Y cell line was calculated from measuring ATF4 suppression from a 4 hr dose response of 2BAct following a 24 hr withdrawal of ISRIB. Immunoblot of ATF4 protein (B) was normalized to eIF2α (left) and displayed as relative abundance (C), n = 3.

(D) Immunoblot of eIF2B subunits in brain, liver, kidney, and lung lysates from 2BAct-treated WT and $N208Y^{HOM}$ mice.

(E) Quantification of bands in (D) normalized to eIF2α expression and represented as % of WT expression. n=4. Error bars are Standard deviation. Student's t-test, ** $p < 0.01$, *** $p < 0.001$, **** $p < 0.0001$.

(F) Body weight measurements of 4-week-old male WT and $N208Y^{HOM}$ mice with 2BAct. WT + 2BAct, n = 44; $N208Y^{HOM}$ + 2BAct, n = 32. Student's t-test. **** $p < 0.0001$

(G) Body weight measurements of WT, $N208Y^{HOM}$, and $N208Y^{HET}$ mice with 2BAct. Male WT, n = 6; $N208Y^{HET}$, n = 14, WT + 2BAct, n = 2, $N208Y^{HET}$ + 2BAct, n = 9; $N208Y^{HOM}$ + 2BAct, n = 4.

(H) Fat and lean mass measurement of 3-month-old female mice by EchoMRI. WT + control, n = 8; WT + 2BAct, n = 13; $N208Y^{HOM}$ + 2BAct, n = 7.

(I) Male daily food consumption measured between 5 and 6 weeks of age.WT + 2BAct, n = 44; $N208Y^{HOM}$ + 2BAct, n = 32.

(H) and (I) Error bars are Standard deviation. Student's t-test. **** $p < 0.0001$

(J) mRNA expression of *Gdf15* in various tissues normalized to WT + 2BAct using nCounter platform. n=4. Error bars are Standard deviation. Student's t-test, * $p< 0.05$, ** $p < 0.01$, **** $p < 0.0001$.

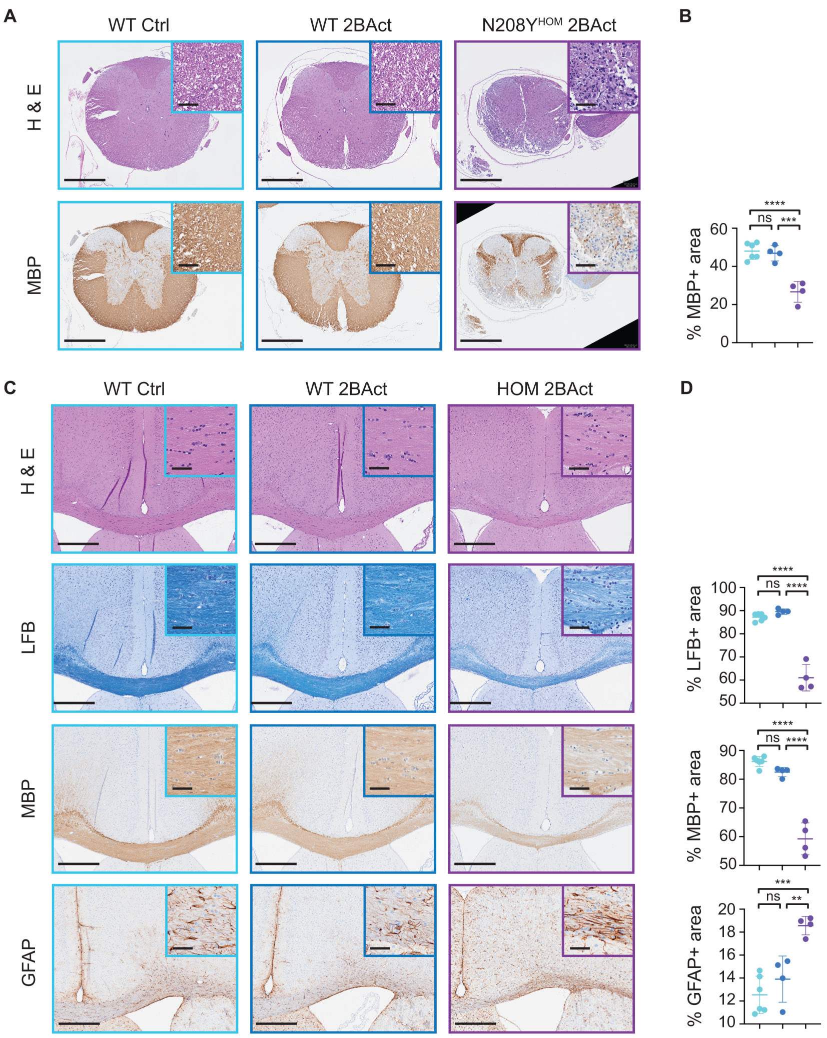

Figure Supplement 5

Figure Supplement 5.

(A and B) Representative H & E and Myelin Basic Protein (MBP) IHC images (A) and quantification (B) of the thoracic region of the spinal cord.

(C and D) Representative H & E, Luxol Fast Blue (LFB) staining, Myelin Basic Protein (MBP), and GFAP IHC images (C) and quantification (D) of the corpus callosum region of the brain.

(A to D) Scale bars, 500 µm. Inset scale bars, 50 µm. WT+Ctrl, n = 6 (3 females and 3 males); WT+2BAct, n = 4 (2 females and 2 males); N208Y^{HOM} + 2BAct, n = 4 (2 females and 2 males). Error bars are Standard deviation. One-way ANOVA with Holm-Sidak's multiple comparisons test. * $p < 0.05$, ** $p < 0.01$, *** $p < 0.001$, **** $p < 0.0001$, ns = not significant.

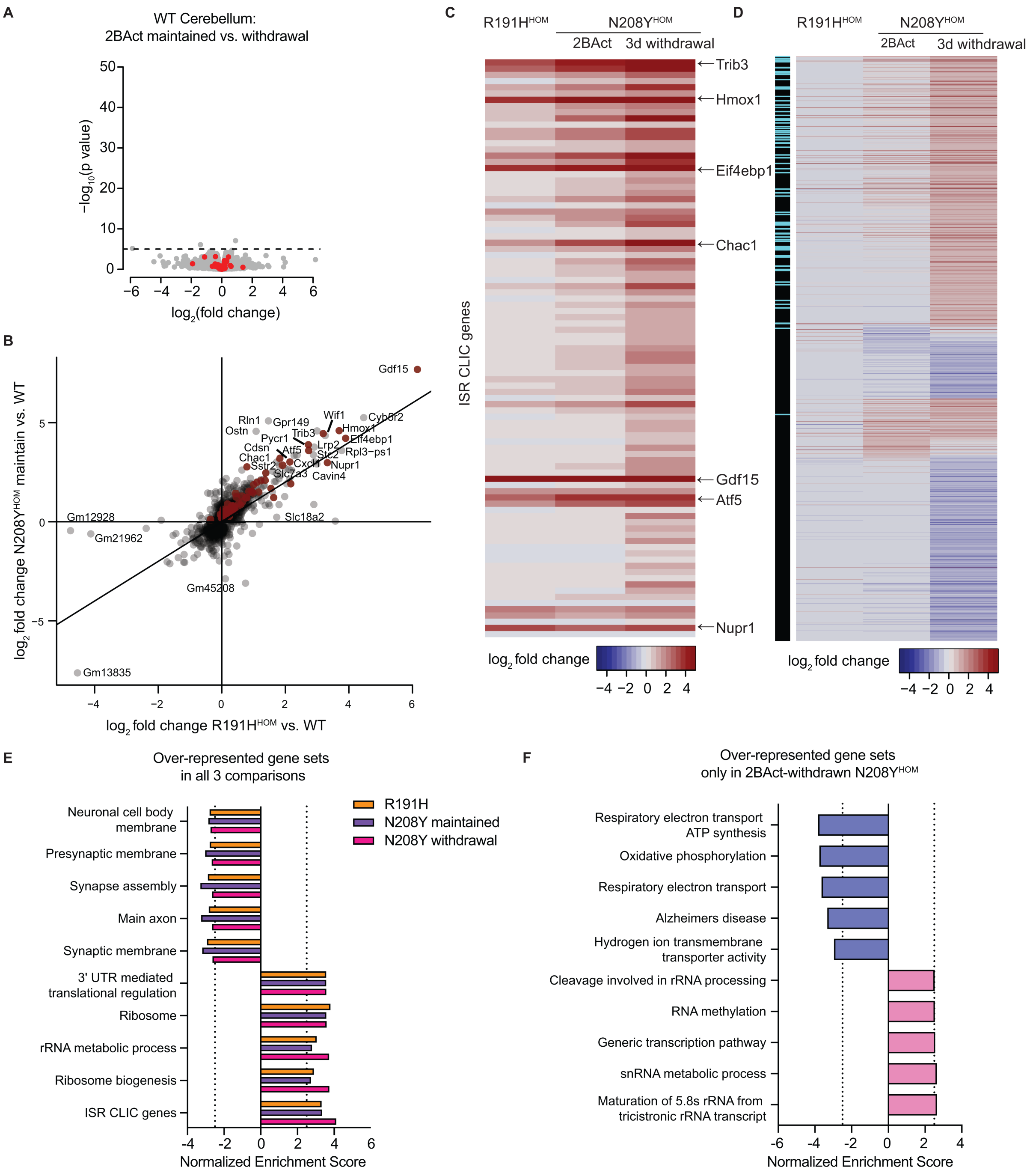

Figure Supplement 6

Figure Supplement 6.

(A) Volcano plot demonstrating gene expression changes between 2BAct maintained and withdrawal in WT cerebellum. Red dots show ISR CLIC genes and the dotted line indicates significance threshold (adj p < 0.05).

(B) Correlation plot showing differentially expressed genes from either R191H^{HOM} or N208Y^{HOM} + 2BAct compared to their respective wild type groups. The genes with more than 3 $\log_2$ fold change were labeled. Dark red dots indicate significantly upregulated ISR CLIC genes (adj p < 0.05).

(C and D) Heatmap of (C) ISR CLIC genes and (D) all significantly differentially expressed genes from R191H^{HOM} vs WT, N208Y^{HOM} + 2BAct vs WT + 2BAct, and N208Y^{HOM} 2BAct withdrawal vs WT + 2BAct comparisons. In (D), on the left, ISR CLIC genes are shown in light blue.

(E and F) The top 5 most significantly overrepresented gene sets by fgsea in all three comparisons (E) or only in N208Y^{HOM} 2BAct withdrawal vs WT + 2BAct comparison (F). Cutoff of Normalized Enrichment Score (2.5) is presented as dotted lines.

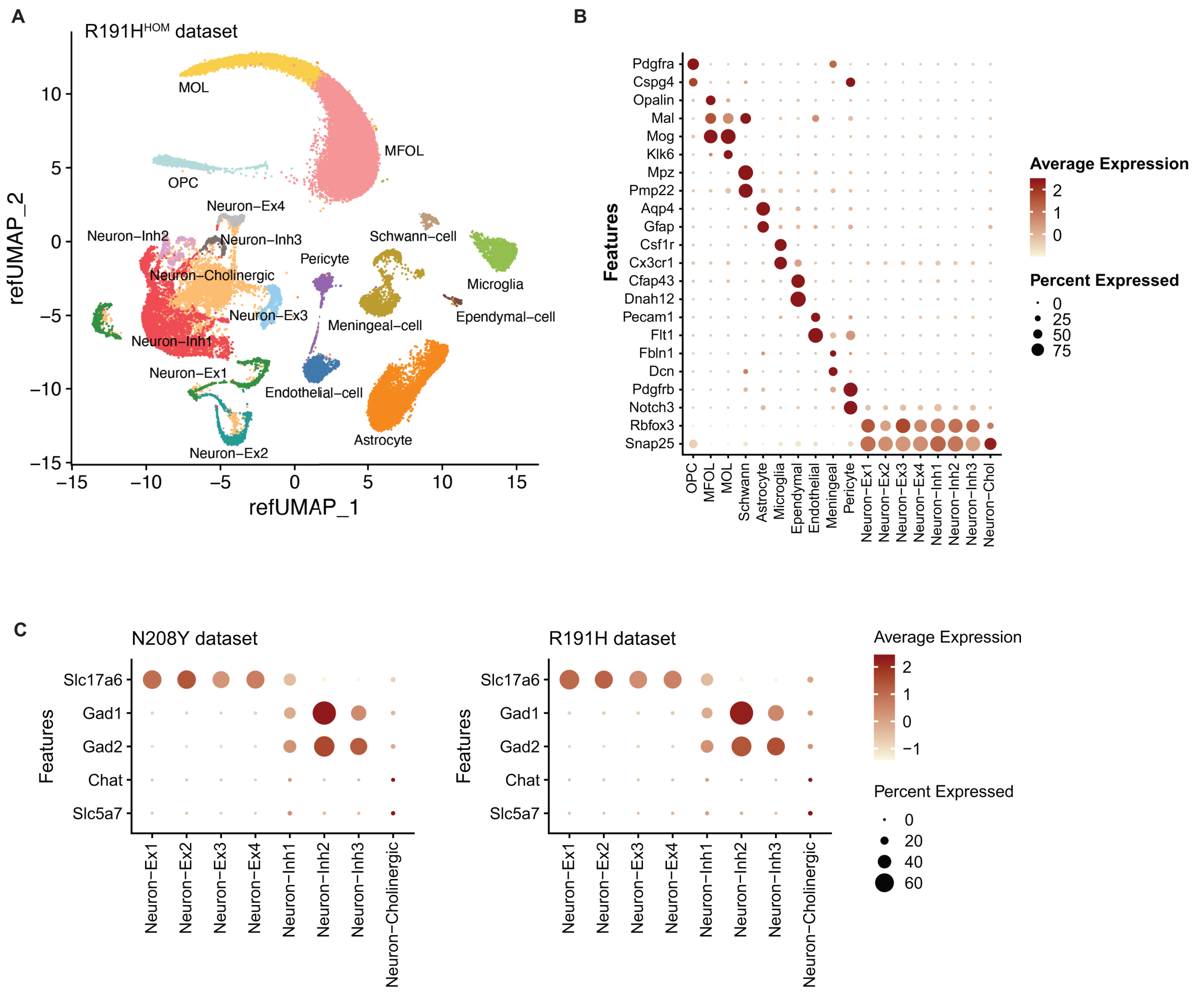

Figure Supplement 7

Figure Supplement 7.

(A) Projection of 50,731 single nuclei isolated from cervical thoracic spinal cord of untreated female, 2.5-month-old WT and R191H^{HOM} mice (n=3 per group) onto the N208Y dataset UMAP structure. Cell type labels were transferred using the N208Y dataset as a reference. OPC, oligodendrocyte progenitor cell; MFOL, myelin-forming oligodendrocytes; MOL, mature oligodendrocytes.

(B) Dot plot expression of marker genes for major cell types in the spinal cord from the R191H dataset.

(C) Dot plot expression of neurotransmitter genes for classifying spinal cord neurons in R191H and N208Y mouse model datasets.

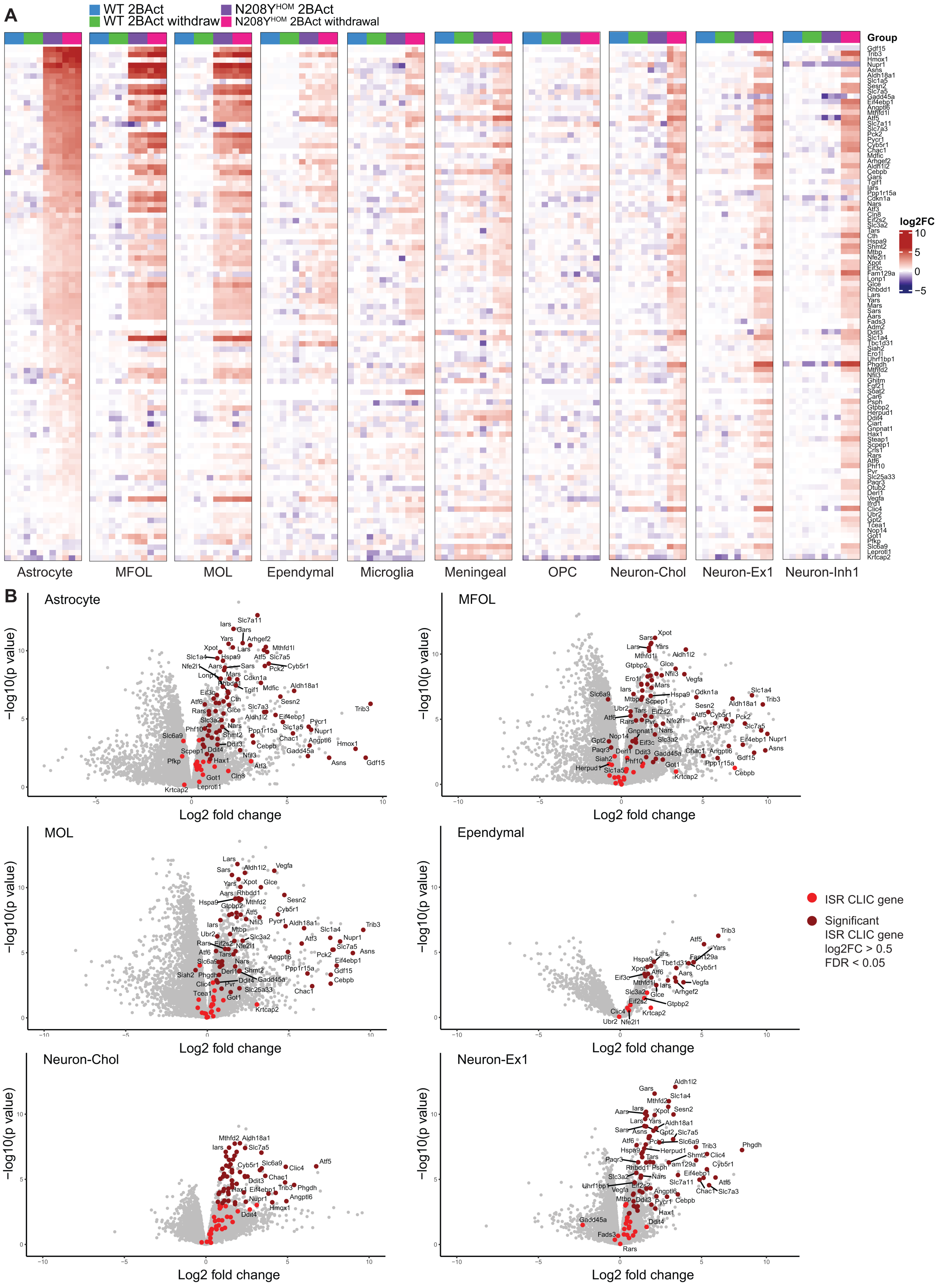

Figure Supplement 8

Figure Supplement 8.

(A) Pseudobulk expression profiles of all ISR CLIC genes in select cell types from spinal cord of WT and N208Y^{HOM} mice maintained on 2BAct or 3 days after 2BAct withdrawal (n=3 per group). Heatmaps values are displayed as a $\log_2$ fold change versus the WT 2BAct-maintained group.

(B) Volcano plots of select cell types displaying differences in gene expression between N208Y^{HOM} 2BAct withdrawal versus WT 2BAct-maintained groups. Red dots show ISR CLIC genes and dark red indicate significantly up- or down-regulated ISR CLIC genes (adj $p < 0.05$, log2FC > 0.5).

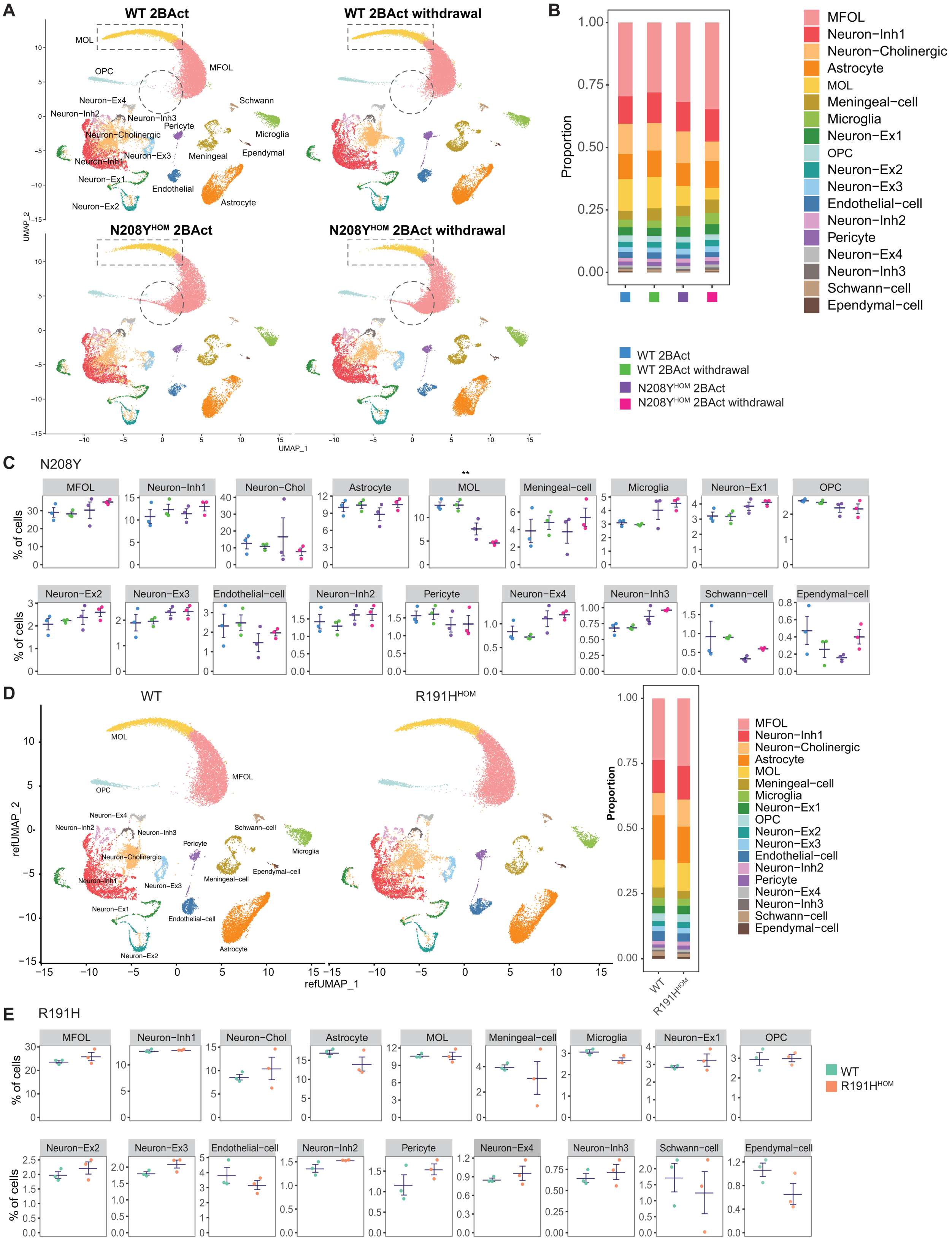

Figure Supplement 9

Figure Supplement 9.

(A) UMAP plots of all spinal cord nuclei from WT and N208Y^{HOM} mice maintained on 2BAct or 3 days after 2BAct withdrawal, split by genotype and treatment (n=3 per group). Differences between genotypes in the myelin-forming oligodendrocyte (MFOL) populations and mature oligodendrocyte (MOL) populations are highlighted with a dotted circle and dotted rectangle, respectively. OPC, oligodendrocyte progenitor cell.

(B) Summary of cell type composition, split by genotype and treatment groups (n=3 per group). Cell types are ordered from most abundant to least abundant.

(C) Proportions of all cell clusters from WT and N208Y^{HOM} mice maintained on 2BAct or 3 days after 2BAct withdrawal. One-way ANOVA. ** $p < 0.01$.

(D) UMAP plots (left) and cell type composition summary (right) of all spinal cord nuclei from untreated WT and R191H^{HOM} mice, split by genotype (n=3 per group).

(E) Proportions of all cell clusters from untreated WT and R191H^{HOM} mice

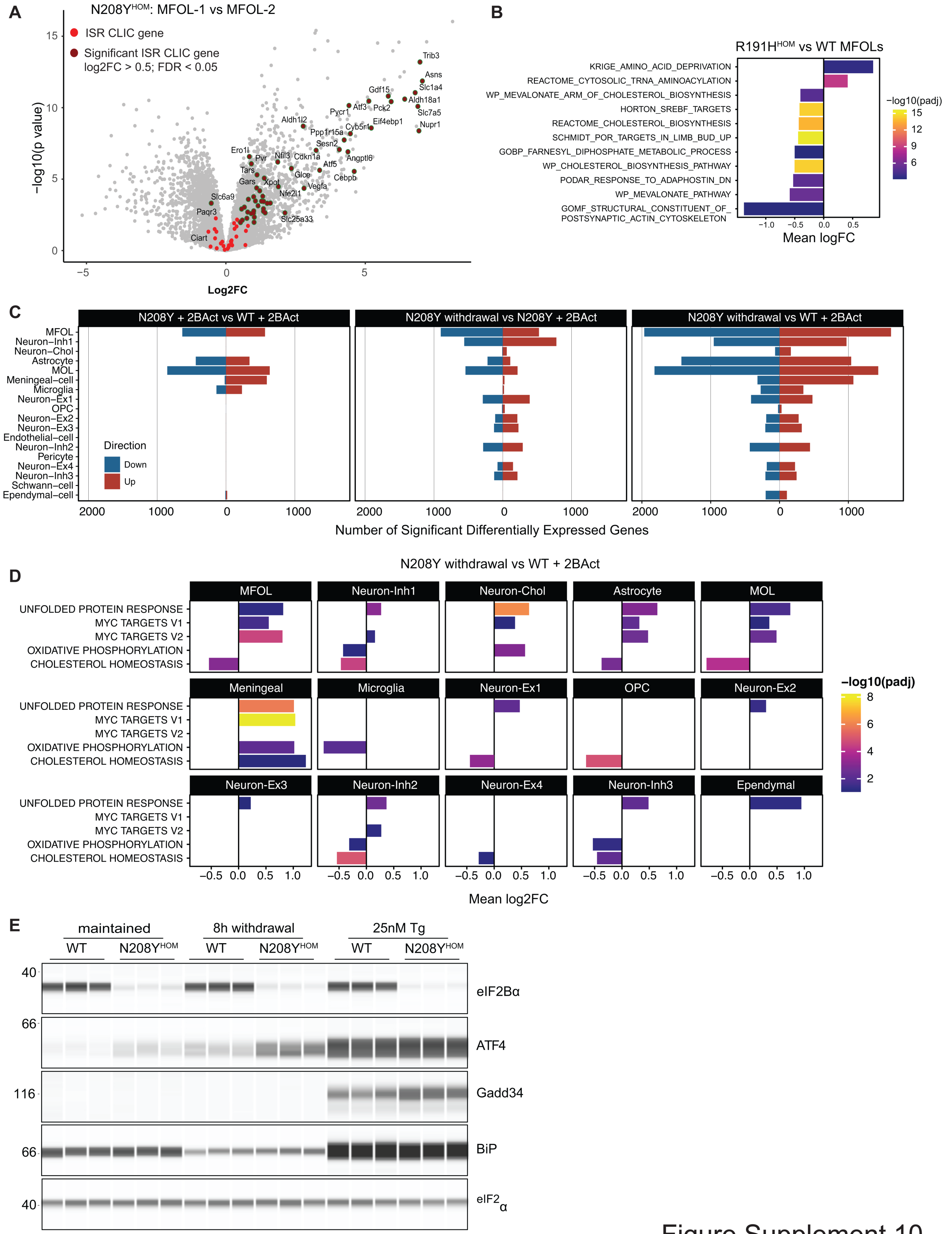

Figure Supplement 10

Figure Supplement 10.

(A) Volcano plots of differences in gene expression between MFOL-1 and MFOL-2 clusters from the N208Y dataset. Red dots show ISR CLIC genes and dark red indicate significantly up- or down-regulated ISR CLIC genes (adj $p < 0.05$, log2FC > 0.5).

(B) Gene set enrichment analysis of MFOLs from untreated R191H^{HOM} compared to WT.

(C) Significant differentially expressed genes (adj $p < 0.05$, log2FC > 0.5) in each cell type across three comparisons: N208Y^{HOM} + 2BAct vs WT + 2BAct, N208Y^{HOM} 2BAct withdrawal vs N208Y^{HOM} + 2BAct, and N208Y^{HOM} 2BAct withdrawal vs WT + 2BAct.

(D) Top Hallmark pathways that change in each cell type in the N208Y^{HOM} 2BAct withdrawal vs WT + 2BAct comparison.

(E) Immunoblot analysis of eIF2Bα, ATF4, BiP, Gadd34 and eIF2α of WT or N208Y^{HOM} MEFs maintained on 2BAct or after 8-hour 2BAct withdrawal compared to 8-hour 25nM Tg treatment.

Supplementary Tables (excel files attached separately, titles listed below)

Table S1. Fold-change of ISR CLIC gene expression panel for N208Y^{HOM} vs WT in E18.5 brain and pooled peripheral organs. n=3. Two-way ANOVA with Holm-Sidak's multiple comparison test. * p < 0.05, ** p < 0.01, *** p < 0.001, **** p < 0.0001

Table S2. Fold-change of ISR CLIC gene expression panel in cerebellum, spinal cord, kidney, lung, muscle, liver, and spleen from 2BAct-withdrawn N208Y^{HOM} animals compared to 2BAct-maintained WT animals. n=4. Two-way ANOVA with Holm-Sidak's multiple comparison test. * p < 0.05, ** p < 0.01, *** p < 0.001, **** p < 0.0001

Table S3. List of differentially expressed genes in the cerebellum of R191H^{HOM} vs WT, N208Y^{HOM} + 2BAct vs WT + 2BAct, and N208Y^{HOM} 2BAct withdrawal vs WT + 2BAct comparisons, as shown in Figure Supplement 6B and 6D.

Table S4. List of ISR CLIC genes differentially expressed in cerebellum of R191H^{HOM} vs WT, N208Y^{HOM} + 2BAct vs WT + 2BAct, and N208Y^{HOM} 2BAct withdrawal vs WT + 2BAct comparisons, as shown in Supplement Figure 6C.

Table S5. List of gene sets significantly over-represented in cerebellum of all three comparisons as shown in Figure Supplement 6E or only in N208Y^{HOM} 2BAct withdrawal vs WT + 2BAct comparison as shown in Figure Supplement 6F. The gene sets with values of Normalized Enrichment Score (NES) > 2.5 and adjusted p < 0.05 were shown.

Table S6. Differential gene expression analysis results from pseudobulked MFOL-1 versus MFOL-2 clusters (log_2 fold change > 0.5 and FDR < 0.05).

Table S7. Gene set enrichment analysis results of all comparisons (N208Y^{HOM} + 2BAct vs WT + 2BAct, N208Y^{HOM} 2BAct withdrawal vs N208Y^{HOM} + 2BAct, and N208Y^{HOM} 2BAct withdrawal vs WT + 2BAct) across all cell types.

Table S8. Differential expression testing of ATF6 and IRE1 UPR genes and cholesterol biosynthesis genes in bulk spinal cord and cerebellum of N208Y^{HOM} + 2BAct vs WT + 2BAct and N208Y^{HOM} 2BAct withdrawal vs N208Y + 2BAct comparisons.

Table S9. Fold-change of ISR CLIC and cholesterol biosynthesis gene expression in 2BAct-maintained or withdrawn mouse embryonic fibroblasts (MEFs) of WT and N208Y^{HOM} genotypes compared to 2BAct-maintained WT MEFs. n = 2-3 biological replicates.

Supplementary Key Resources Table

Reagent type or resource	Designation	Source or reference	Identifiers	Additional information
crRNA	CTTGTCATAGTTGGTGCTGA	IDT		eIF2Bα E198K
crRNA	GGAGGCATTATTAACAAGGT	IDT		eIF2Bα N208Y
HDR donor sequence	GCTATATCATGGAGAAAGCAGATCTTGTCATAGTTGGTGCCAAAGGAGTGGTAGAGAACGGAGGCATTATTAACAAGGTAGGTA	IDT		E198K (GAA>AAA) mutation and silent gRNA mutation
HDR donor sequence	AGTTGGTGCTGAAGGAGTGGTAGAGAACGGAGGCATTATTTACAAGGTAGCTACTGTCATACTGTTCCCAGAAGCCCCTACAGCC	IDT		N208Y (AAC>TAC) mutation and silent gRNA mutation
Cell line (*M. musculus*)	MIN6 WT clones	This paper		2 clones generated
Cell line (*M. musculus*)	MIN6 E198K clones	This paper		2 clones generated
Cell line (*M. musculus*)	MIN6 N208Y clones	This paper		2 clones generated

Antibody	Rabbit polyclonal anti-MBP	Abcam	#ab40390	IHC 5 ug/ml; epitope retrieval with pepsin pH 2.3, 10–20 min
Antibody	Rabbit monoclonal anti-GFAP	Cell Signaling	#12389	IHC 0.1 ug/ml; BOND Epitope Retrieval Solution 2 (Leica), 100 °C, 20 min<br>Used for 11 MO mice
Antibody	Rabbit monoclonal anti-GFAP	Abcam	#ab68428	IHC 0.05 ug/ml; Epitope retrieval with pH6, 95C 20 min<br>Used for 3 MO mice +/- 2BAct
Antibody	Rabbit polyclonal anti-IBA1	Wako Chemicals	#019–19741	IHC 1 ug/ml; BOND Epitope Retrieval Solution 2 (Leica), 100 °C, 20 min
Antibody	Rabbit monoclonal anti-ATF3	Abcam	#ab207434	IHC 4 ug/ml; BOND Epitope Retrieval Solution 2 (Leica), 100 °C, 20 min

Antibody	Rabbit monoclonal anti-OLIG2	Abcam	#ab109186	IHC 0.3 ug/ml; BOND Epitope Retrieval Solution 1 (Leica), 100 °C, 20 min<br>Used for 4 and 11 MO mice<br><br>IHC 0.7 ug/ml; epitope retrieval with EDTA pH 9, 95C, 20 min<br>Used for 3 MO mice +/- 2BAct
Antibody	Goat polyclonal anti-PDGFRα	R&D Systems	AF1062	IHC 0.4 ug/ml; BOND Epitope Retrieval Solution 1 (Leica), 100 °C, 20 min
Antibody	Mouse monoclonal anti-CC-1	Sigma-Aldrich	OP80	IHC 0.08 ug/ml; BOND Epitope Retrieval Solution 1 (Leica), 100 °C, 20 min
Antibody	Goal polyclonal anti-TPPP	Invitrogen	PA5-19243	IHC 0.22 ug/ml; BOND Epitope Retrieval Solution 1 (Leica), 100 °C, 20

				min
Other	Luxol Fast Blue	Electron Microscopy Sciences	#26681	Histological stain for myelin of 11 MO mice.
Other	Luxol Fast Blue	StatLab	#STLFBPT	Histological stain for myelin of 3 MO mice +/- 2BAct
Antibody	Rabbit monoclonal anti-ATF4	Cell Signaling	#11815	1:50 in Wes
Antibody	Rabbit monoclonal anti-eIF2α	Cell Signaling	#5324	1:500 in Wes
Antibody	Rabbit polyclonal anti-eIF2Bα	Novus	#NBP2-16292	1:50 in Wes
Antibody	Rabbit polyclonal anti-eIF2Bβ	Novus	#NBP2-38572	1:50 in Wes
Antibody	Rabbit polyclonal anti-eIF2Bγ	Proteintech	#11296-2-AP	1:50 in Wes
Antibody	Rabbit polyclonal anti-eIF2Bδ	Proteintech	#11332-1-AP	1:50 in Wes
Antibody	Rabbit polyclonal anti-eIF2Bε	Novus	#NBP1-32783	1:25 in Wes
Compound	Fructose-6-phosphate	Sigma	#F3627-1G	Resuspended in water

Compound	Glucose-1-phosphate	Sigma	#G7018-1G	Resuspended in water
Chemical compound	ISRIB	(1)		
Chemical compound	2BAct	(1)		
Chemical compound	2BAct medicated diet	(1)		
Gene expression	*Atf3*	Applied Biosystems	Mm00476032_m1	
Gene expression	*Chop*	Applied Biosystems	Mm01135937_g1	
Gene expression	*Actin*	Applied Biosystems	Mm00607939_s1	
Gene expression	ISR CLIC genes	Nanostring	Custom	95 genes (1) + *Fgf21*
Cholesterol standard	Cholesterol-d7	Avanti	700041P-25mg	0.9 ppm

Supplementary Methods

Generation of mutant cell lines and cell culture

The eIF2Bα E198K and N208Y mutations were introduced into MIN-6 cells (AddexBio #C0018008) using the Alt-R CRISPR-Cas9 System (Integrated DNA Technologies, Coralville, IA, USA) following manufacturer's protocol. The RNP and HDR donor were delivered to MIN-6 cells by electroporation using the Lonza 4D-Nucleofector (Lonza Group AG, Basel, Switzerland), using the 96-well SE reagent kit and program CM-150. Nucleofected cells were incubated in media containing Alt-R HDR Enhancer and 200 nM ISRIB for 24 hr before media was changed to complete media + ISRIB. After 48 hr, cells were single-sorted into 96-well plates containing complete media + ISRIB using a BD Biosciences FACSAria Fusion (BD Biosciences, San Jose, CA, USA). Clones were expanded and genomic DNA was sequenced to confirm the desired mutations. Experiments involving ISRIB withdrawal from cell lines were accomplished by one round of PBS wash followed by addition of complete media without ISRIB.

MIN-6 cell lines were grown in complete media (DMEM containing 25 mM glucose supplemented with 15% FBS, 100 U/mL streptomycin, 100 U/mL penicillin sulfate and 75 uM β-mercaptoethanol). Cell lines were maintained in complete media + 200 nM ISRIB. Cell lines were incubated at 37°C, 5% CO_2.

Immunoblots

Cell lysates were prepared in RIPA buffer (Thermo Scientific #89900) with protease/phosphatase inhibitors (Thermo Scientific #78444). Cells were lysed on ice for 10 min then centrifuged (21,000 x g, 15 min, 4C) to remove cellular debris. Tissue lysates were homogenized by mortar pestle grinding in liquid nitrogen prior to bead-based homogenization in RIPA buffer. All lysates were incubated on ice for 10 min then centrifuge (21,000 x g, 15 min, 4C) to remove cellular debris. Lysate concentration was determined using BCA and cell protein lysate samples were adjusted to 0.5 mg/mL and tissue protein lysate samples were adjusted to 0.75 mg/mL. Samples were run on a ProteinSimple Wes system (Bio-Techne, Minneapolis, MN, USA) using a 12-230 kDa separation module. GraphPad Prism (La Jolla, CA, USA) was used to perform statistical analyses utilizing two-way ANOVA with Holm-sidak performed post-hoc to correct for multiple comparisons.

Differential scanning fluorimetry

Recombinant eIF2Bα WT, E198K, N208Y were purified as previously described (2). Thermal shift assay was performed on Prometheus Panta NT.48 from NanoTemper technologies by measuring the intrinsic dual-UV fluorescence change in tryptophan and tyrosine residues in proteins at emission wavelengths of k = 330 and 350 nm.The ratio of the recorded emission intensities (Em350nm/Em330nm), which represents the change in TRP fluorescence intensity as well as the shift of the emission maximum to higher wavelengths ("red-shift") or lower wavelengths ("blue-shift") was plotted as a function of the temperature. The fluorescence intensity ratio and its first derivative were calculated and determined to be the melting temperature (Tm), with the manufacturer's software (PR.Panta Control and PR.Panta Analysis). The samples were loaded using capillaries in a volume of 10μL on Prometheus Panta NT.48 from NanoTemper Technologies. 50 μM eIF2Bα was incubated with 2-fold serial dilution of the indicated sugar phosphates at a concentration titration of 0.046mM - 3mM, and then subjected to thermal change from 25°C - 85°C with a ramp rate of 1°C/min.

Generation of *Eif2b1* (p.N208Y) mutant mouse model.

The mutant mouse strain carrying the *Eif2b1* p.N208Y point mutation was generated as a service by Cyagen Inc. through targeted mutagenesis in mouse embryos using the CRISPR/Cas9 system. In brief, the Cas9 protein, the synthesized sgRNA (5'-GGAGGCATTATTAACAAGGTAGG-3') and ssDNA donor (5'-
GAGAAAGCAGATCTTGTCATAGTTGGTGCTGAAGGAGTGGTAGAGAACGGAGGCAT
TATTTATAAGGTAGGTACTGTCATACTGTTCCCAGAAGCCCCTACAGCCTGAGCAAG
ACCTTGTCAG-3') harboring the p.N208Y mutation (AAC to TAT) were co-injected into the cytoplasm of pronuclear stage embryos. After overnight incubation, the two-cell stage embryos were selected and transferred into the oviduct of pseudopregnant ICR females. Pups were genotyped by PCR with two primers (5'-CTCACTATTGAGGTGGTTAGAGGT-3', 5'-AAACCAGAGACACTCAATTCCAAG-3') followed by Sanger sequencing to confirm nucleotide substitutions. The identified mutant founders were further

back-crossed with wild-type C57BL/6N mice to generate *Eif2b1*$^{N208Y/+}$ (N208Y^{HET}) mutant mice. Genotyping was performed by Transnetyx using real-time PCR.

Animal breeding and study

To assess homozygous phenotype, N208Y^{HET} male and female mice were bred, and the pups were monitored daily at Taconic Biosciences, Inc. as a service and Calico Life Sciences, LLC. or. All dead pups were collected and genotyped, along with the weaned mice, to assess genotype-specific lethality. To study the drug effect, 30 mg/kg or 100 mg/kg 2BAct-medicated diet was prepared as previously described (1) and provided to N208Y^{HET} breeding pairs upon breeding setup and continued during the nursing period. The same diet without added compound was used as the control diet. Offspring were genotyped, weaned with 30 mg/kg 2BAct-medicated or control diet, and their body weight was monitored and recorded weekly. To study the phenotype of E18.5 embryos, N208Y^{HET} males and females were fed with a control or 30 mg/kg 2BAct-medicated diet a week prior to the mating and the vaginal plug was checked daily after mating. The day the vaginal plug was detected was considered as embryonic day 0.5 (E0.5). Pregnant female mice were euthanized at E18.5 by CO_2 and a caesarean section was performed to obtain the embryos. The E18.5 embryos were placed on a warm pad, imaged, and monitored for up to 15 minutes while breathing, movement, and foot pinch response were assessed as described (3), followed by weight measurement before euthanasia for histopathology, RNA extraction, or western blot analysis. Lean and fat mass of individual mice were measured using EchoMRI-100 (EchoMRI, Houston, TX). To score motor deficit, individual mouse was placed on an open field and monitored the presence of abnormal gait, tremor, and other neurological features, then scored its disease severity according to the published clinical scoring system (4). All animal experiments and methods were approved by the Institutional Animal Care and Use Committee of Calico Life Sciences, LLC. and Taconic Biosciences, Inc.

ELISA

3–4-month-old mice were euthanized with CO_2 and whole blood was collected via cardiac puncture and placed in K2 EDTA-treated BD microtainer tubes. Plasma was collected after spinning at 7000 g for 4 min

at 4 °C and plasma GDF15 and FGF21 levels were quantified using the GDF15 ELISA (R&D #MGD150) and FGF21 ELISA (BioVender #RD291108200R), respectively, following manufacturer's instructions. Plasma 2BAct drug exposure was measured as previously described (1).

Embryo or tissue collection

For RNA extraction and western blot analysis, 3–4-month-old mice were euthanized with CO_2, and cerebellum, spinal cord, liver, kidney, lung, muscle and spleen were freshly collected and snap frozen in liquid nitrogen and stored at -80 °C. For histology and immunohistochemistry, mice were deeply anesthetized and perfused with normal saline followed by 10% buffered formalin via transcardiac perfusion. Brains and spines were excised and post-fixed in 10% buffered formalin for 24-48 hours. For E18.5 embryo histopathology, embryos were fixed with 10% buffered formalin for 24 hours. For RNA extraction and western blot analysis, E18.5 embryos were euthanized by decapitation and placed on ice. Brain was collected from the head and snap-frozen in liquid nitrogen. For peripheral organs, all internal organs from body cavity, including trachea, esophagus, lung, heart, thymus, liver, pancreas, spleen, stomach, bladder, kidney, adrenal gland, and intestine, were collected, pooled in one tube per animal, then snap frozen in liquid nitrogen. The embryo organs were then stored at -80 °C.

Histology, histopathology, and immunohistochemistry

After fixation, E18.5 embryos were sliced along the transverse plane. Brains were coronally sliced and spinal cords were collected via laminectomy on the spinal column and separated into cervical, thoracic & lumbar regions, and processed using an automated tissue processor (Sakura Tissue-Tek VIP6 AI) following a standard dehydration and clearing protocol. The samples underwent graded ethanol dehydration (70%, 80%, 95%, and 100% ethanol, 1 hour per step), followed by 2 hours of clearing in xylene. Next, tissues were infiltrated with molten paraffin wax (Surgipath Paraplast, Leica) at 60°C for 3 hours. After infiltration, tissues were embedded into paraffin blocks using a tissue embedding station (HistoCore Arcadia H, Leica), sectioned at a thickness of 5 - 6 µm using a rotary microtome (HistoCore AUTOCUT, Leica), and mounted on adhesive-coated slides for staining. Histopathological evaluation of E18.5 embryos was carried out by

Comparative Pathology Laboratory, University of California Davis School of Veterinary Medicine (Davis, CA, USA) as a service. Immunohistochemistry was carried out in-house using Leica Bond RX Automated IHC Platform using modified IHC protocol F or by Histobridge, LLC. using Dako Link48 plus as a service with the primary antibodies described in the Key Resources Table and species-appropriate ImmPRESS Polymer Detection Kit (Vector Laboratories) and developed with 3,3'-Diaminobenzidine (DAB) or BOND IHC Polymer Refine Detection (DS9800, Leica Biosystems) followed by hematoxylin counterstaining. Luxol Fast Blue (LFB) was carried out following the manufacturer's instructions. After staining, sections were dehydrated through successive ethanol solutions, cleared in xylene, and coverslipped using xylene-based mounting media. To examine histopathological timepoints in the brain, sections from two coronal levels of the corpus callosum were examined. For the spinal cord, coronal sections from cervical and thoracic levels were examined. Image capture was achieved using Olympus VS200 slide scanner (Olympus), NanoZoomer HT2.0, or Zeiss Axioscan7. Image analysis was performed using HALO image analysis software (Indica Labs), VS200 Desktop (Olympus), or a QuPath (5). The same parameters for microscopy and image analysis were uniformly applied to all images for each timepoint and histological staining. For the spinal cord and LFB and MBP staining of corpus callosum, the mean area fraction from two sections from both the cervical and thoracic levels served as the value for each subject to normalize to the different size of the anatomical levels. For other staining of the corpus callosum, positive staining from a fixed area in the corpus callosum of a single section at each coronal level served as the value for each subject.

Bioanalysis of 2BAct

Plasma, liver, lung, kidney, and brain samples were sent to Quantitative, Translational & ADME Sciences group at Abbvie Lake County for bioanalysis. Samples and standards were extracted by protein precipitation and analytes were separated using C18 reverse phase chromatography prior to analysis with tandem mass spectrometry. Sample concentrations were calculated using the equation derived from regression analysis of the peak area ratio (analyte/internal standard) of the spiked standards versus concentrations.

GEF assay

WT and MIN-6 N208Y cells were grown to approximately 80% confluence in complete culture media containing 200 nM ISRIB before replacing media containing no drug for 1 hour prior to cell harvest. Cell pellets were washed in PBS, flash frozen in liquid nitrogen and stored at -80C until cell lysis. Each cell pellet was resuspended in 20 mM Hepes, 150 mM KCl, 2 mM TCEP, pH 7.4 + cOmplete EDTA-free protease inhibitor cocktail (Roche). Cells were lysed by bead-based homogenization using Bullet blender (Storm Pro, Next Advance) for 2 x 30 seconds (Setting 10) with 0.15mm zirconium oxide beads. Lysates were clarified by centrifugation at 21,000 x g, 15 min, 4C to remove cellular debris. Protein concentration was determined by BCA and lysates were aliquoted and stored at -80C until use. Bodipy-FL-GDP loaded eIF2 was used as a substrate for in vitro GEF assay. 1 mg/mL protein lysates (unless otherwise described) were treated in vitro with indicated ISRIB or 2BAct concentrations, 25 nM Bodipy-FL-GDP loaded eIF2, 0.1 mM GDP, and 1 mg/mL BSA. Fluorescence decay was measured on a SpectraMax i3x plate reader (Molecular Devices) with the following parameters: plate temperature = 25C; excitation wavelength = 485 nm (15 nm width); emission wavelength = 535 nm (25 nm width); read duration = 30 min at 45 s intervals. Data were analyzed in Prism fitting to a single exponential decay curve to calculate GDP half-lives.

RNA extraction

WT, E198K or N208Y MIN-6 cells were plated in 12-well plates in complete media containing 200 nM ISRIB and grown until 80% confluent. ISRIB-containing media was removed, cells were washed once with PBS before media lacking ISRIB was replaced to the wells. ISRIB was withdrawn for 24 hours prior to cell lysis in RT lysis buffer (Qiagen). RNA extraction using the RNeasy Mini Kit (Qiagen) was performed following the manufacturer's protocol.

Following the indicated animal treatments, the cerebellum, spinal cord, liver, kidney, lung, muscle and spleen were flash frozen in liquid nitrogen prior to RNA extraction. Tissues were treated with RNA-later ICE (Invitrogen #AM7030) for 24 hr at -20C and RNA extraction was performed following the MaxMAX total RNA isolation kit (Invitrogen #AM1830). Briefly, tissues were homogenized in lysis buffer

+ β-mercaptoethanol using a Qiagen TissueLyser II (Qiagen, Hilden, Germany) for 2 x 2-minute intervals at 25 Hz with the addition of one 7 mm stainless steel bead (Qiagen #69990). 200 uL of tissue lysate was used for KingFisher Flex (Thermo Fisher Scientific, Waltham, USA) RNA extraction according to manufacturer's protocol, eluting in 60 uL nuclease-free water. E18.5 embryo lysates were homogenized by mortar pestle grinding in liquid nitrogen prior to bead-based homogenization in RLT RNA extraction buffer using RNeasy Mini Kit (Qiagen, #74104). RNA was extracted following the manufacturer's protocol.

qRT-PCR

cDNA was synthesized from equal amounts of RNA using the High-capacity cDNA Reverse Transcription Kit (Applied Biosystems #4368814) according to the manufacturer's protocol. TaqMan qRT-PCR reactions were performed on Quantstudio 6 Flex (Thermo Fisher Scientific, Waltham, MA, USA) using TaqMan Universal PCR Master Mix (Applied Biosystems #4304437) with the following TaqMan Gene Expression Assays: *Atf3* (Mm00476032_m1), *Chop* (Mm01135937_g1), and *Actin* (Mm00607939_s1) purchased from Applied Biosystems. Gene expression for each sample was measured in triplicate. Fold change of gene expression was performed using the comparative CT (-ΔΔ CT).

Gene expression analysis with nCounter

Multiplex transcript expression levels were measured by nCounter (Nanostring Technologies, Seattle, WA) with a custom panel containing our ISR CLIC genes. 100 - 500 ng of purified total RNA was used for nCounter gene expression analysis as instructed by the manufacturer. Briefly, reporter and capture probes to the genes of interest were hybridized to total RNA at 65°C for 16 hr. Hybridized probes were then captured to the nCounter cartridge prior to imaging and quantification.

nCounter data analysis

Raw counts were background subtracted against negative control probes then normalized against housekeeping genes (*B2m*, *Gapdh*, *Hprt*, *Rpl19*) and internal positive control probes. The counts were $\log_2$-transformed and a Z-score for each gene was calculated by subtracting the overall mean of the control group

from the sample and dividing that result by the SD of all of the measured intensities. ISR pathway activation was measured by taking the averages of the Z-score within each sample. GraphPad Prism (La Jolla, CA) was used to perform statistical analyses utilizing one way ANOVA with Dunnett's Test performed post-hoc to correct for multiple comparisons. Log_2 fold changes of ISR CLIC genes were clustered by correlation-based distance using the heatmap.2 function from the gplots package (R Core Team, 2018).

Bulk RNA-seq and analysis

RNA-seq libraries were prepared using RNA isolated from the cerebellum of 4-month-old male WT and $N208Y^{HOM}$ mice maintained on 2BAct or 3 days after 2BAct withdrawal (n=3 per group) or from the cerebellum of 5-month-old WT and $R191H^{HOM}$ mice (3 males and 2 females for WT and 3 males and 1 female for $R191H^{HOM}$). RNA quality and concentration were assayed using the 5300 Fragment Analyzer System (Agilent Technologies, Santa Clara, CA, USA). RNA-seq libraries were prepared using the NEBNext Ultra II Directional RNA Library Prep Kit for Illumina (New England Biolabs, Ipswich, MA, USA). Paired-end libraries with read length 150 bp were sequenced on an Illumina NovaSeq 6000 sequencer to a mean depth of 36 million reads per library.

RNA-seq library mapping and estimation of expression levels were computed as follows. Reads were mapped using salmon (6), version 1.9.0, to the transcriptome index derived from the mm10 reference mouse genome and the Encode vM12 primary assembly annotation (7).

In order to test for differential expression, for each of the three comparisons, we used sleuth with a design matrix corresponding to two groups (8). Gene set enrichment was done using fgsea using genes ranked in descending order according to the p value and fold change directionality, as reported by sleuth (9).

Single nuclei RNA-seq and analysis

(1) Nuclei preparation and RNAseq using the 10x Genomics platform

The cervical-thoracic region of each spinal cord was dissected, and flash frozen in liquid nitrogen. Nuclei were isolated using the Chromium Nuclei Isolation Kit with RNAse Inhibitor (10x Genomics #1000494) and sample libraries were prepared according to Chromium Next GEM Single Cell 33' Kit v3.1 instructions

(10x Genomics #1000268) to capture 10,000 nuclei per sample. Libraries were pair-end sequenced on an Illumina NovaSeq 6000 sequencer (Illumina, San Diego, CA) under recommended settings. Illumina output was processed using cellranger count (CellRanger 7.0.0, 10x Genomics) to align reads to the mouse mm10 reference genome (including introns), distinguish nuclei-containing droplets from empty ones, and generate a gene-barcode matrix for each sample.

(2) Preprocessing and quality control

We used DecontX (10) to estimate and remove contamination of ambient RNA from each sample by providing filtered and raw output matrices obtained from CellRanger. The gene expression matrices from all samples were subsequently merged in R (version 4.2.1) for further analysis using the Seurat pipeline (version 4.3.0). We filtered out low quality nuclei expressing fewer than 350 unique genes and greater than 5% mitochondrial genes. Data in each post-QC nuclei were log normalized with a scaling factor of 10,000, the 2,000 most variable genes were selected, and a linear transformation was performed to scale the data before performing dimensionality reduction by PCA. 20 principal components were used for graph-based clustering with a resolution of 0.1. These methods are implemented as the NormalizeData (method = 'LogNormalize'), FindVariableFeatures, ScaleData, RunPCA, FindNeighbours, FindClusters functions in Seurat. To remove potential doublets or multiplets, we used these identified clusters to perform cluster-based doublet detection for each sample individually using scDblFinder (11) and removed flagged barcodes from downstream analysis. Variable feature selection, scaling, PCA, graph-based clustering was performed as described above for all singlets and this filtered dataset is used for all subsequent analysis. Finally, we generated UMAP plots using Seurat's RunUMAP function to visualize the data.

(3) Cluster annotation, reference UMAP projection, and cell label transfer

Clusters were annotated by evaluating the expression of marker genes for spinal cord and brain cell types canonically used in literature (12, 13). The DotPlot function from the Seurat package was used to visualize the average expression and percentage of cells expressing each gene in each cluster. To annotate nuclei from the untreated WT and R191H^{HOM} spinal cords, we used the FindTransferAnchors function to project

post-QC nuclei (processed and filtered as described above) onto the N208Y UMAP structure, and the TransferData function to classify cell types using the annotated N208Y dataset as a reference, both implemented in Seurat using default parameters. To visualize the R191H dataset on the same UMAP structure as the N208Y dataset, Seurat's MapQuery function was used.

(4) ISR module score calculation

The ISR CLIC genes plus *Fgf21* were used to obtain an ISR gene set expression score using the AddModuleScore function in Seurat, which calculates the average expression level of this gene program against a set of expression-level-adjusted control genes for each cell. Results were visualized using complex_dotplot_single from the R package plot1cell.

(5) Cell type composition analysis

We used the plot_cell_fraction function from plot1cell to visualize cell proportions across groups for each cluster. To test for statistical differences in cell type proportions between groups, the propeller function (14) from the speckle R package was used with default parameters. Briefly, cell type proportions are calculated for each biological replicate, a logit transformation is performed on the matrix of proportions, a linear model for each cell type is fit using the limma framework, and an F-test (ANOVA) is used.

(6) Maturation trajectory inference

Upon subsetting the full object to OPC, MFOL, and MOL clusters, the Seurat pipeline was again used to find variable features, scale data, run PCA, and find neighbors (using 10 principal components) and clusters (resolution of 0.1). We then computed a single cell pseudotime trajectory using the slingshot algorithm (version 2.4.0)(15), using the calculated UMAP embeddings and cell type labels as input. OPCs were pre-defined as the starting clusterin this analysis. A violin plot was then used to show the distribution of nuclei in each group along the pseudotime trajectory.

(7) Pseudo bulking and gene set enrichment analysis

The AggregateExpression function from Seurat was used to calculate the summed expression values for each cluster for every sample. For visualization, counts were normalized to library size, a prior.count of 5 added, and log2 transformed. Heatmaps were generated using the ComplexHeatmap R package and plotted as log2 fold change over the average expression of the indicated control group. For differential gene expression analysis, we used the limma-voom pipeline to normalize data, define contrasts, and to fit a linear model. These results were used as input for the seas function (with parameters: methods = 'camera', feature.max.padj = 0.05, feature.min.logFC = 0.5, inter.gene.cor = 0.01, feature.bias = "size") in the sparrow package to test for enrichment of Hallmark gene sets from the Molecular Signatures Database (16).

MTBE-LLE extraction for cholesterol measurement of tissues

Following 2BAct withdrawal, 5-month-old female WT and N208Y^{HOM} mice (and corresponding 2BAct-maintained controls) were euthanized with CO_2, and cerebellum and spinal cord were freshly collected via liquid nitrogen cooled clamps and stored at -80 °C. Lipids were extracted from tissues using methyl-tert-butyl ether liquid-liquid extraction (MTBE-LLE) adopted from Matyash et al.(17). In brief, 25 mg of tissues were homogenized using a Cryomill (Retsch) at 25Hz rpm for 30 sec with constant liquid nitrogen cooling, then 800 μL of 50/50 v/v MeOH/H_2O containing 1ppm internal standards (EquiSplash, Avanti Polar Lipids) was added. The homogenate was vortexed for 30 sec, incubated on ice for 10 min, and 800 μL MTBE was added. Samples were vortexed for 30 sec, incubated on ice for 15 min, and then centrifuged at 3500 RPM for 10 min at 4°C. Lipids, partitioned in the top layer, were collected into a separate vial, and the extraction process was repeated via addition of 600 μL of MTBE. After vortexing, incubation and centrifugation, the second organic layer was collected and combined in the first extract, dried under nitrogen at 4°C, and resuspended in 200 μL of IPA.

LC-MS for cholesterol measurement

Cholesterol was measured in positive ion mode using LC-MS, consisting of a Vanquish UPLC coupled to a Q-Exactive Plus mass spectrometer. Cholesterol was separated from other lipids using a Thermo Scientific

Hypersil Gold C18 (3 µm, 2.1mm x 100mm) at a flow rate of 0.3 mL/min. Mobile phase A was 5 mM ammonium acetate in H_2O, and mobile phase B was 5 mM ammonium acetate in MeCN/ H_2O (95/5,v/v). The gradient was t=-2, 60%B, t=0, 60%B, t=3, 100%B, t= 8, 100%B, t= 12, 50%B. The Q-Exactive Plus was operated in APCI mode, using data-dependent acquisition with the following settings: resolution = 140,000, AGC target = $3x10^6$, maximum IT = 100 ms, scan range = 50-750. The MS2 parameters were as follows: resolution = 17,500, AGC target = $3x10^6$, maximum IT = 150 ms, loop count =3, isolation window = 1 m/z, (N)CE = 20,30,40, underfill ratio = 1%, Apex trigger = 5-30s, dynamic exclusion =10s.

Raw files were converted to mzML files using msconvert from ProteoWizard (18) and analyzed using MAVEN2 software (19). Identification of cholesterol was performed by matching retention time and fragmentation spectra compared to an authentic cholesterol standard. Absolute quantification of cholesterol in tissue was performed by normalizing the peak area of endogenous cholesterol to the cholesterol-d7 standard (Avanti Polar Lipids) in every sample. Two-way ANOVA analysis was done to compare across conditions in Prism.

Isolation, characterization, and relative in vitro cholesterol synthesis rate measurement of mouse embryonic fibroblasts

(1) Isolation and culture $N208Y^{HOM}$ mouse embryo fibroblasts (MEFs) were isolated from embryos at embryonic day 13.5 as described (20) with some modifications. Briefly, each embryo was individually processed by first removing the liver and heart before mincing into small pieces followed with trypsin digestion. Cells were mechanically dissociated by pipetting before inhibition the trypsin reaction with complete medium (Dulbecco's modified Eagle's medium (DMEM, Gibco) supplemented with 10% fetal bovine serum (FBS, Sigma) and 1X antibiotic-antimycotic solution (Gibco) and 200nM 2BAct). The cell suspension was cultured in a 15 cm petri dish housed in an incubator at 37°C, 5% CO2, 3% O2 and passaged every 2-3 days with trypsin.

(2) Gene expression analysis

N208YHOM MEFs were seeded in DMEM with 10% FBS with 200 nM 2BAct. Cells were washed 3 times in PBS prior to replacing cells with media containing with or without 200 nM 2BAct for 6 hours. Cells were collected in RNA lysis buffer (Applied Biosystems A27828) and RNA was extracted following the manufacturer's protocol for bulk RNA sequencing. Gene expression of ISR CLIC and cholesterol synthesis genes from N208Y^{HOM} MEFs were measured by bulk RNAseq, using RNA extraction, library preparation, and bulk RNAseq analysis methods described above.

(3) Deuterium tracing

For determining $^{2}H_2O$ incorporation into cholesterol, MEFs were seeded in DMEM with 10% FBS supplemented with 1X NEAA (Sigma) and 55nM beta mercaptoethanol (Gibco) with 200 nM 2BAct prior to exchanging to 10% lipoprotein-depleted serum media (LPDS, Kalen Biomedical, LLC) for 24 hours. Cells were washed 3 times in PBS prior to replacing cells with media containing 10% $^{2}H_2O$ (Sigma) or 10% cultured water (Corning) with 10% LPDS with or without 200 nM 2BAct for 24 hours. For Western blots, cells were washed with cold PBS, prior to lysing cells in RIPA buffer. N208Y^{HOM} MEFs were characterized for eIF2B1α, ATF4, and eIF2α protein expression as described above. For deuterium tracing, cells were washed with 1mL PBS and quenched with 1 mL -20°C LC-MS isopropanol. The supernatant was dried under nitrogen and resuspended in 200 µL of LC-MS grade isopropanol. Free (non-esterified) cholesterol was identified based on the MS^{1} accurate mass of the [M-H_2O+H]+ adduct, MS^{2} fragmentation pattern, and retention time of an authentic cholesterol standard. Deuterium label incorporation into cholesterol was determined using MAVEN2, which extracted the isotopic envelopes, and corrected for the natural abundance of ^{13}C and ^{2}H isotopes with functionality adopted from the IsoCorrectoR R package (21). The *de novo* cholesterol synthesis was quantified as the fraction of the total cholesterol isotopologues containing one or more deuteria, post correction.

In vivo **cholesterol synthesis rate determination**

The cholesterol synthesis rate in 5-month-old WT and N208Y^{HOM} mice (either maintained on 2BAct or subjected to 3 day 2BAct withdrawal) was determined by providing free access to 6% $^{2}H_2O$ in drinking

water for 2 weeks prior to tissue harvest. Spinal cord and cerebellum were quickly dissected and frozen via clamping with a liquid nitrogen cooled clamp. Samples were prepared via homogenization at cryo temperature, followed-up with MTBE-LLE, and subsequently analyzed via LC-MS. Peaks were extracted and corrected for natural isotope abundance with MAVEN2, and *de novo* synthesis was determined as described above.

Supplementary References

1. Wong YL, LeBon L, Basso AM, Kohlhaas KL, Nikkel AL, Robb HM, et al. eIF2B activator prevents neurological defects caused by a chronic integrated stress response. *eLife.* 2019;8.
2. Hao Q, Heo JM, Nocek BP, Hicks KG, Stoll VS, Remarcik C, et al. Sugar phosphate activation of the stress sensor eIF2B. *Nat Commun.* 2021;12(1):3440.
3. Kammoun M, Maas E, Criem N, Gribnau J, Zwijsen A, and Vermeesch JR. RLIM enhances BMP signalling mediated fetal lung development in mice. *bioRxiv.* 2018:507921.
4. Traka M. The DTA Mouse Model for Oligodendrocyte Ablation and CNS Demyelination. *Methods Mol Biol.* 2019;1936:295-310.
5. Bankhead P, Loughrey MB, Fernandez JA, Dombrowski Y, McArt DG, Dunne PD, et al. QuPath: Open source software for digital pathology image analysis. *Sci Rep.* 2017;7(1):16878.
6. Patro R, Duggal G, Love MI, Irizarry RA, and Kingsford C. Salmon provides fast and bias-aware quantification of transcript expression. *Nature methods.* 2017;14(4):417-9.
7. Mudge JM, and Harrow J. Creating reference gene annotation for the mouse C57BL6/J genome assembly. *Mamm Genome.* 2015;26(9-10):366-78.
8. Pimentel H, Bray NL, Puente S, Melsted P, and Pachter L. Differential analysis of RNA-seq incorporating quantification uncertainty. *Nature methods.* 2017;14(7):687-90.
9. Korotkevich G, Sukhov V, and Sergushichev A. Fast gene set enrichment analysis. *bioRxiv.* 2019:060012.
10. Yang S, Corbett SE, Koga Y, Wang Z, Johnson WE, Yajima M, et al. Decontamination of ambient RNA in single-cell RNA-seq with DecontX. *Genome Biol.* 2020;21(1):57.
11. Germain PL, Lun A, Garcia Meixide C, Macnair W, and Robinson MD. Doublet identification in single-cell sequencing data using scDblFinder. *F1000Res.* 2021;10:979.
12. Russ DE, Cross RBP, Li L, Koch SC, Matson KJE, Yadav A, et al. A harmonized atlas of mouse spinal cord cell types and their spatial organization. *Nat Commun.* 2021;12(1):5722.

13. Zeisel A, Hochgerner H, Lonnerberg P, Johnsson A, Memic F, van der Zwan J, et al. Molecular Architecture of the Mouse Nervous System. *Cell.* 2018;174(4):999-1014 e22.

14. Phipson B, Sim CB, Porrello ER, Hewitt AW, Powell J, and Oshlack A. propeller: testing for differences in cell type proportions in single cell data. *Bioinformatics.* 2022;38(20):4720-6.

15. Street K, Risso D, Fletcher RB, Das D, Ngai J, Yosef N, et al. Slingshot: cell lineage and pseudotime inference for single-cell transcriptomics. *BMC Genomics.* 2018;19(1):477.

16. Liberzon A, Birger C, Thorvaldsdottir H, Ghandi M, Mesirov JP, and Tamayo P. The Molecular Signatures Database (MSigDB) hallmark gene set collection. *Cell Syst.* 2015;1(6):417-25.

17. Matyash V, Liebisch G, Kurzchalia TV, Shevchenko A, and Schwudke D. Lipid extraction by methyl-tert-butyl ether for high-throughput lipidomics. *J Lipid Res.* 2008;49(5):1137-46.

18. Chambers MC, Maclean B, Burke R, Amodei D, Ruderman DL, Neumann S, et al. A cross-platform toolkit for mass spectrometry and proteomics. *Nat Biotechnol.* 2012;30(10):918-20.

19. Seitzer P, Bennett B, and Melamud E. MAVEN2: An Updated Open-Source Mass Spectrometry Exploration Platform. *Metabolites.* 2022;12(8).

20. Durkin ME, Qian X, Popescu NC, and Lowy DR. Isolation of Mouse Embryo Fibroblasts. *Bio Protoc.* 2013;3(18).

21. Heinrich P, Kohler C, Ellmann L, Kuerner P, Spang R, Oefner PJ, et al. Correcting for natural isotope abundance and tracer impurity in MS-, MS/MS- and high-resolution-multiple-tracer-data from stable isotope labeling experiments with IsoCorrectoR. *Sci Rep.* 2018;8(1):17910.
