# Supplementary material for: Chronic integrated stress response causes dysregulated cholesterol synthesis in white matter disease
Source: JCI Insight. 2025 Jul 15;10(16):e188459. doi: 10.1172/jci.insight.188459 (PMC12406721; doi:10.1172/jci.insight.188459)

Full unedited blot for Figure 1A

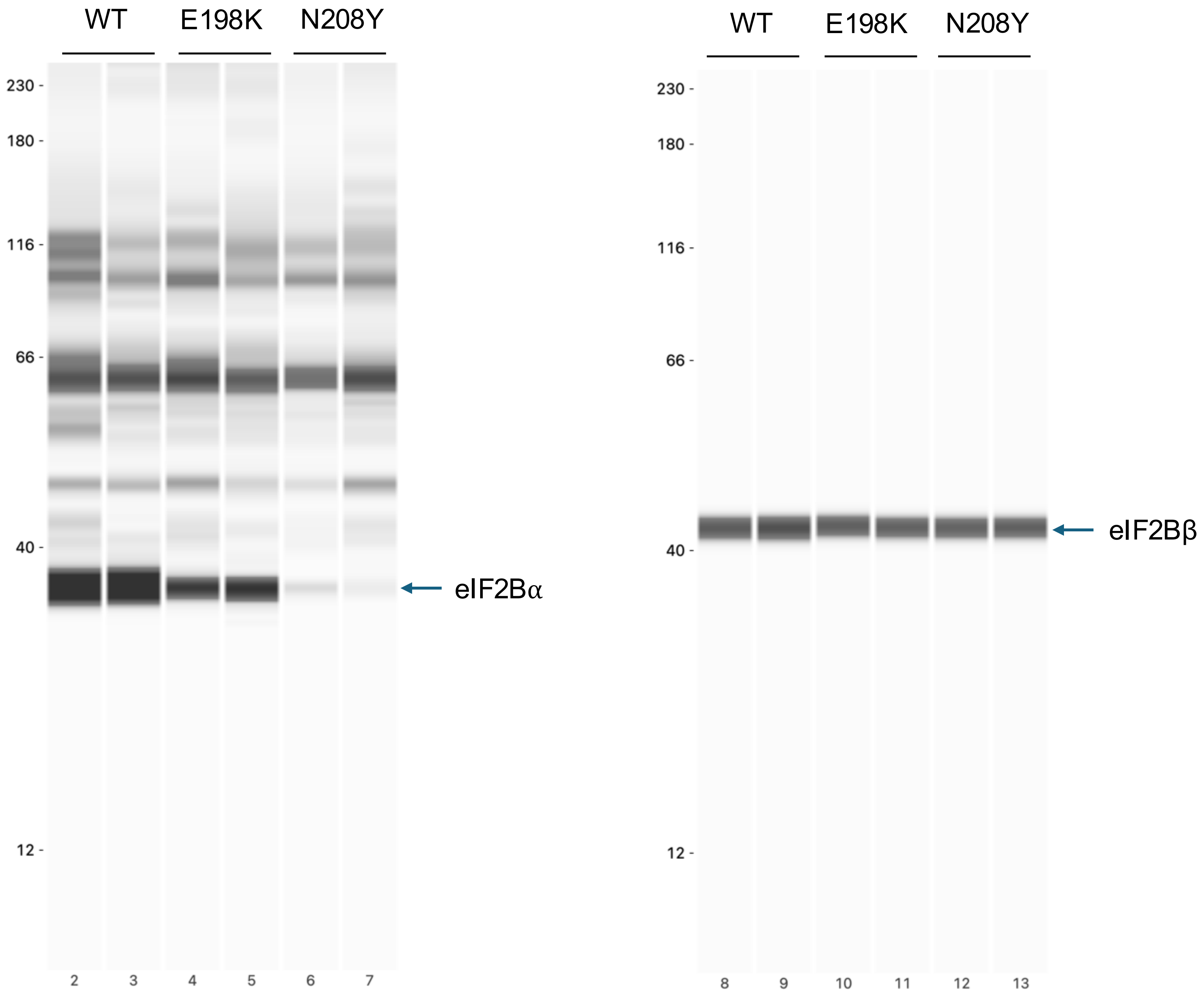

Full unedited blot for Figure 1A

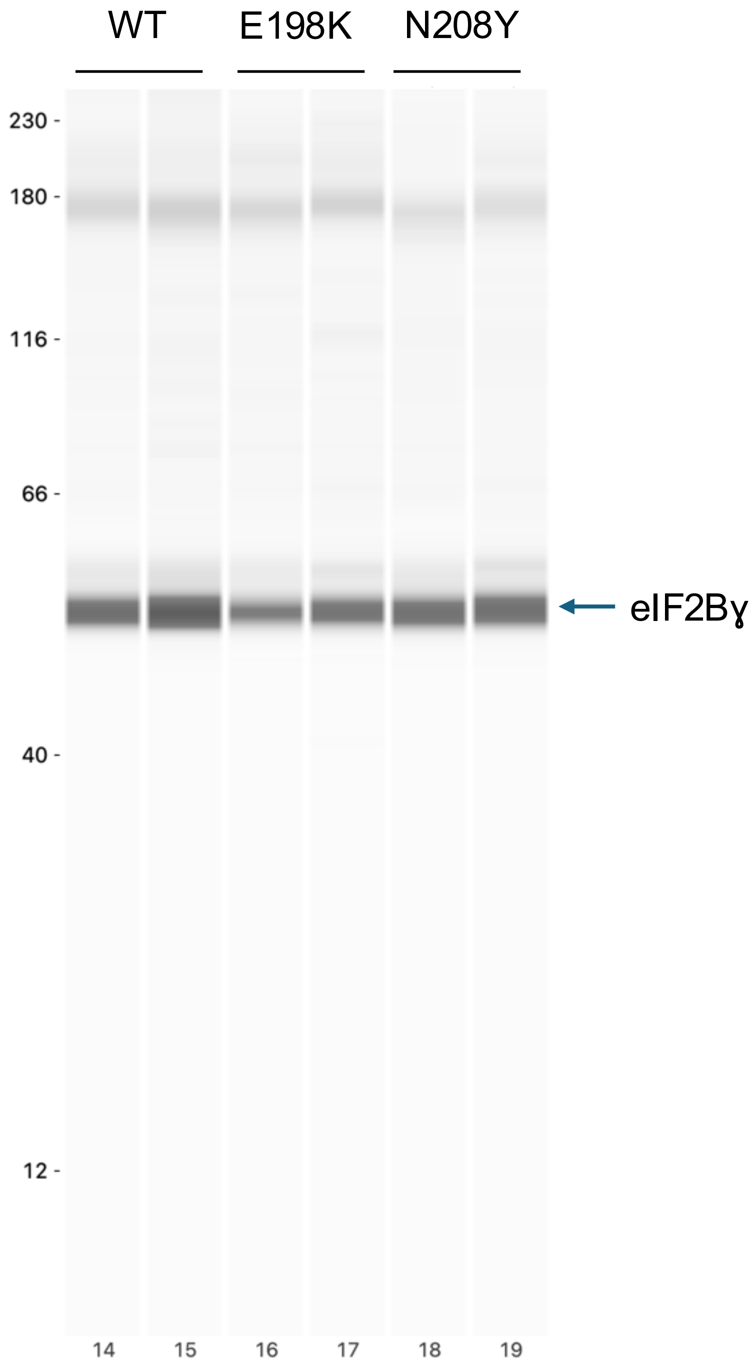

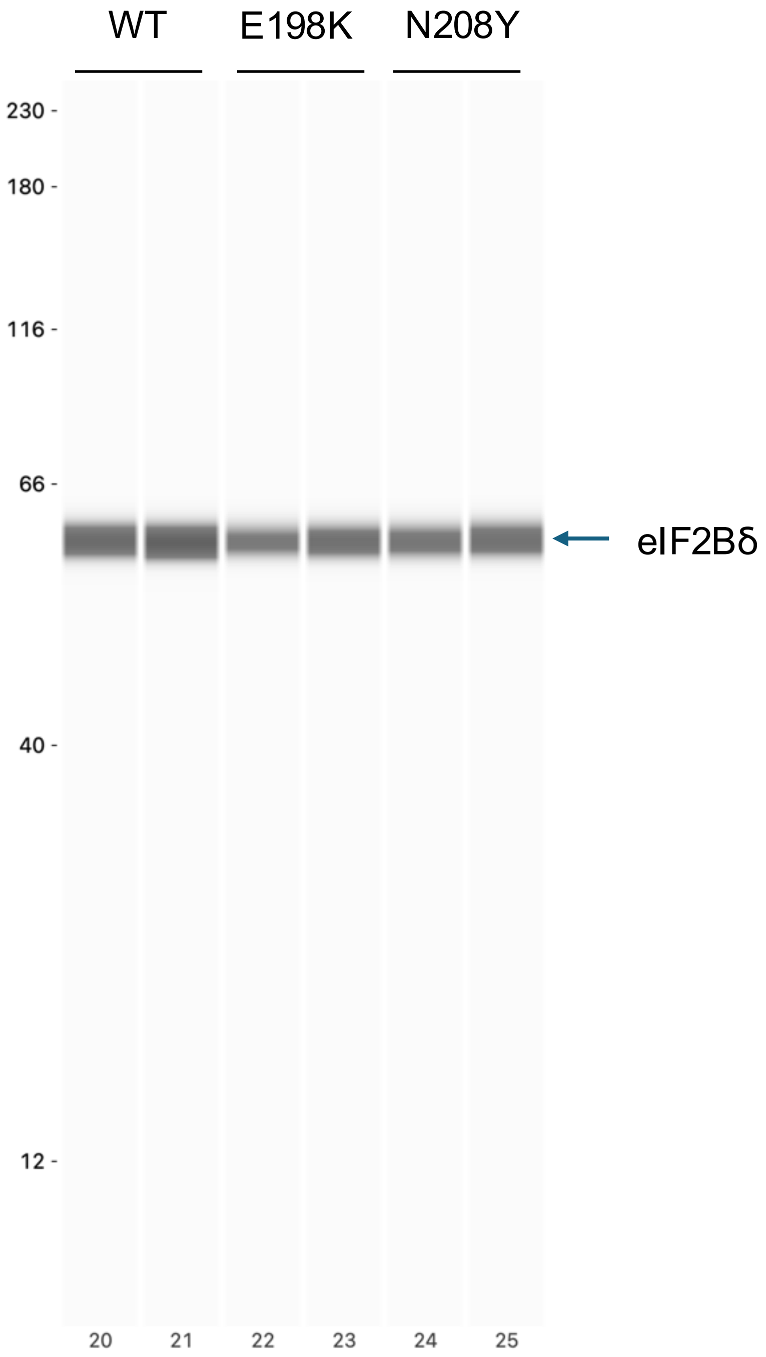

Full unedited blot for Figure 1A

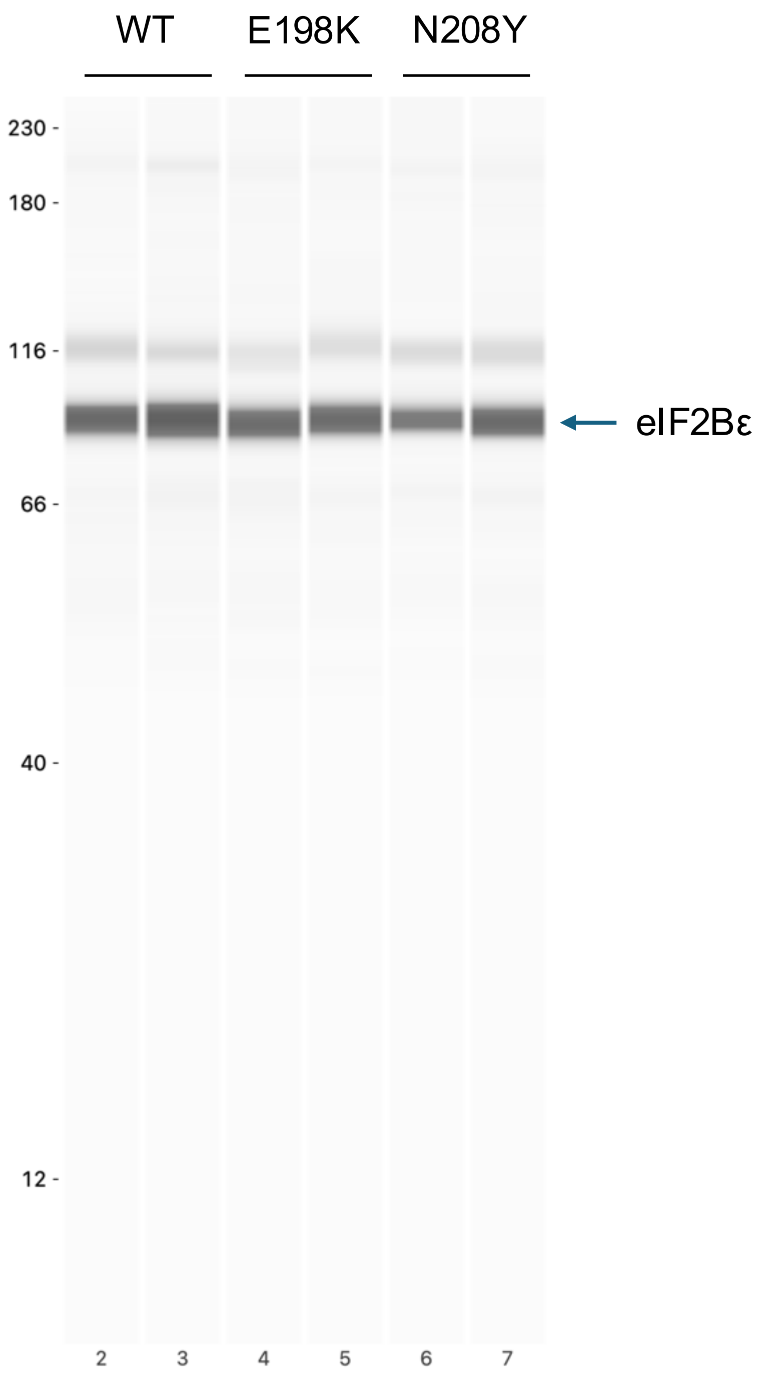

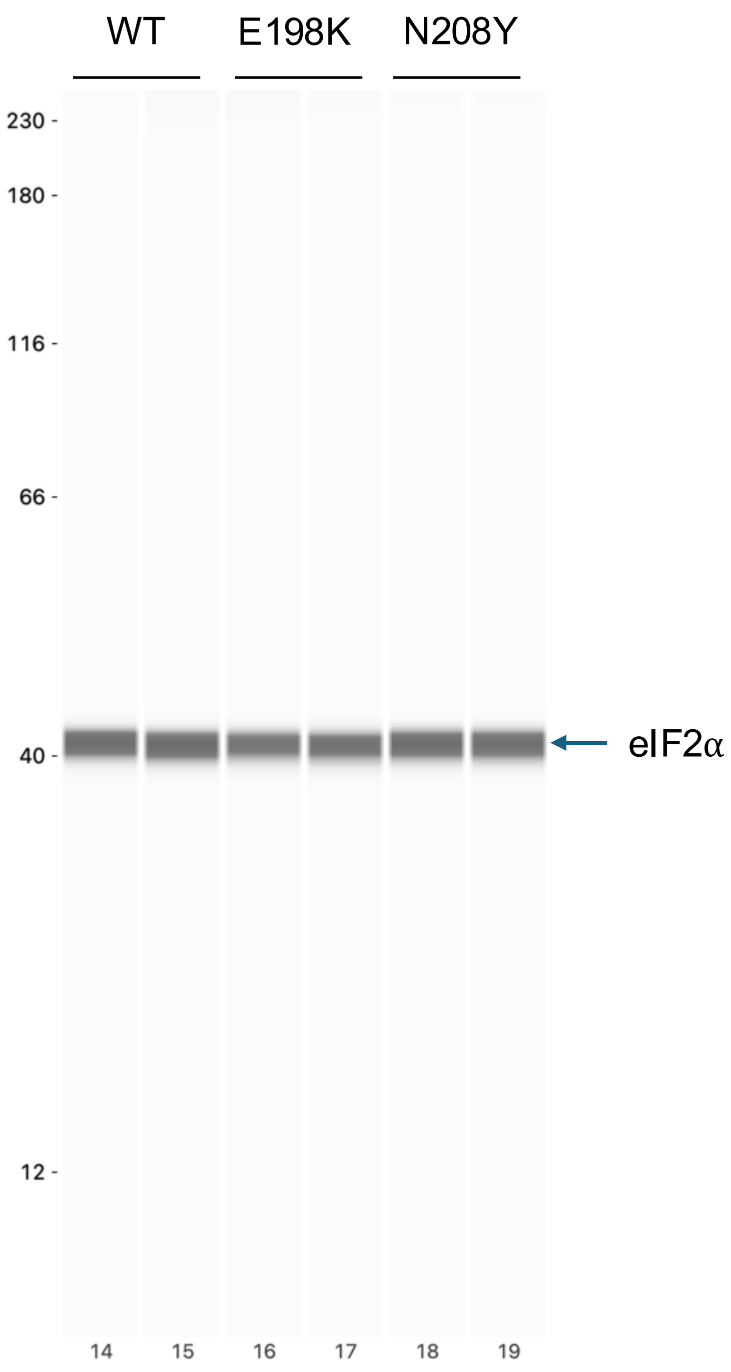

Full unedited blot for Figure 1F

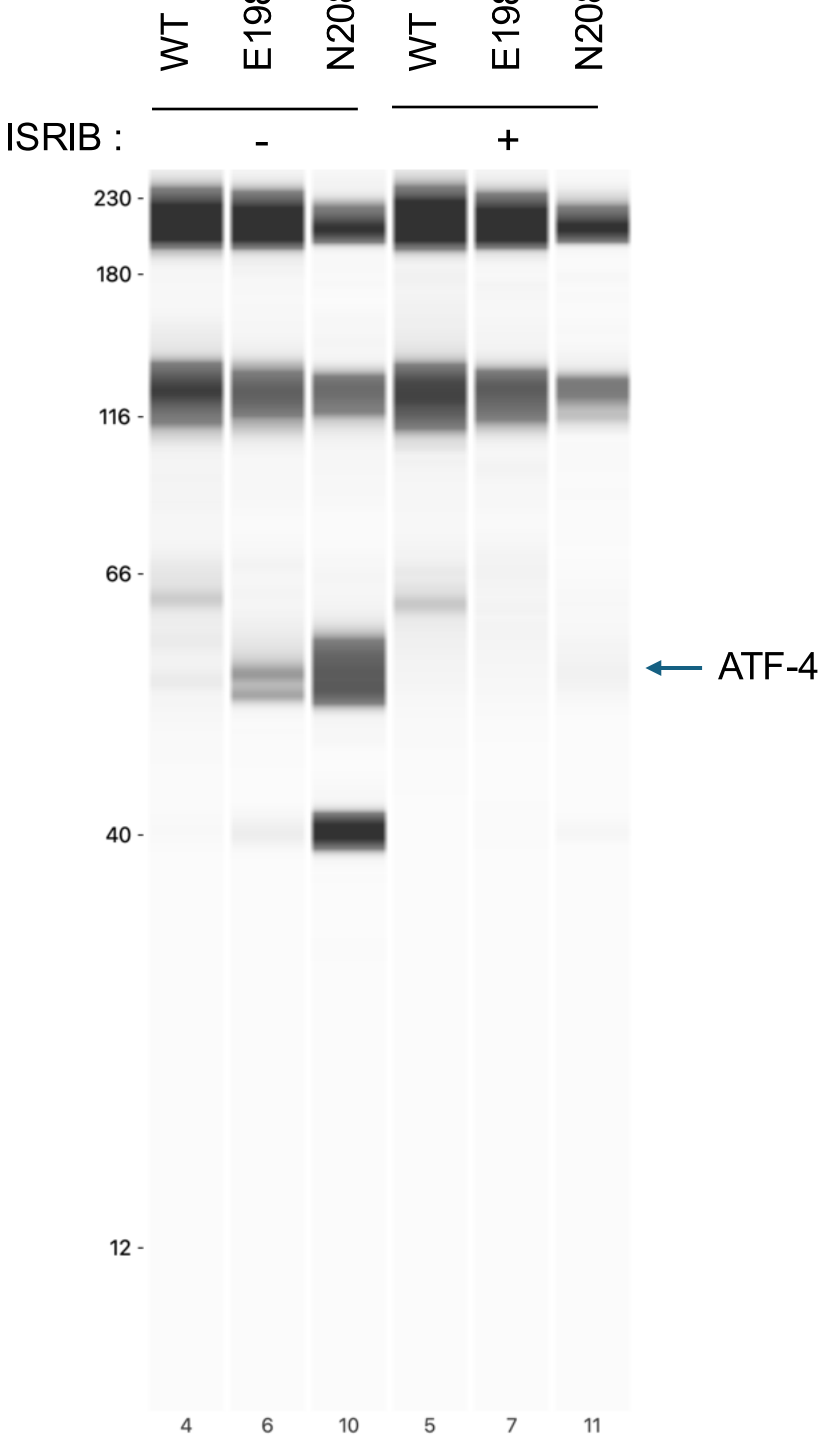

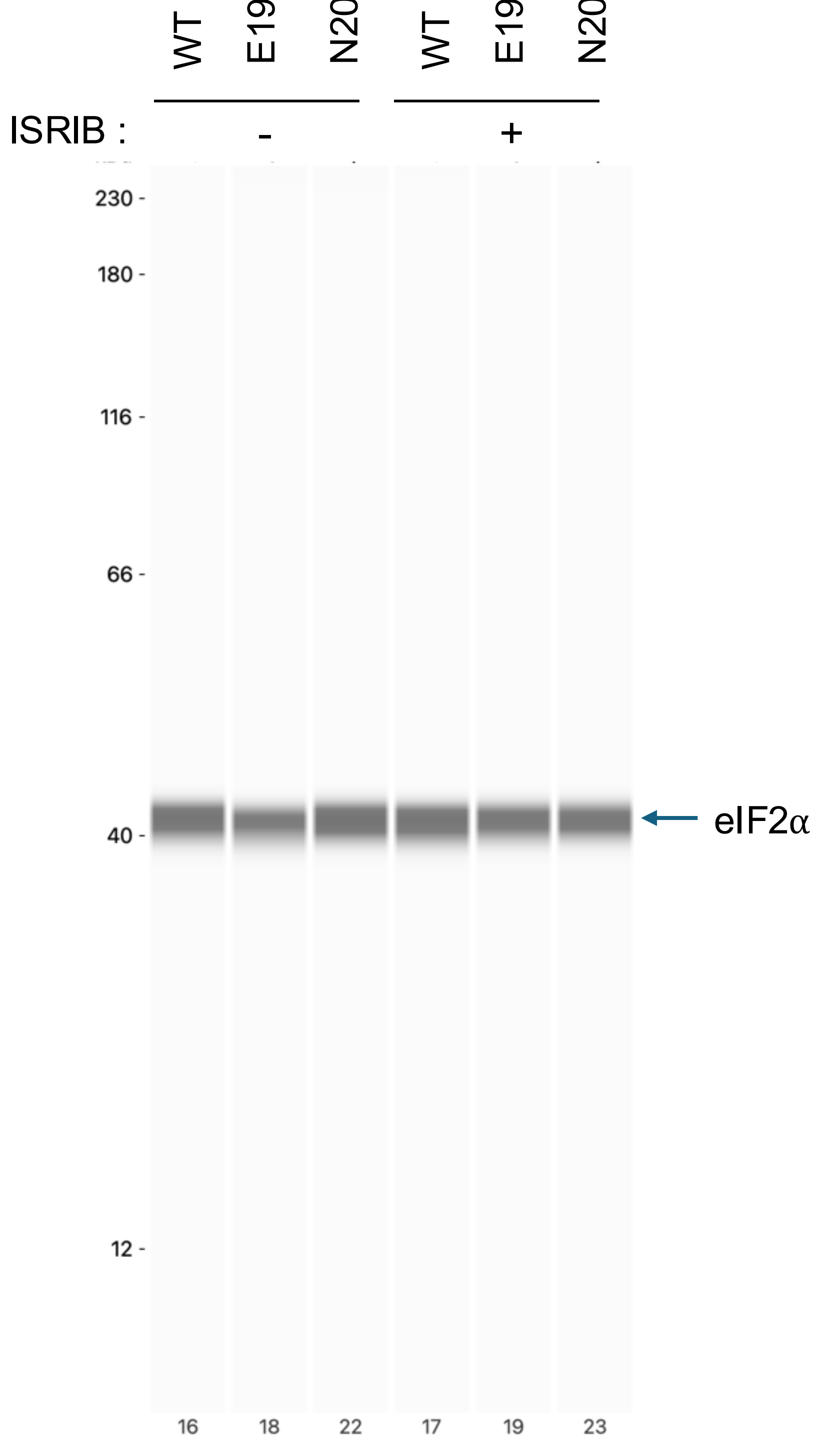

Full unedited blot for Figure 2F

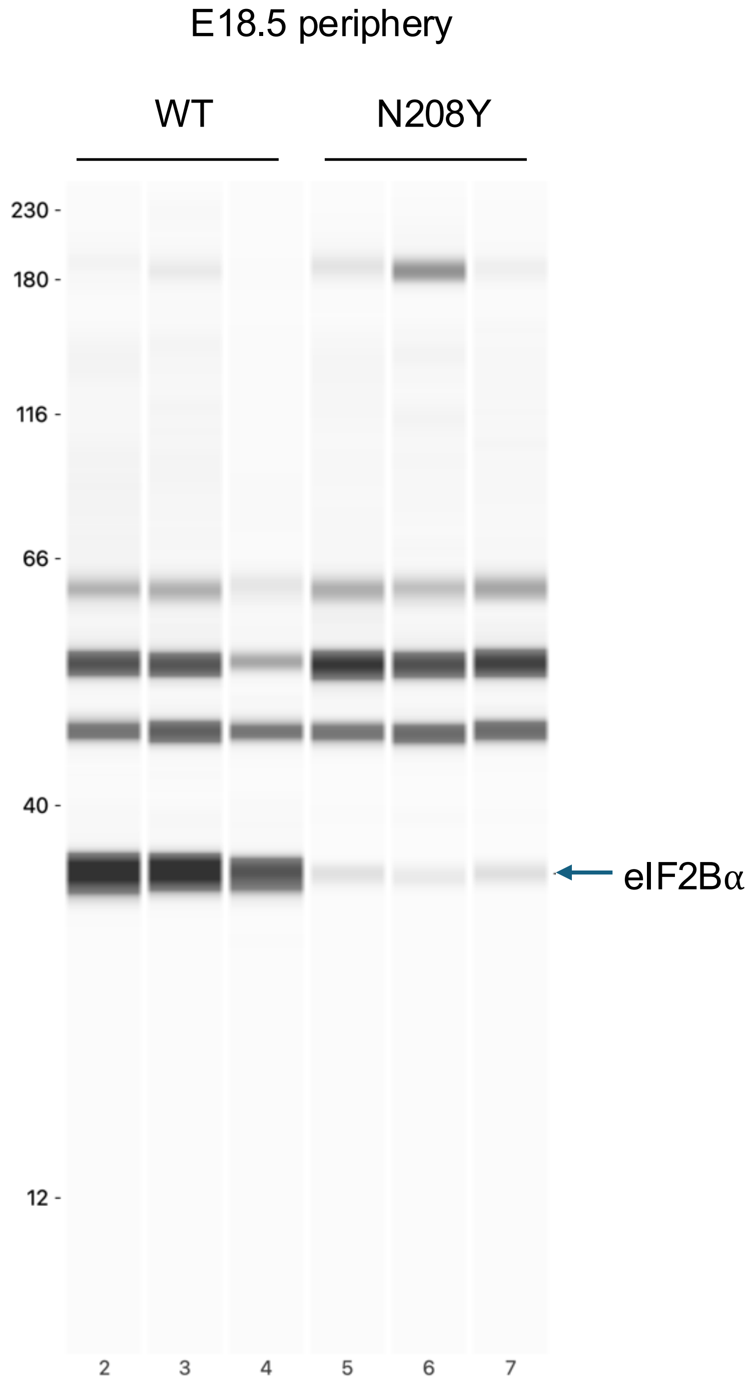

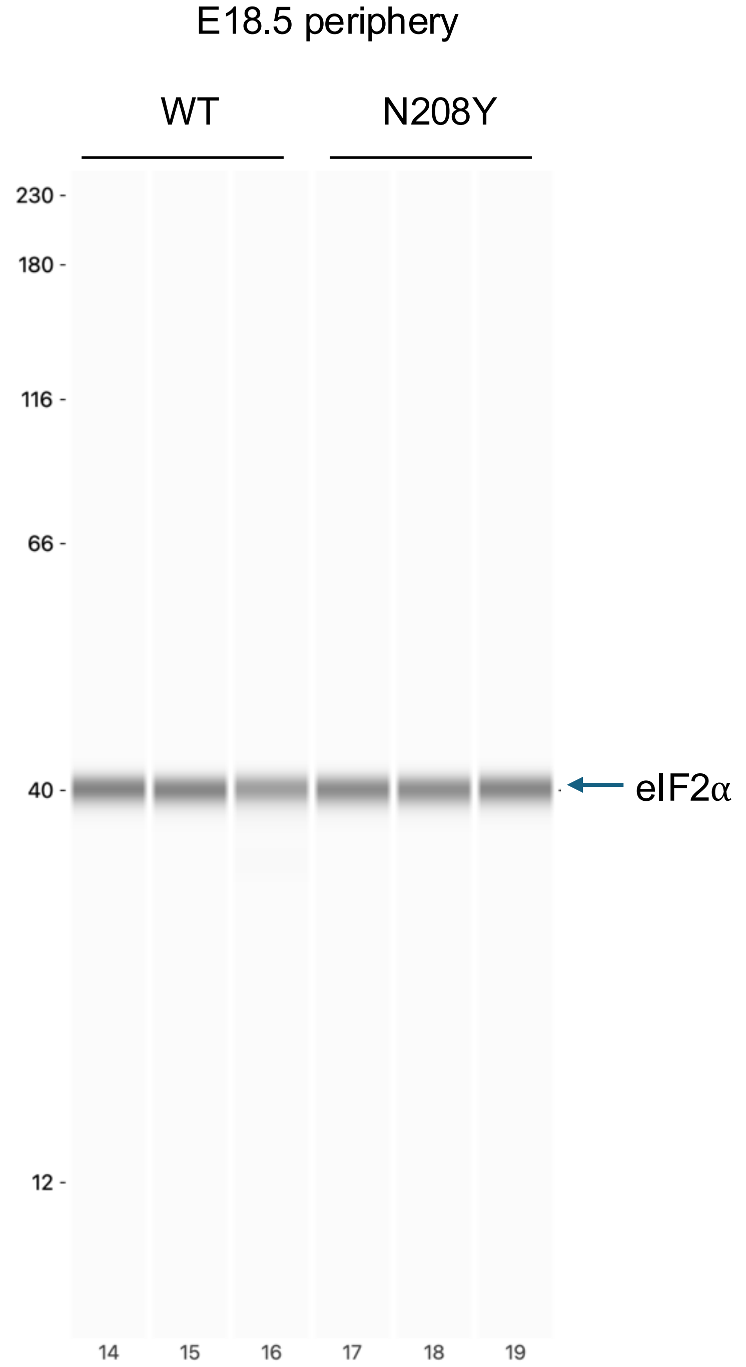

Full unedited blot for Figure 2G

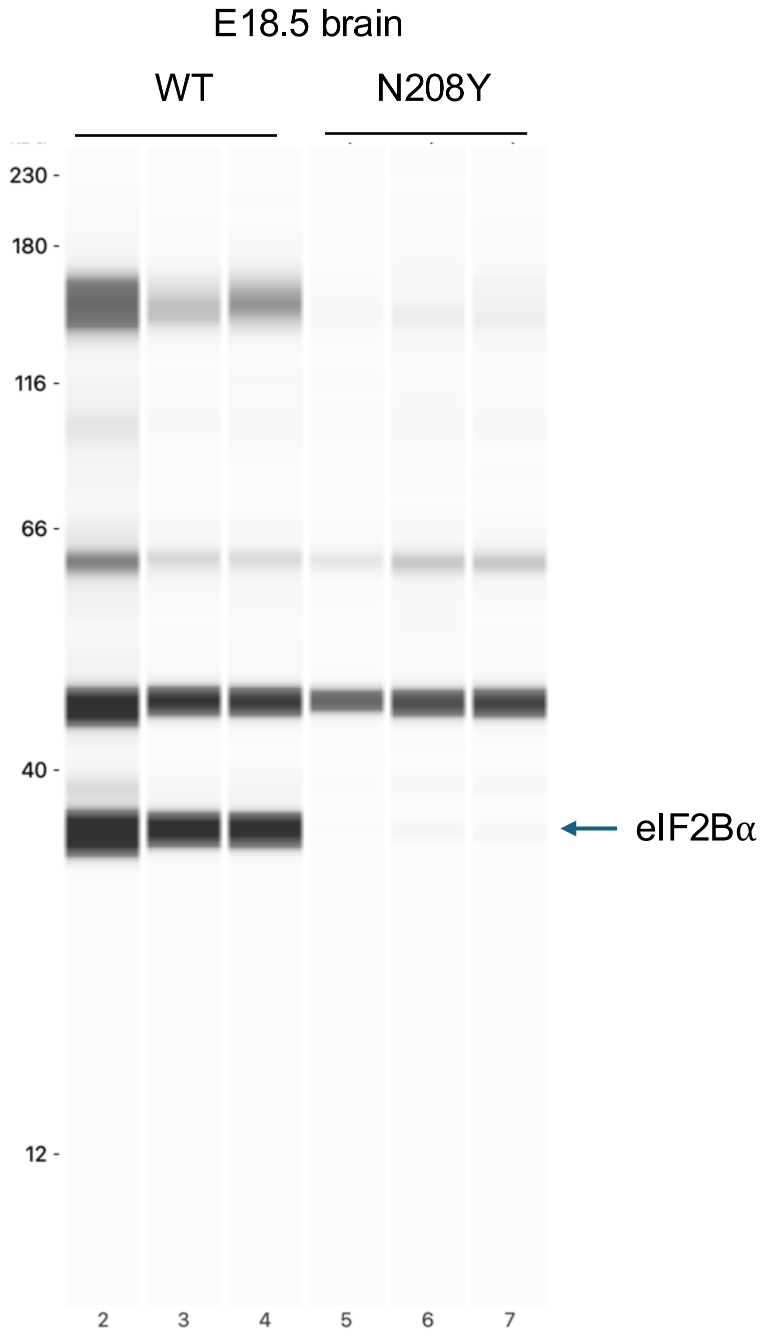

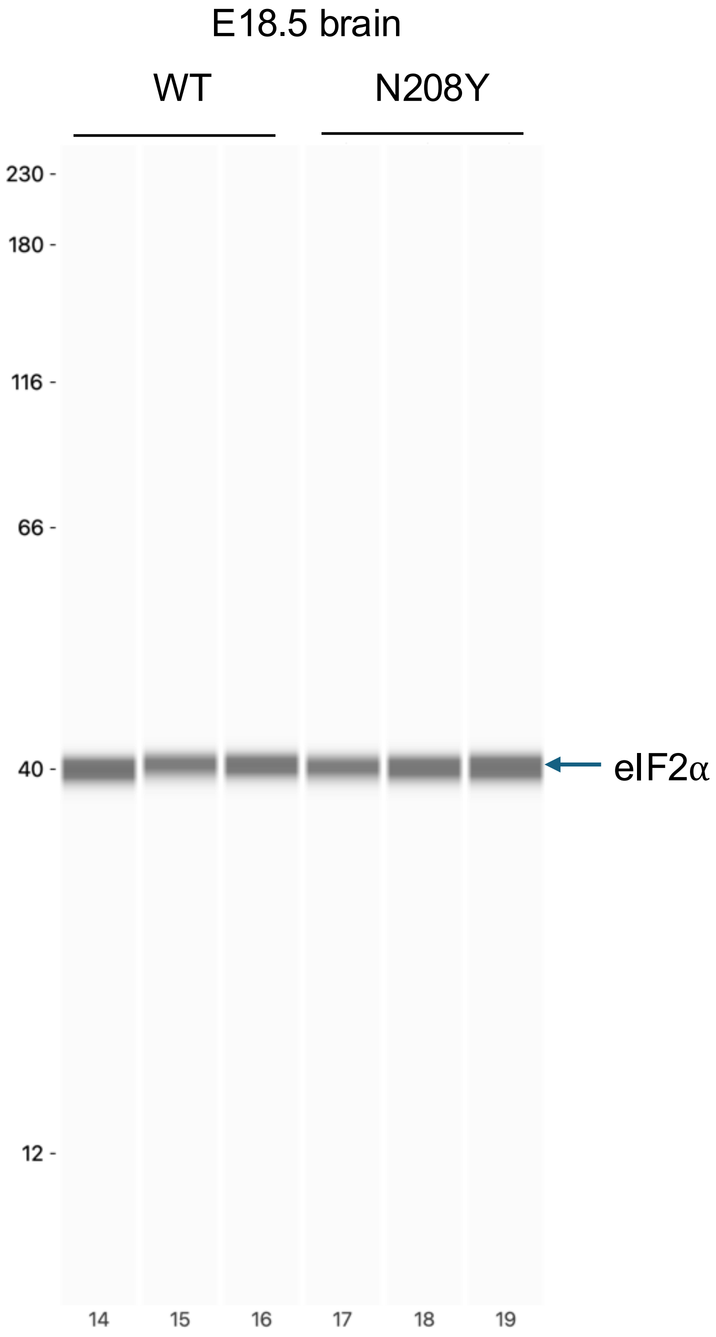

Full unedited blot for Figure 7C

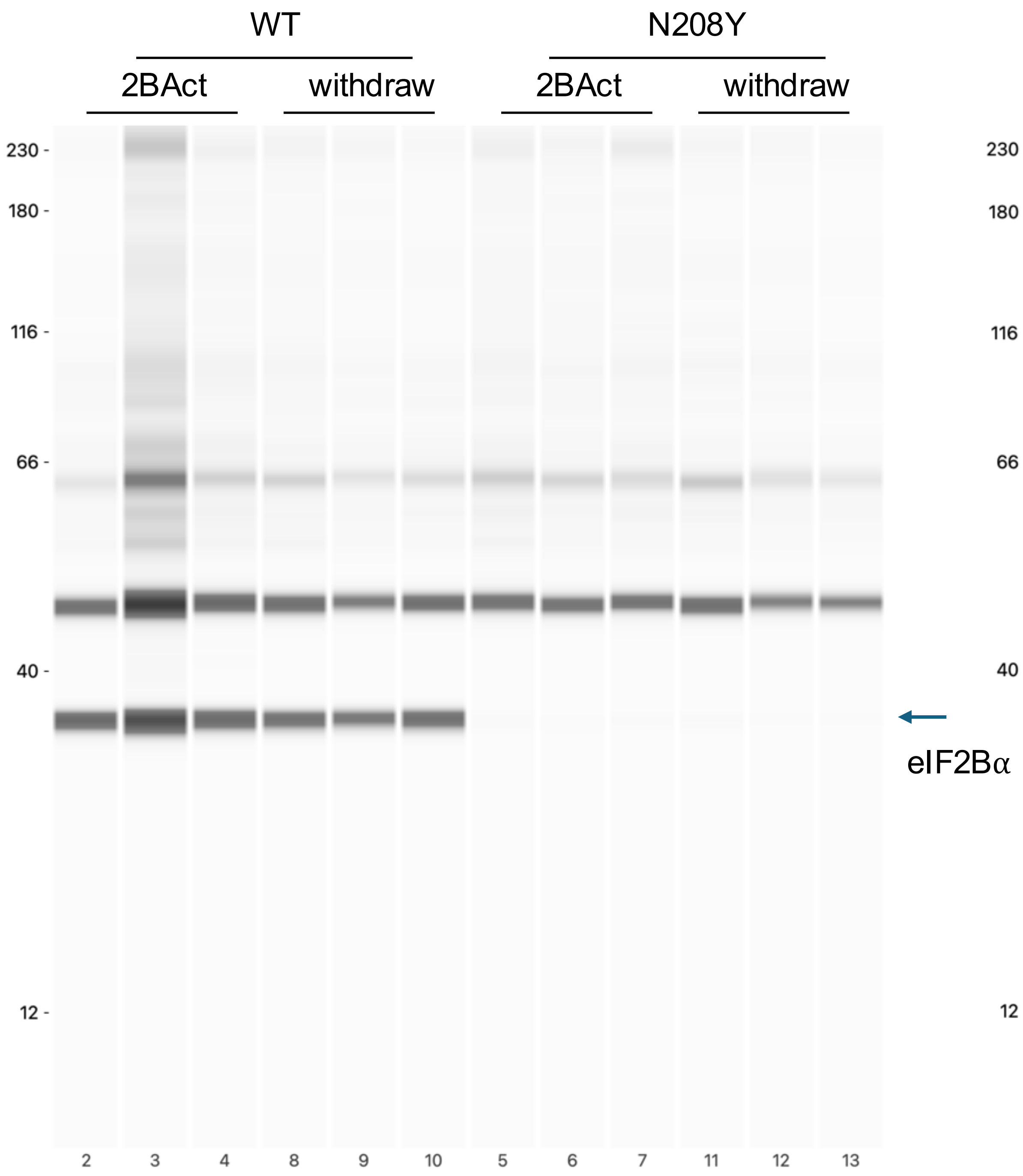

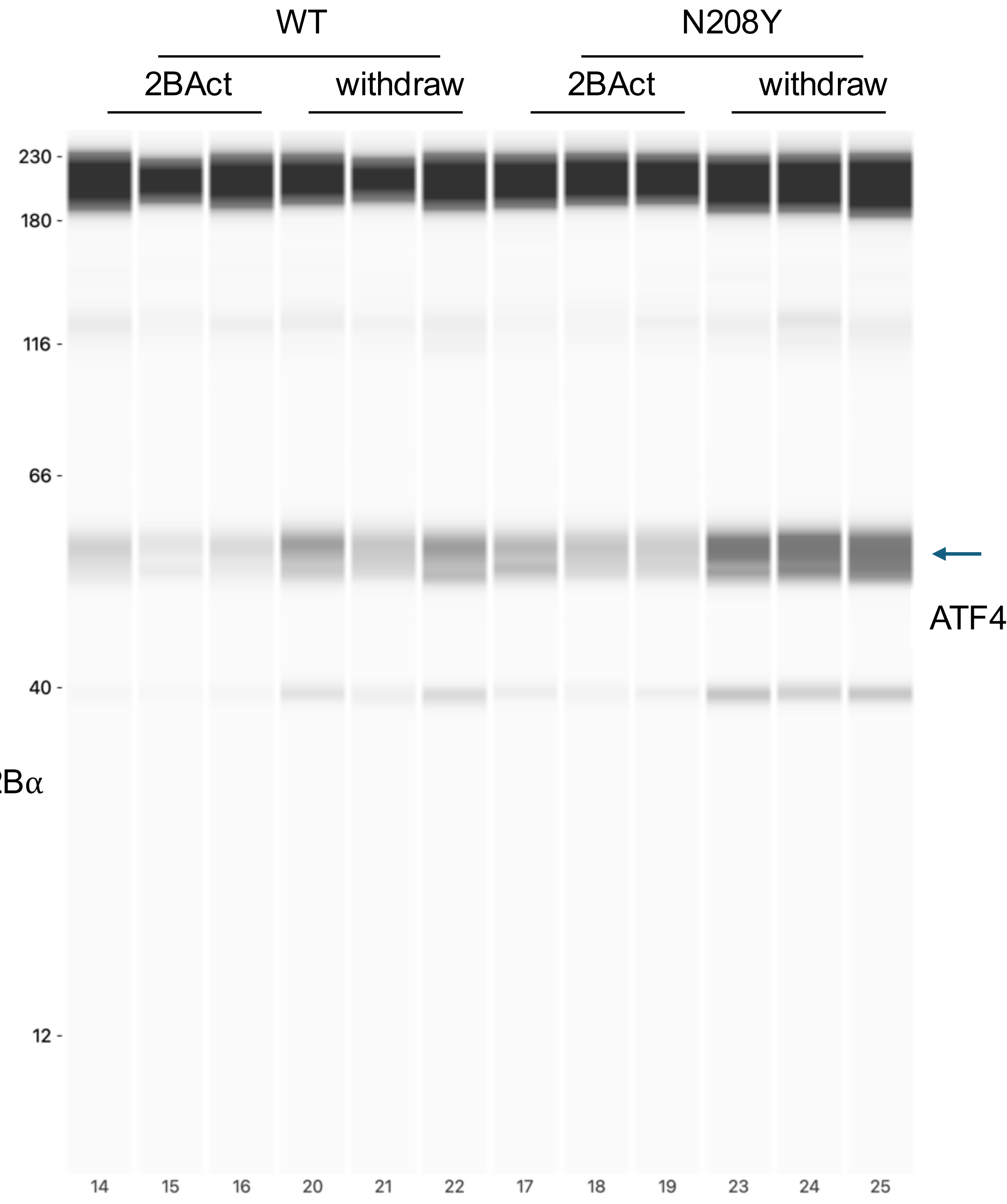

Full unedited blot for Figure 7C

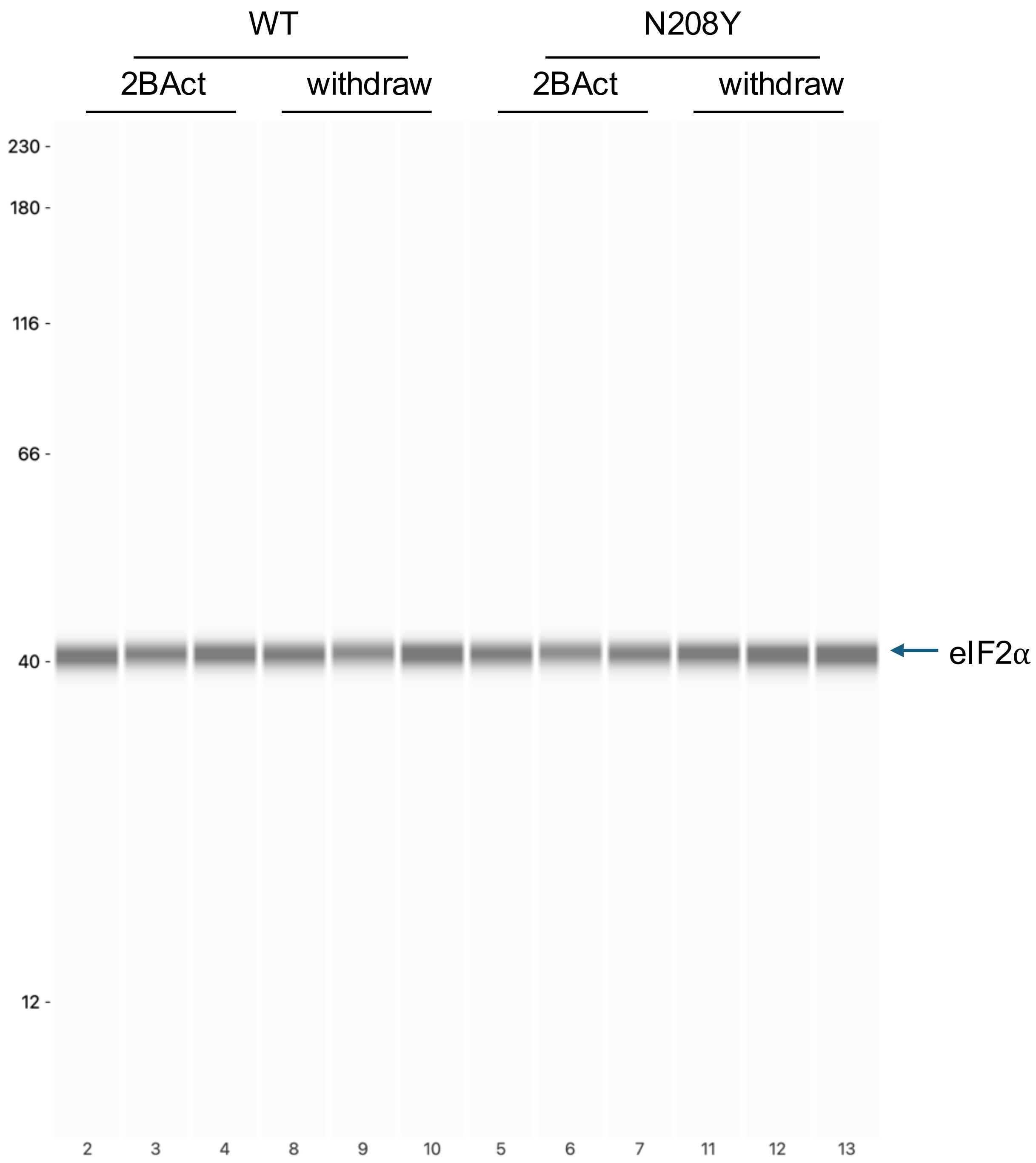

Full unedited blot for Supplement Figure 1B

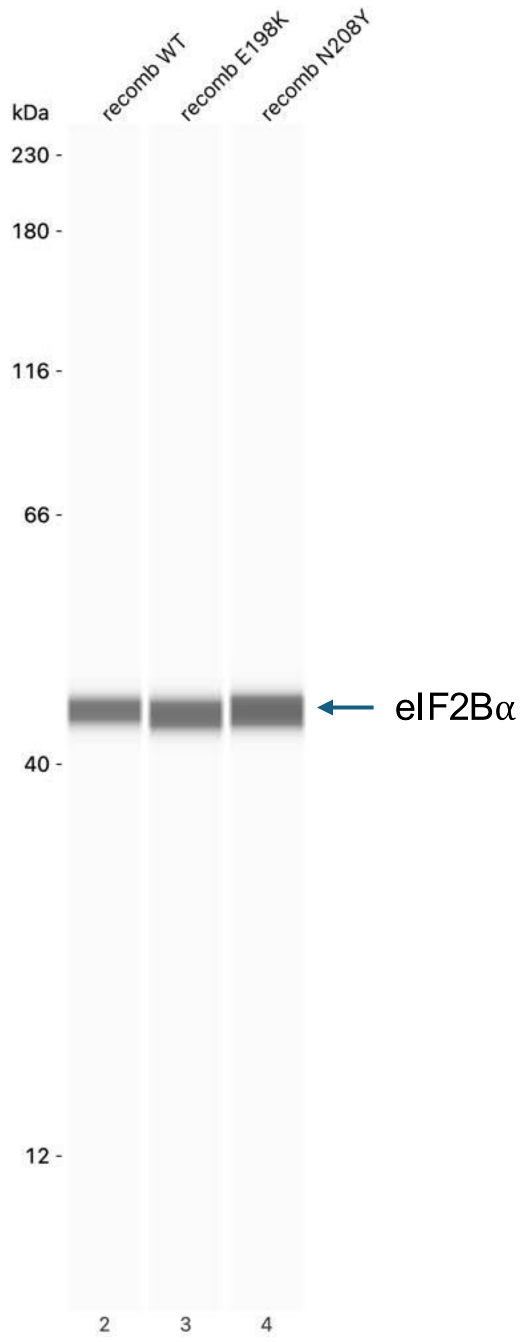

Full unedited blot for Supplement Figure 3B

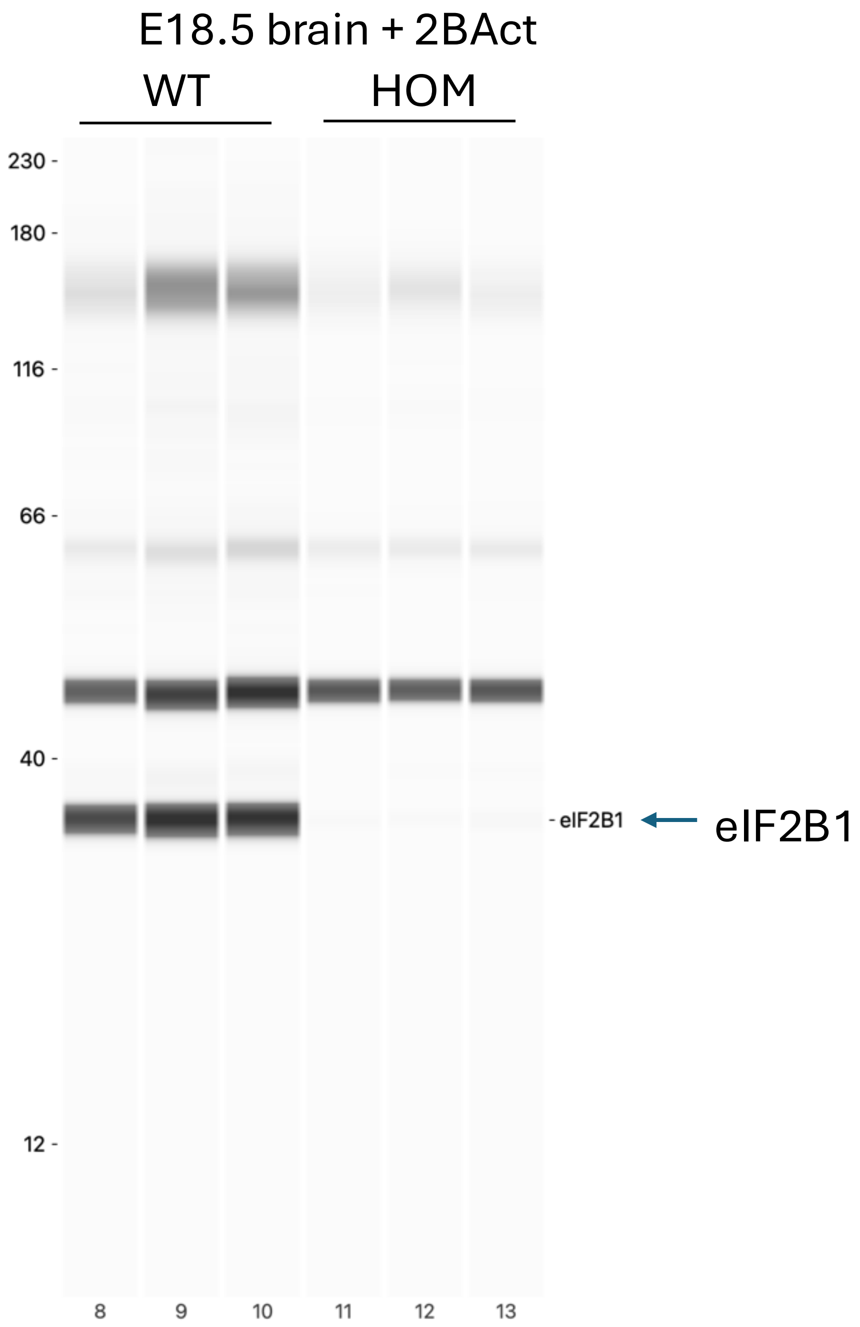

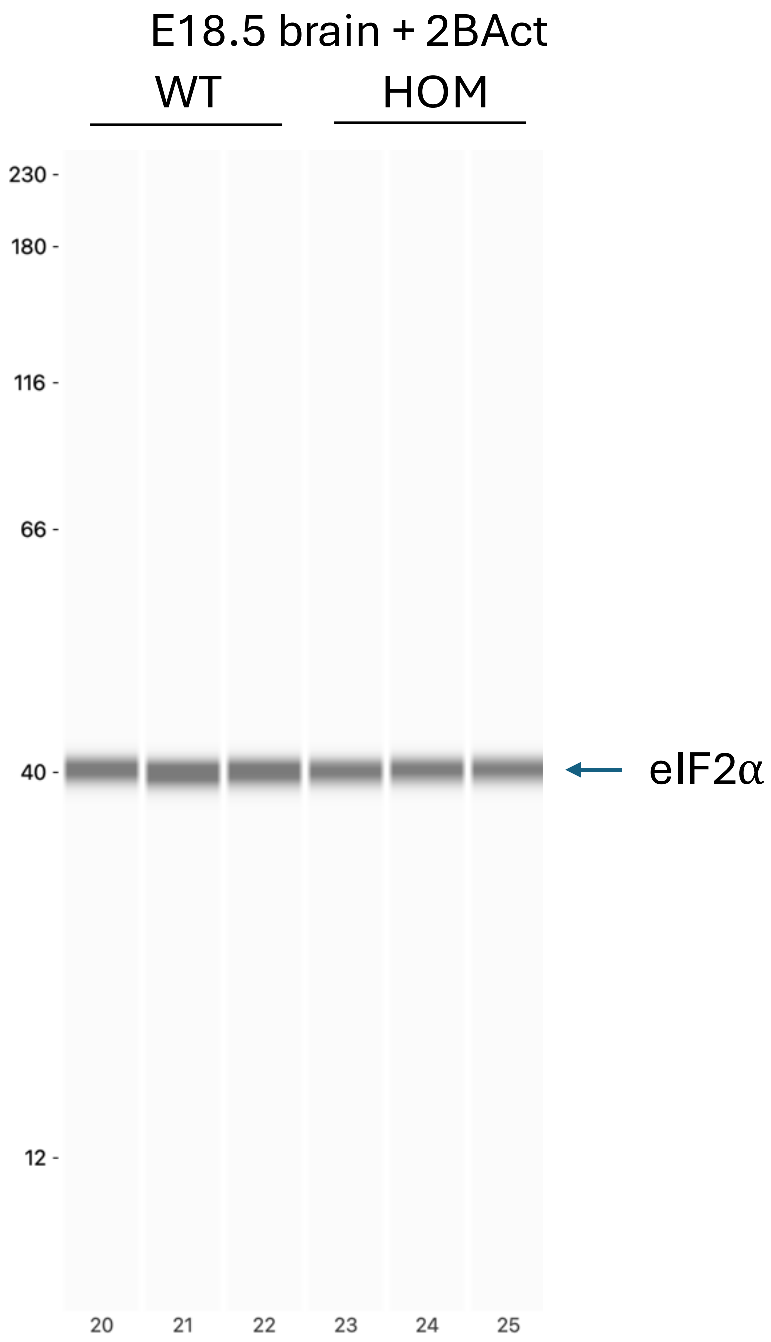

Full unedited blot for Supplement Figure 3C

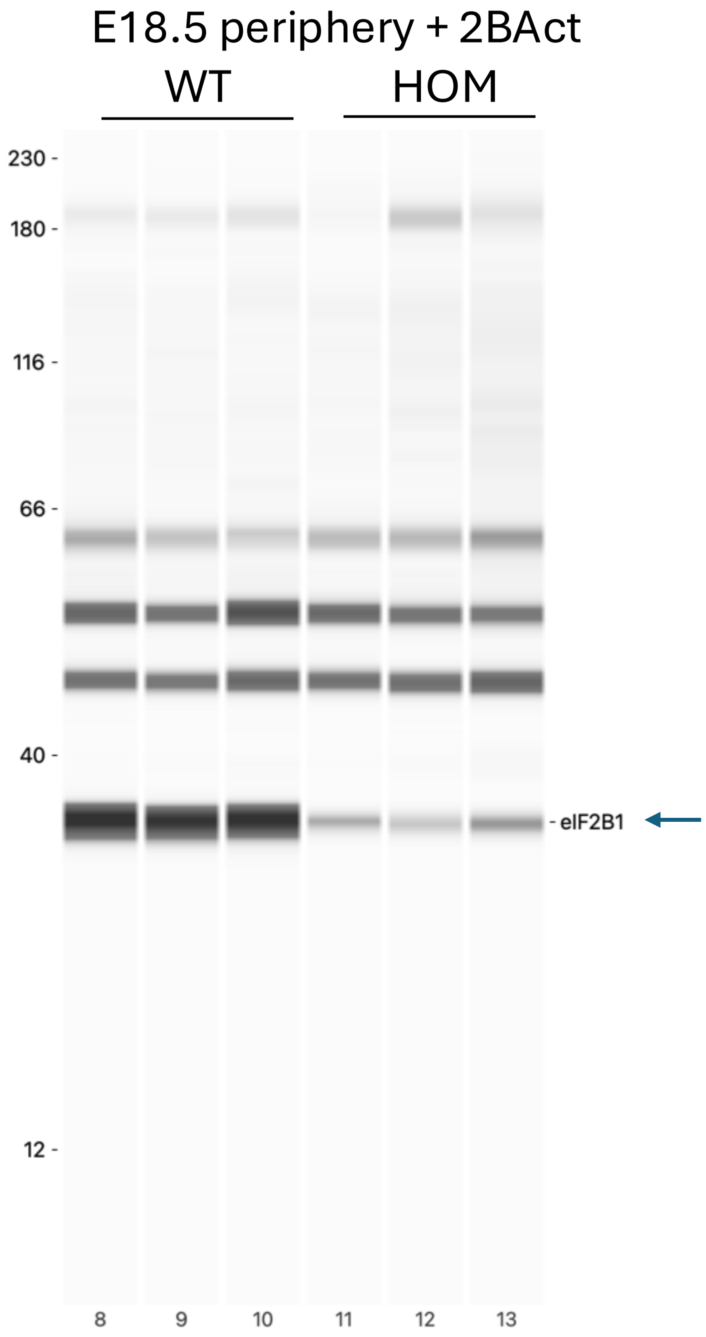

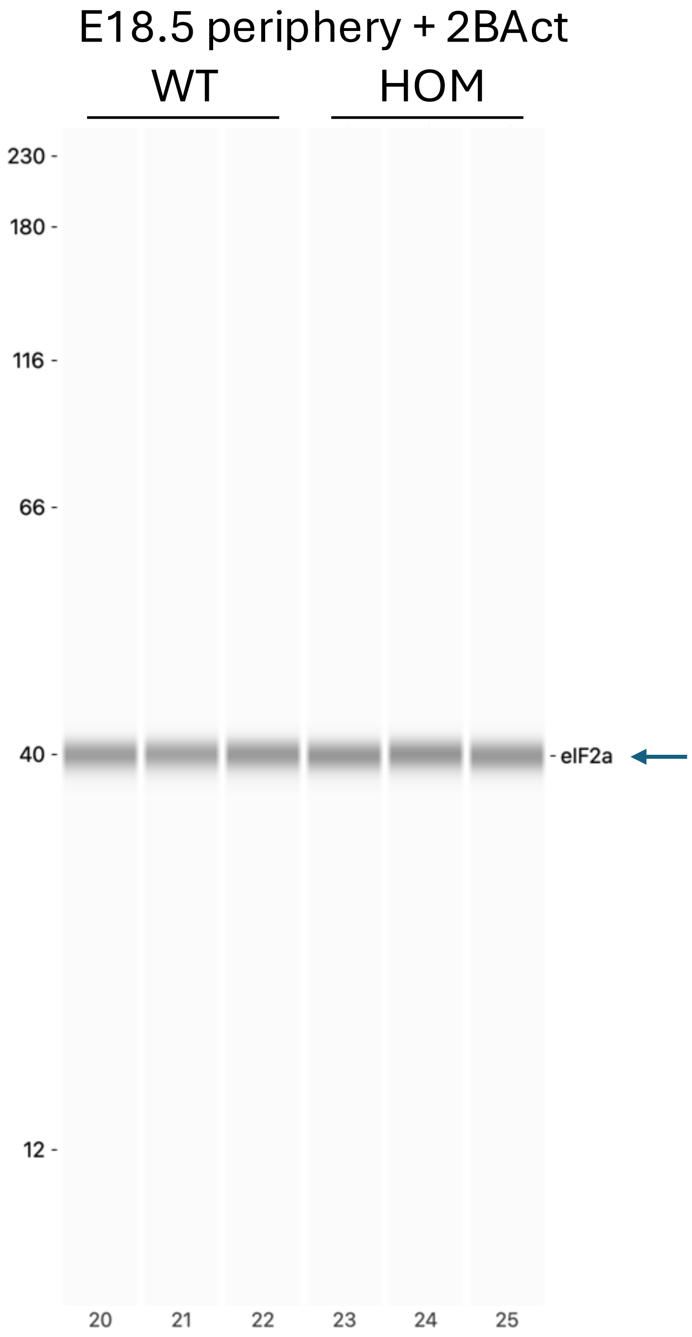

Full unedited blot for Supplement Figure 4B

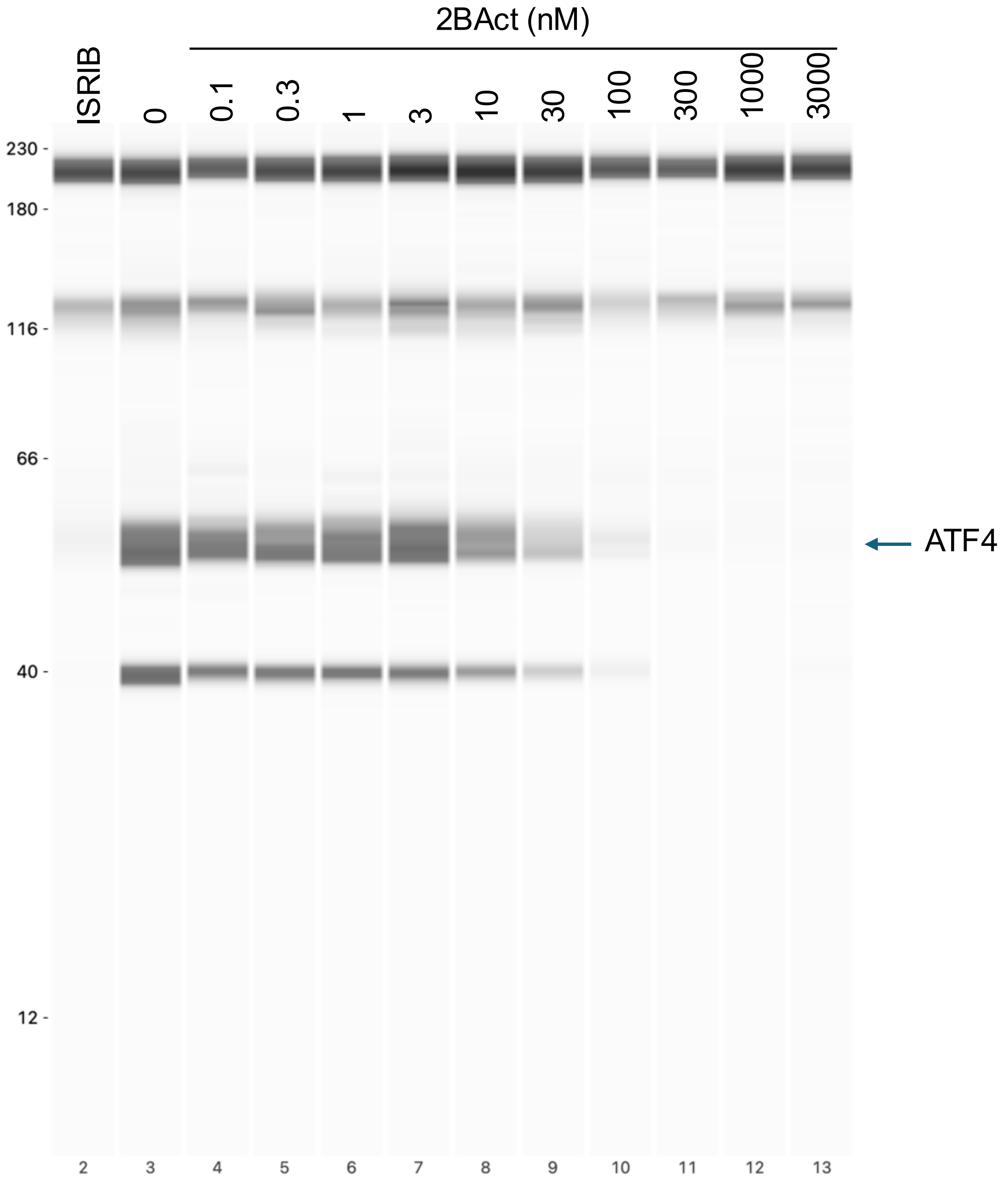

Full unedited blot for
Supplement Figure 4B

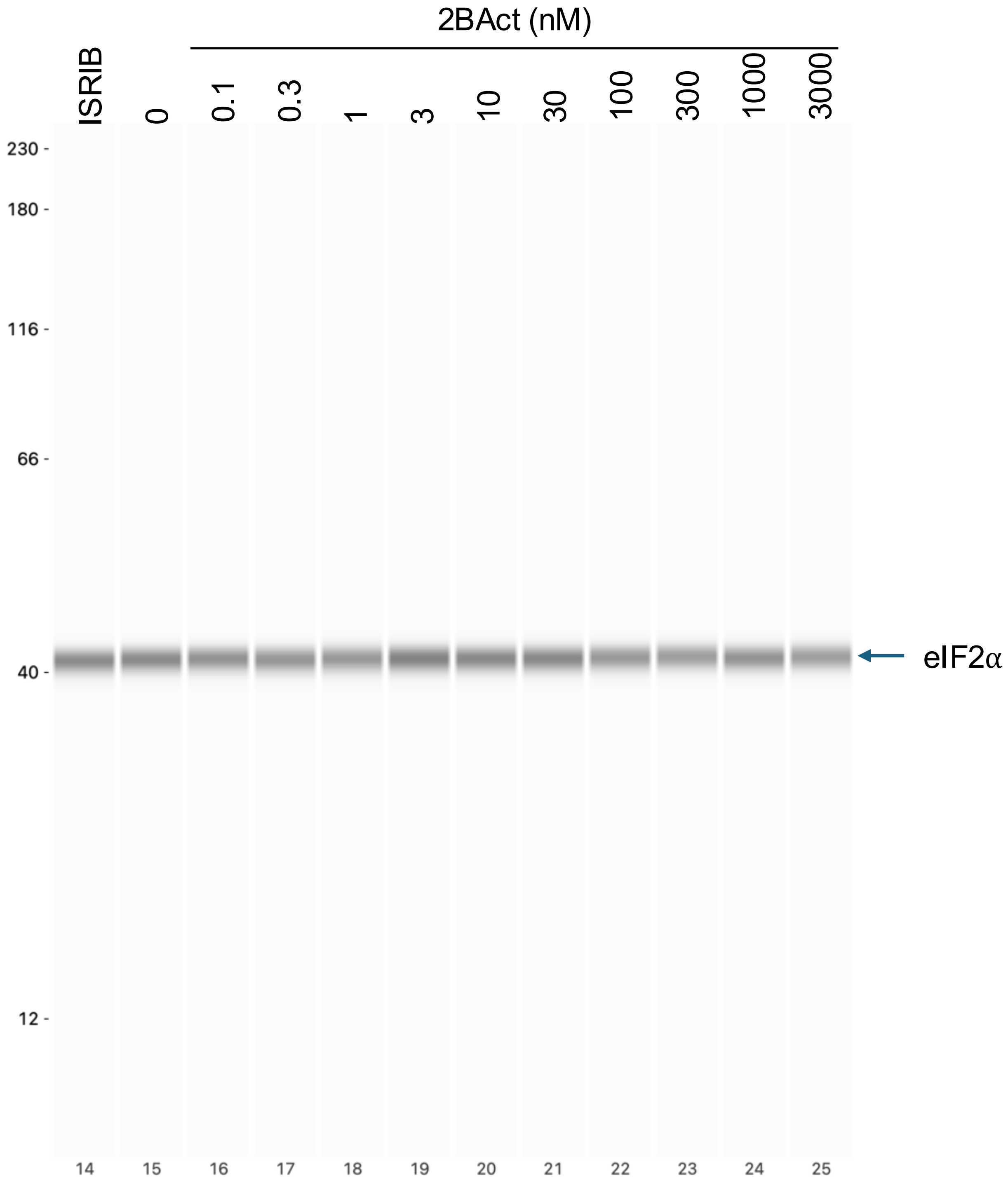

Full unedited blot for Supplement Figure 4D

Brain

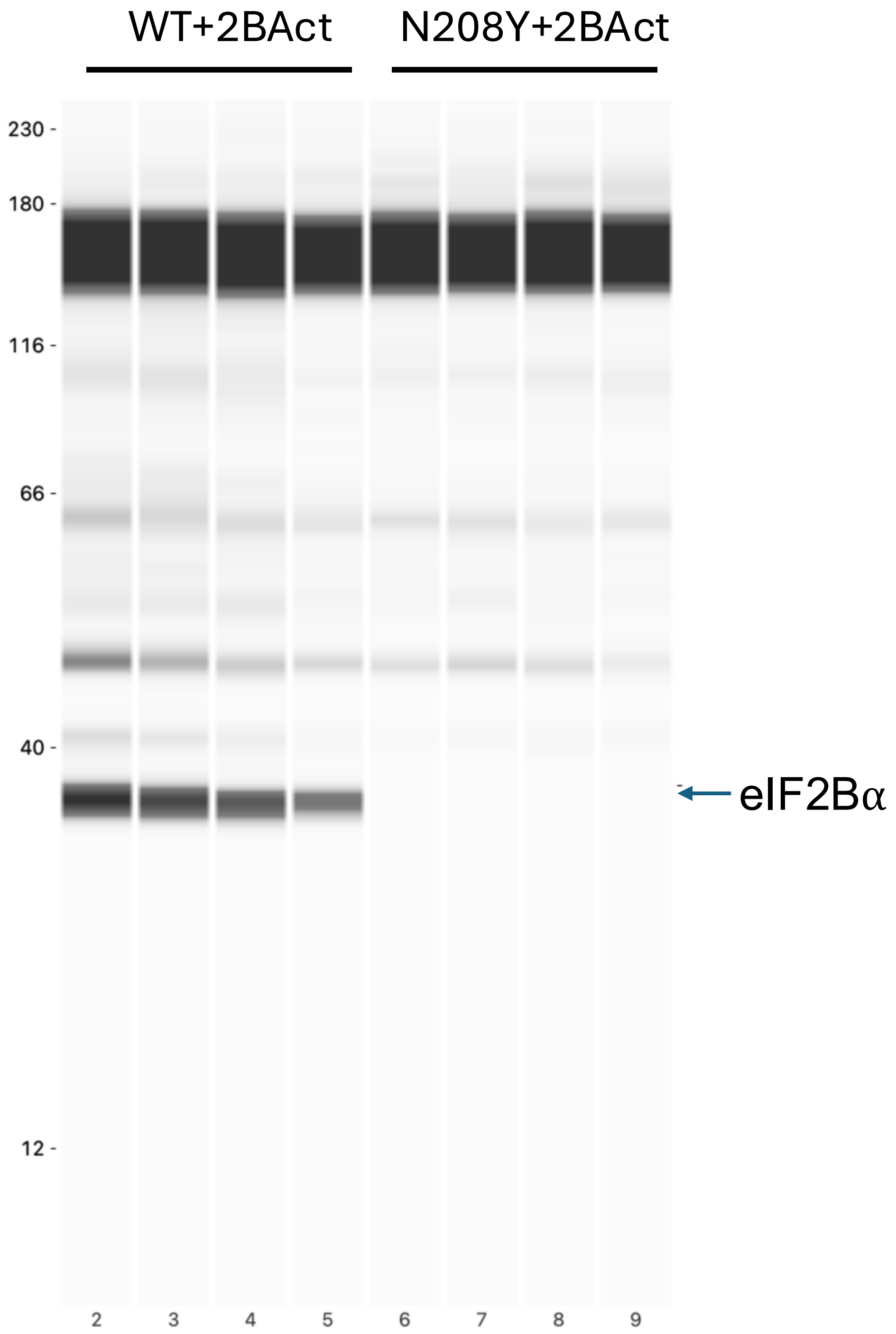

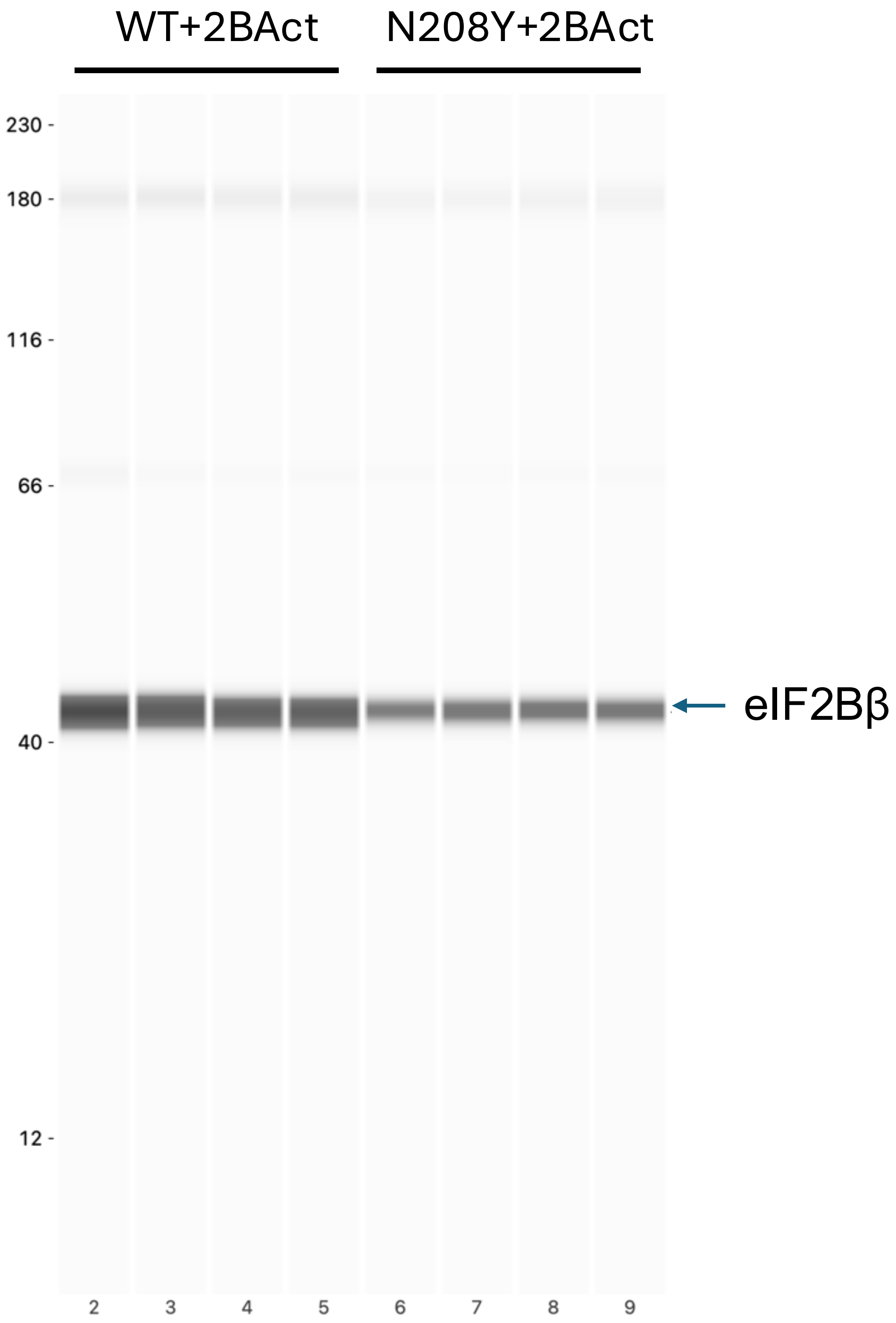

Full unedited blot for Supplement Figure 4D

Brain

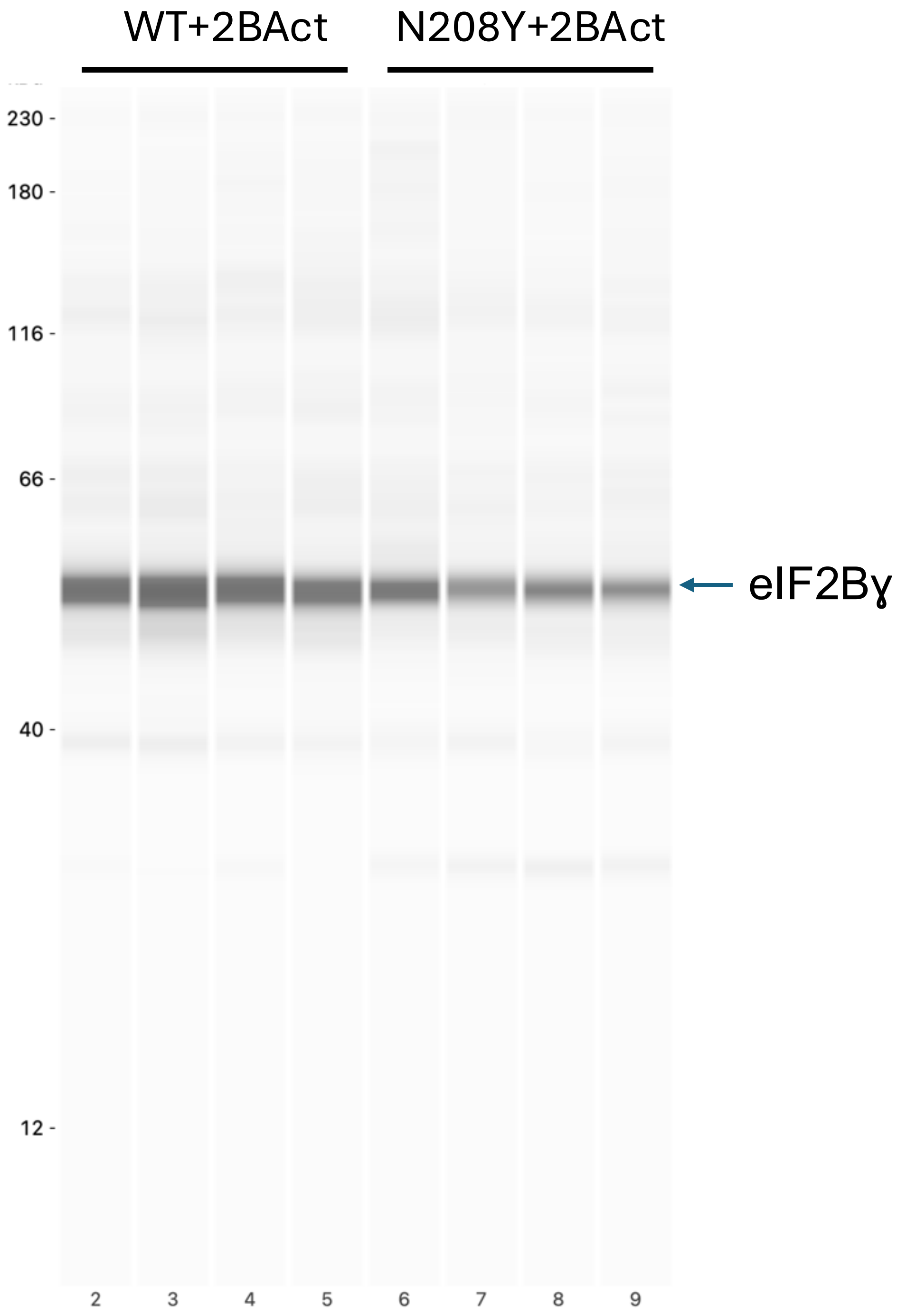

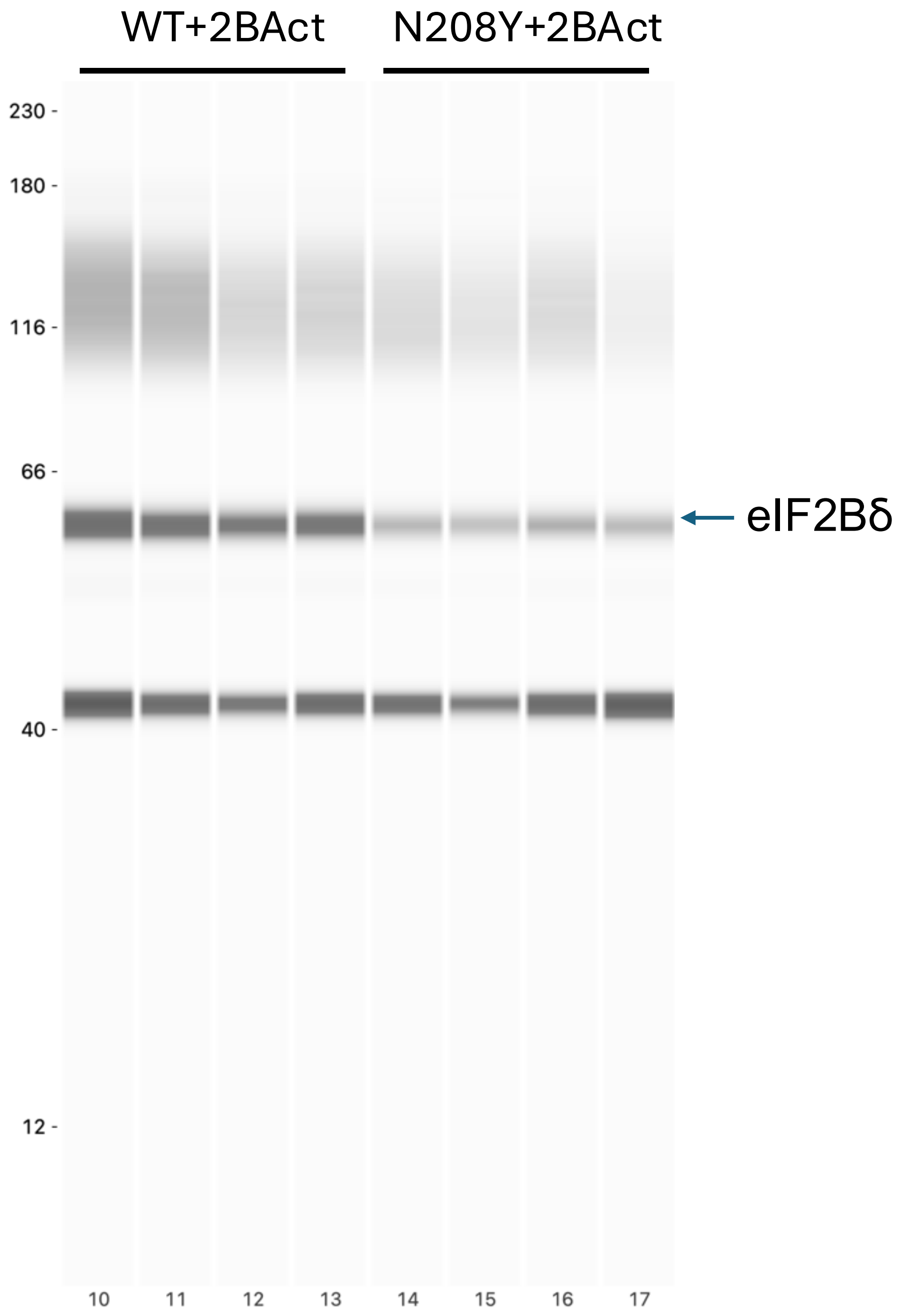

Full unedited blot for Supplement Figure 4D

Brain

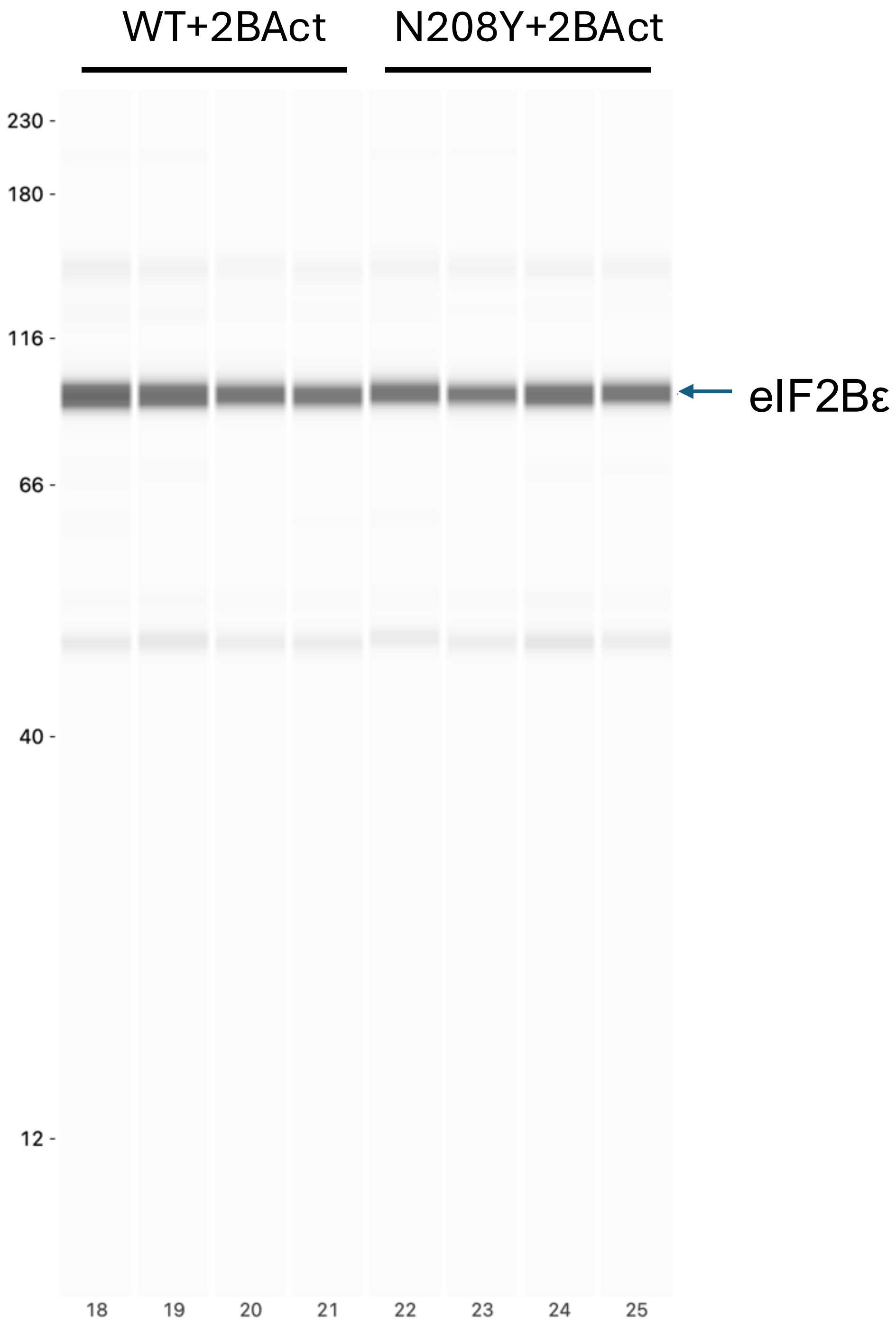

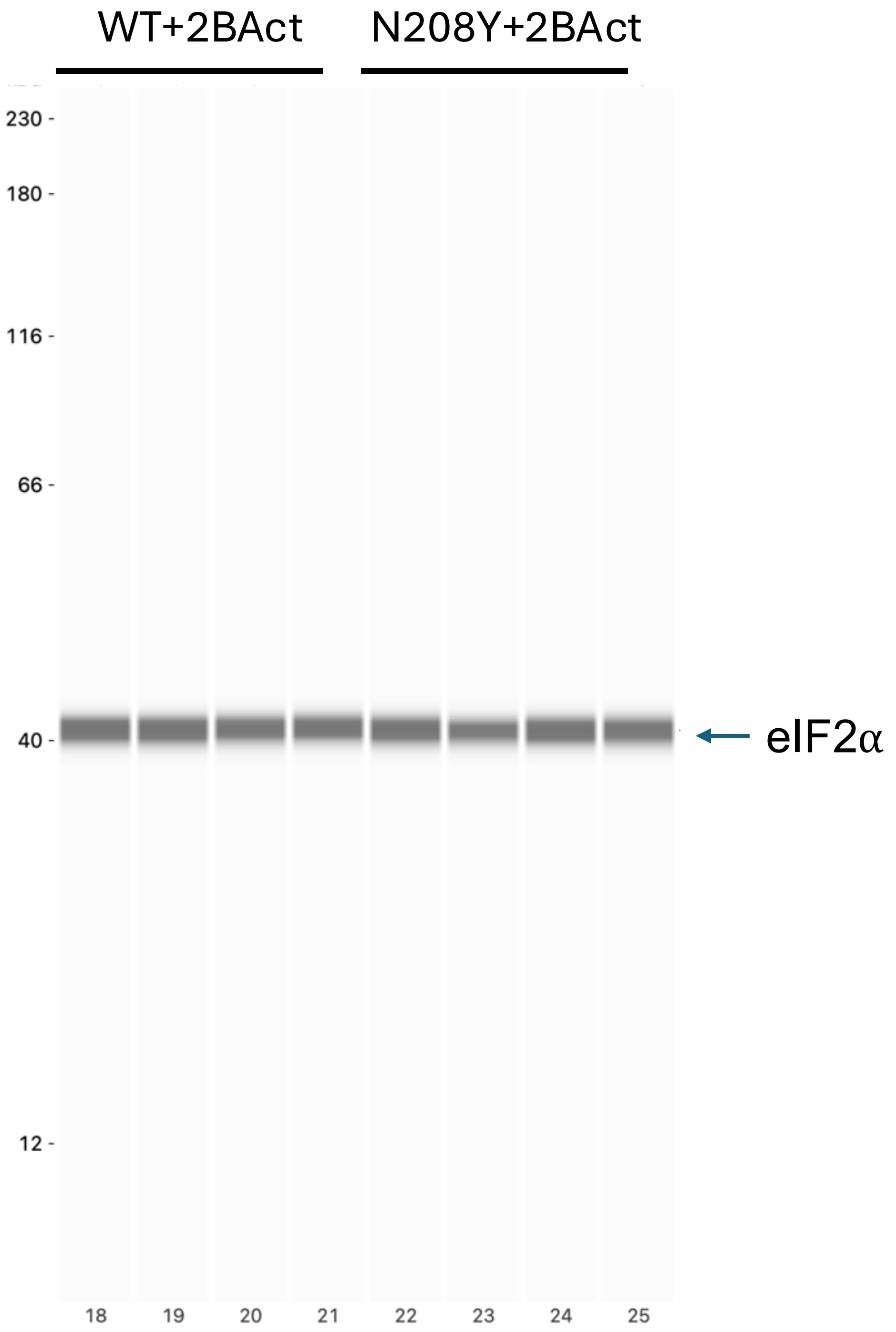

Full unedited blot for Supplement Figure 4D

Liver

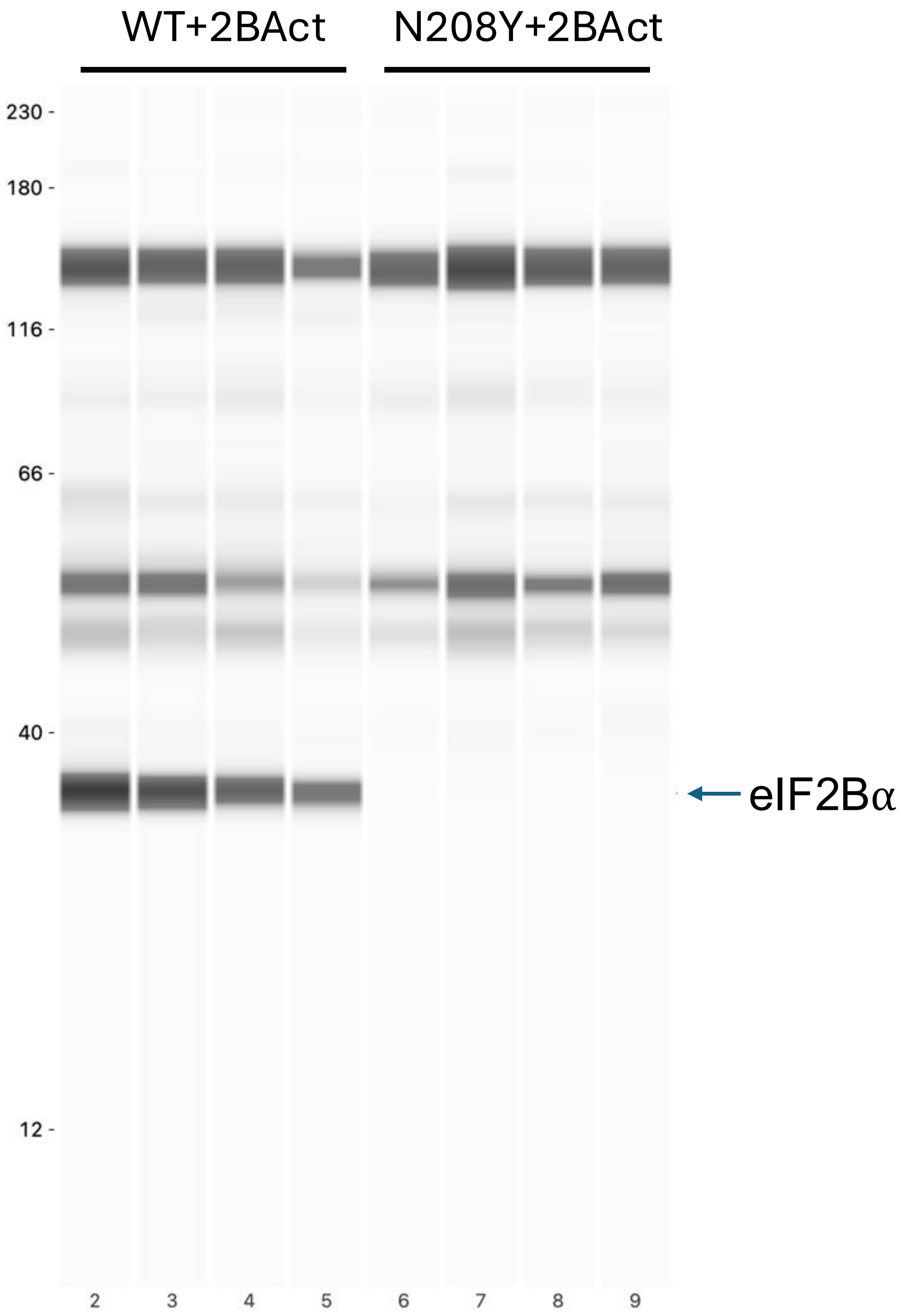

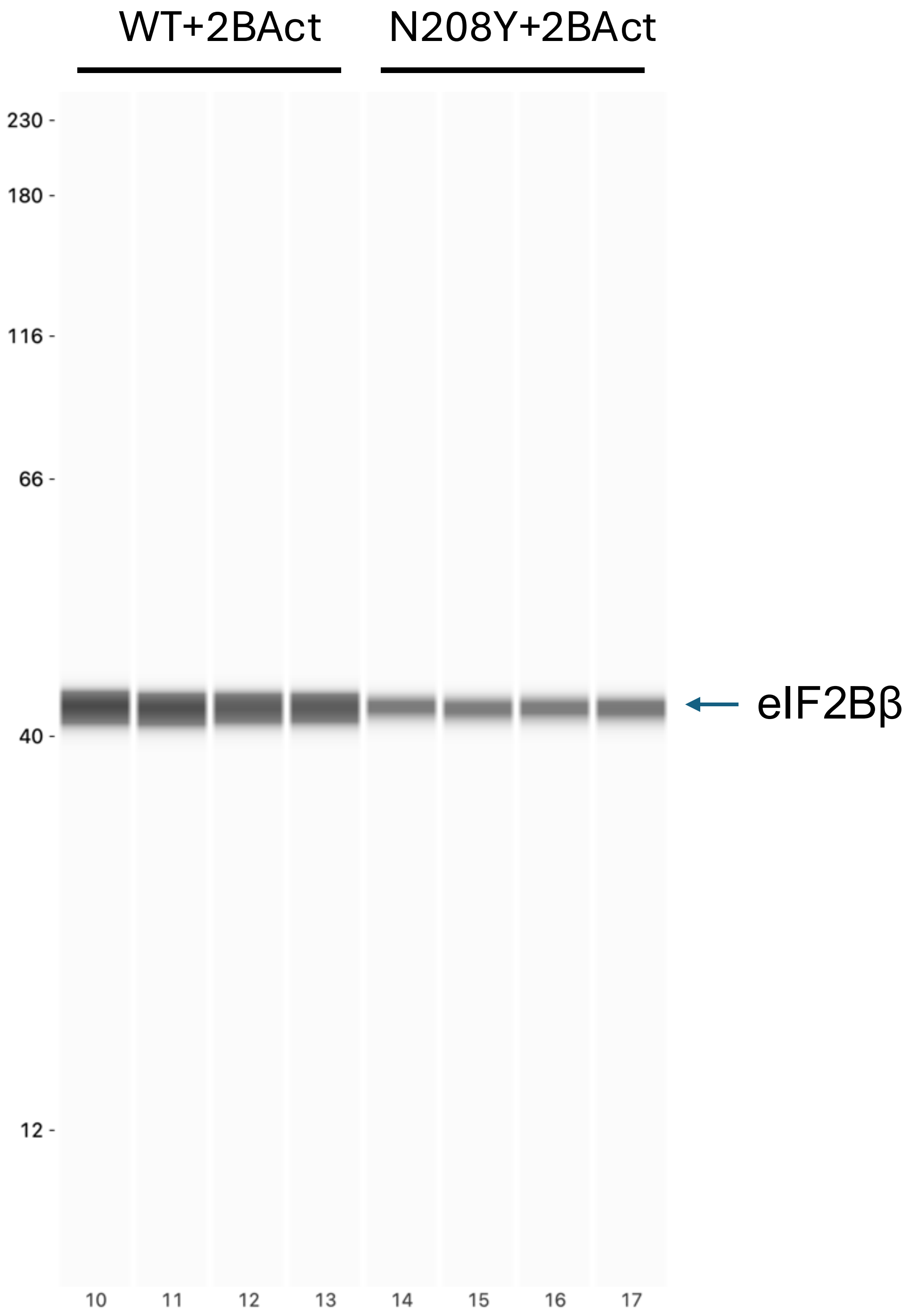

Full unedited blot for Supplement Figure 4D

Liver

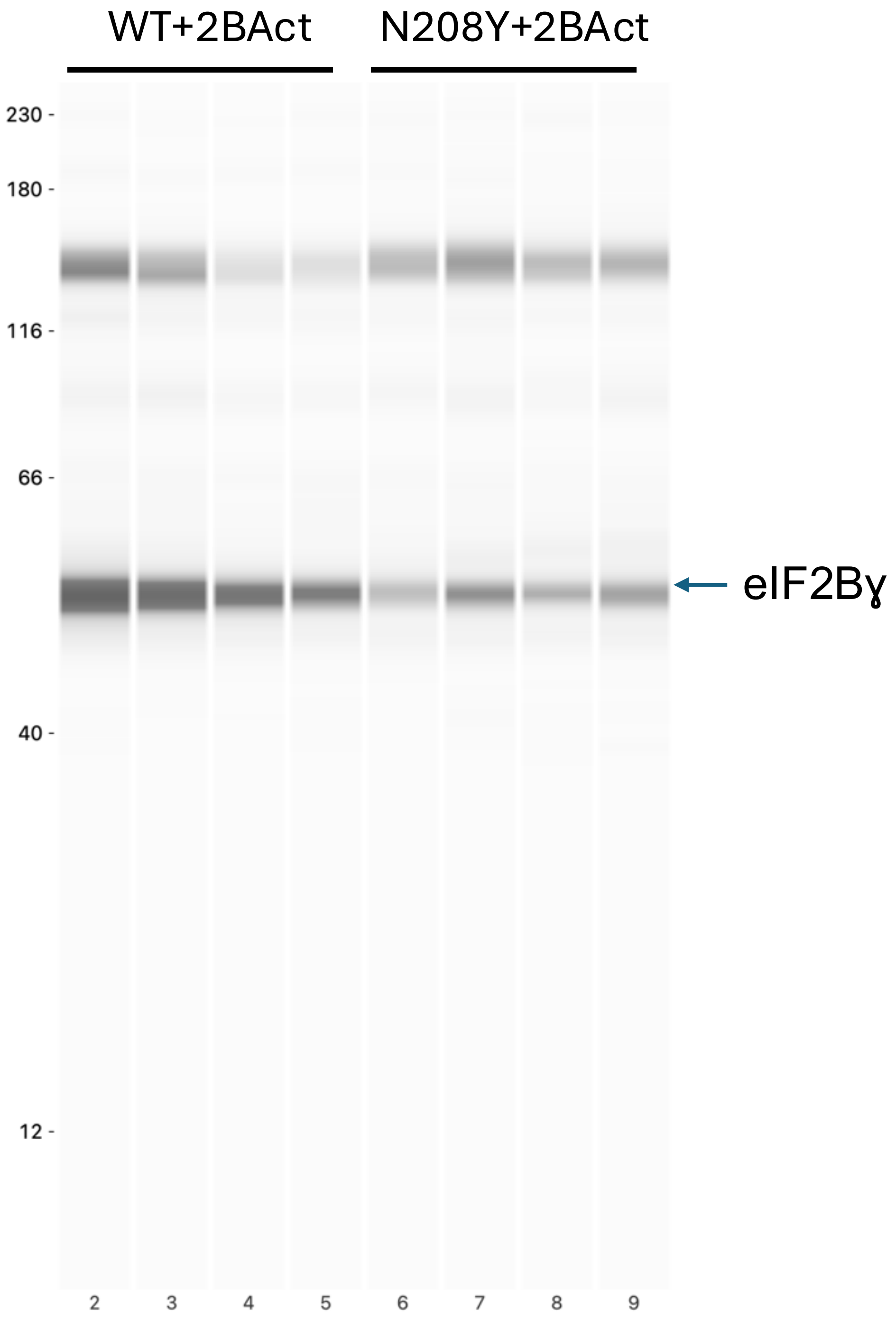

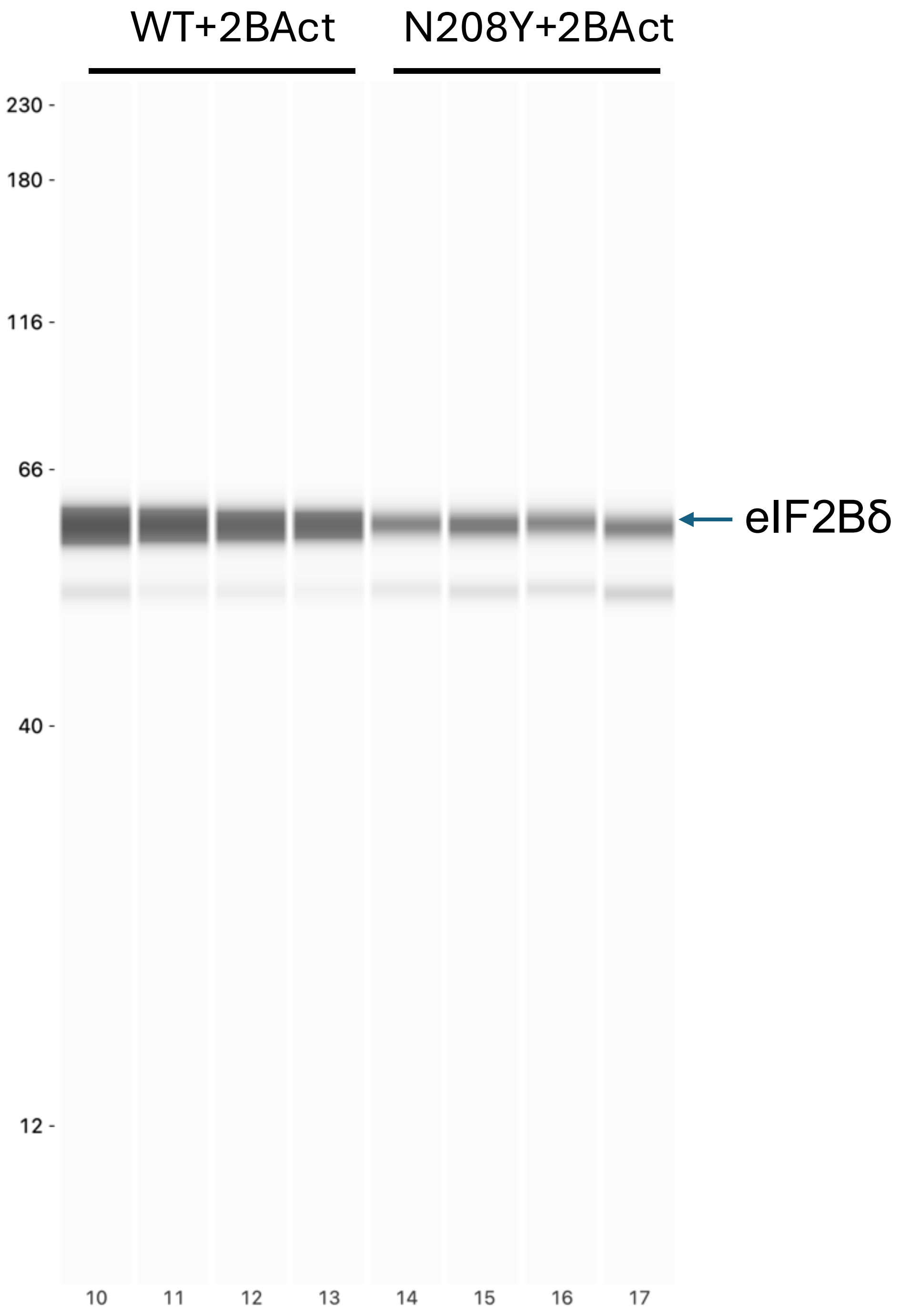

Full unedited blot for Supplement Figure 4D

Liver

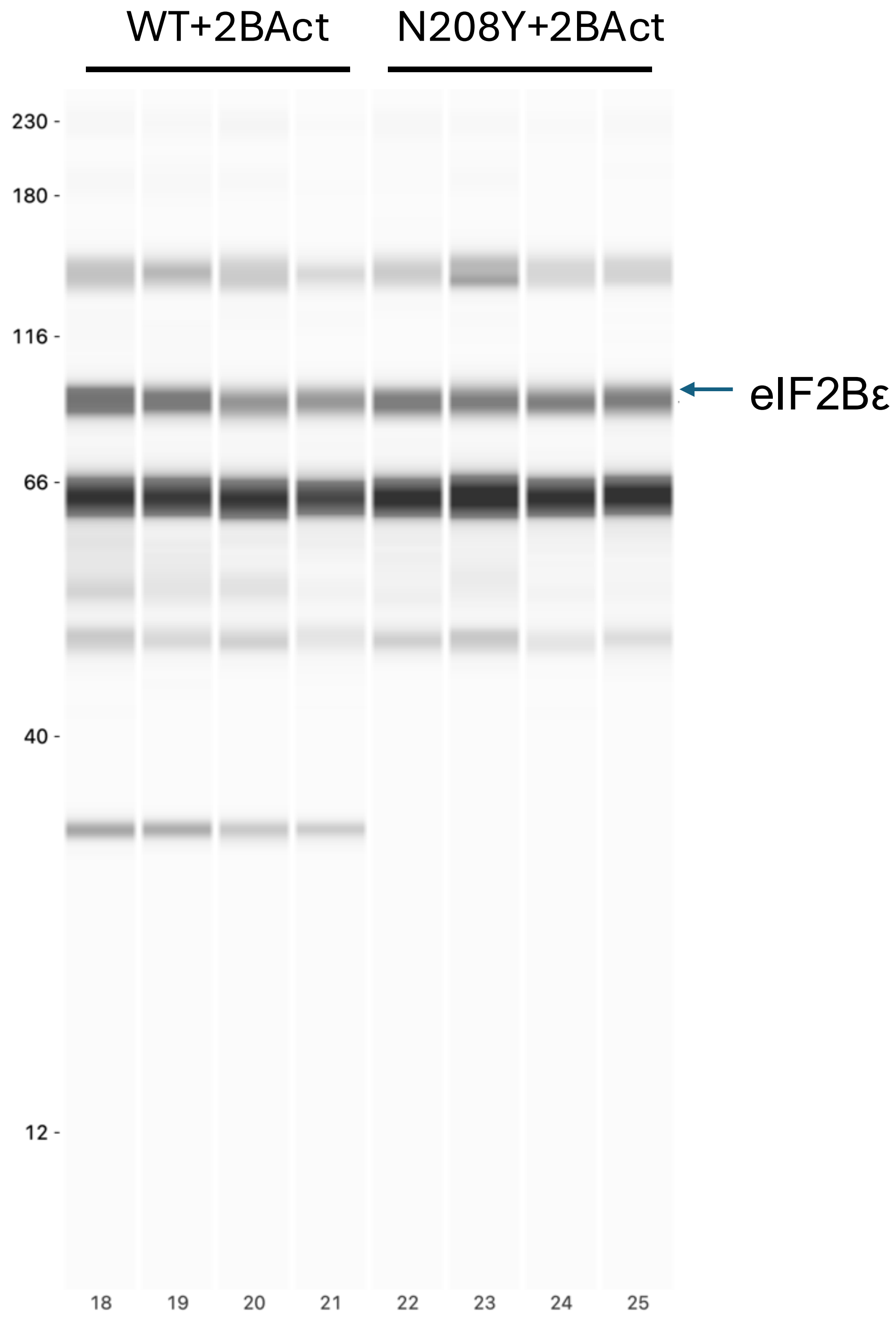

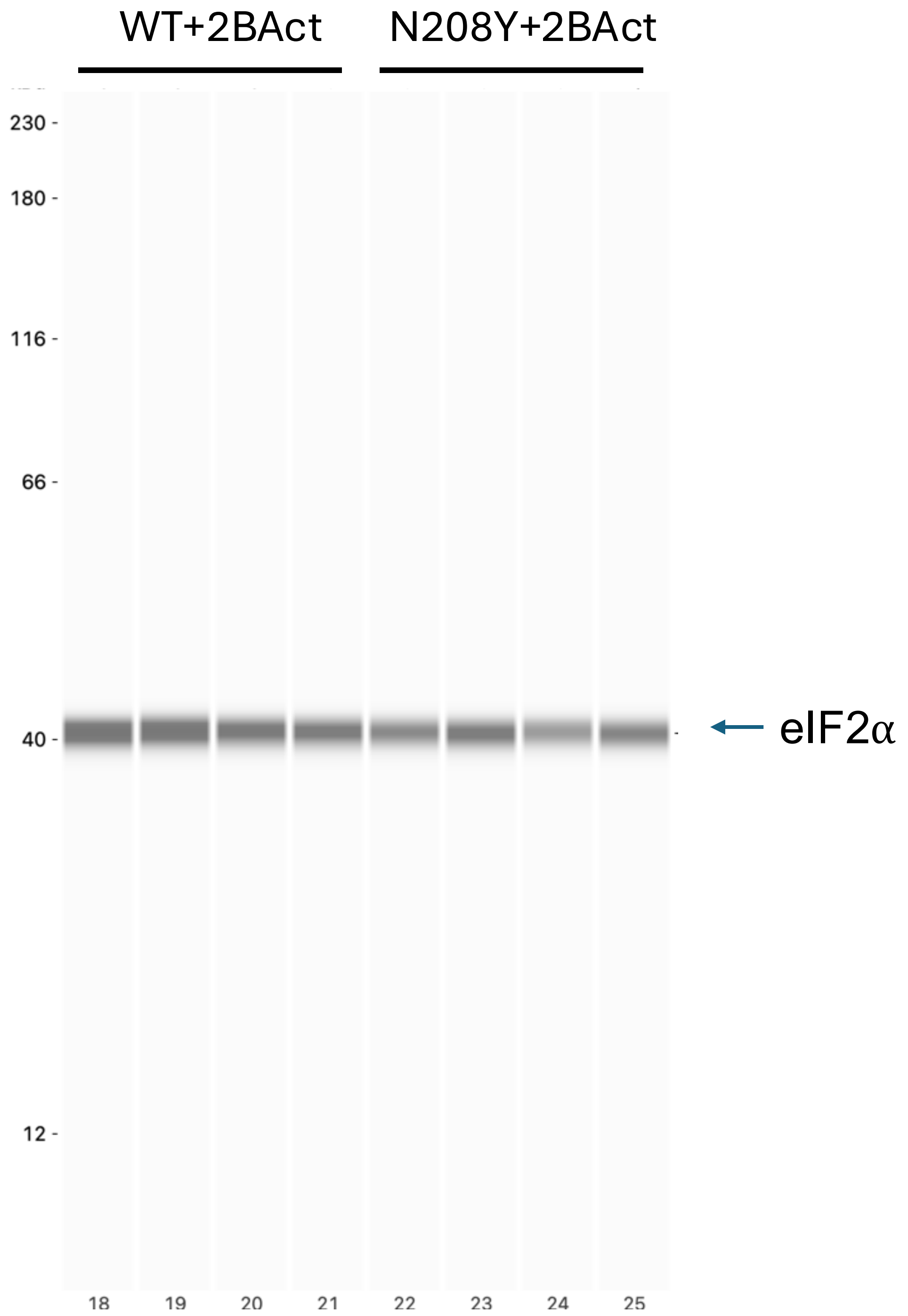

Full unedited blot for Supplement Figure 4D

Kidney

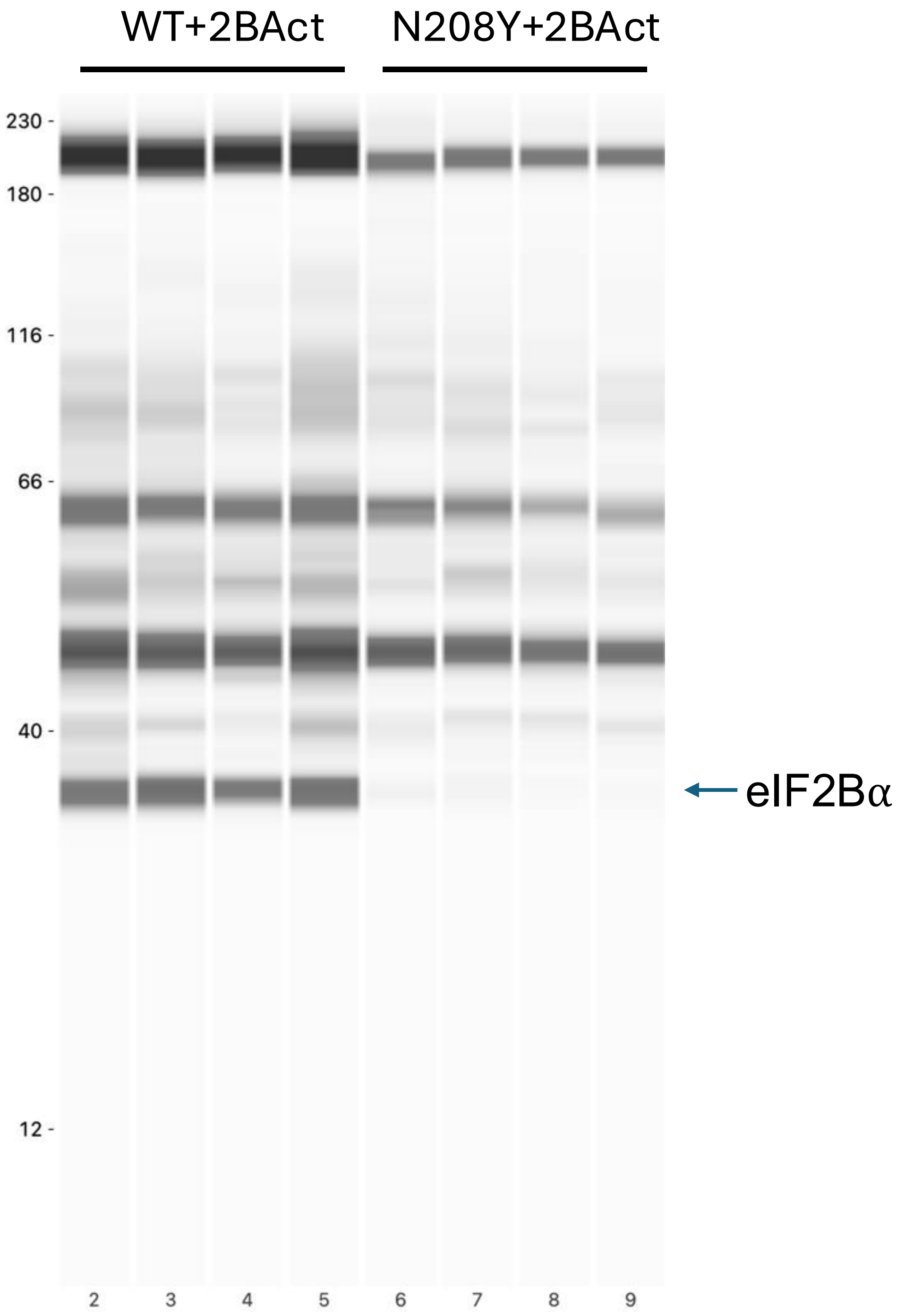

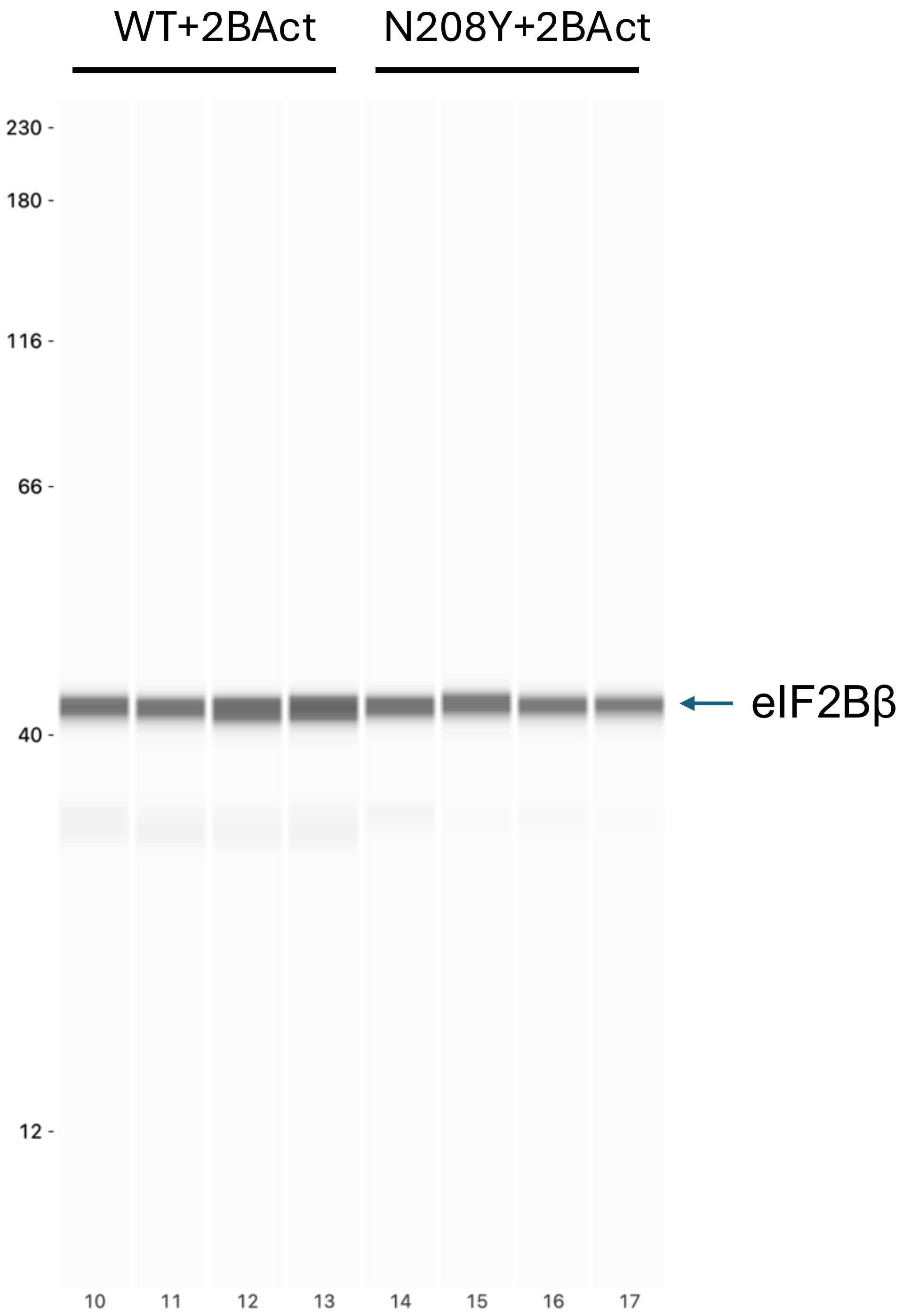

Full unedited blot for Supplement Figure 4D

Kidney

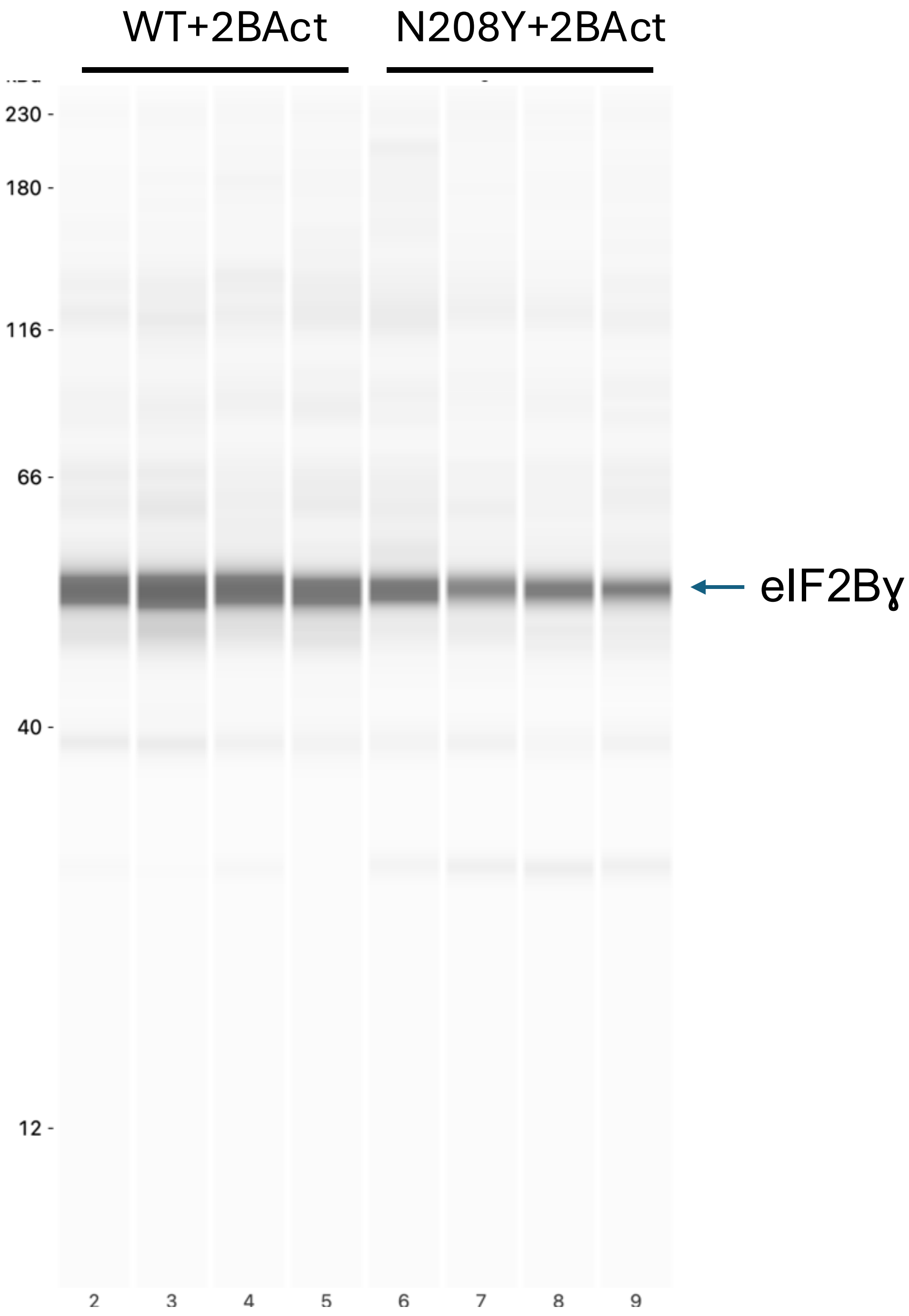

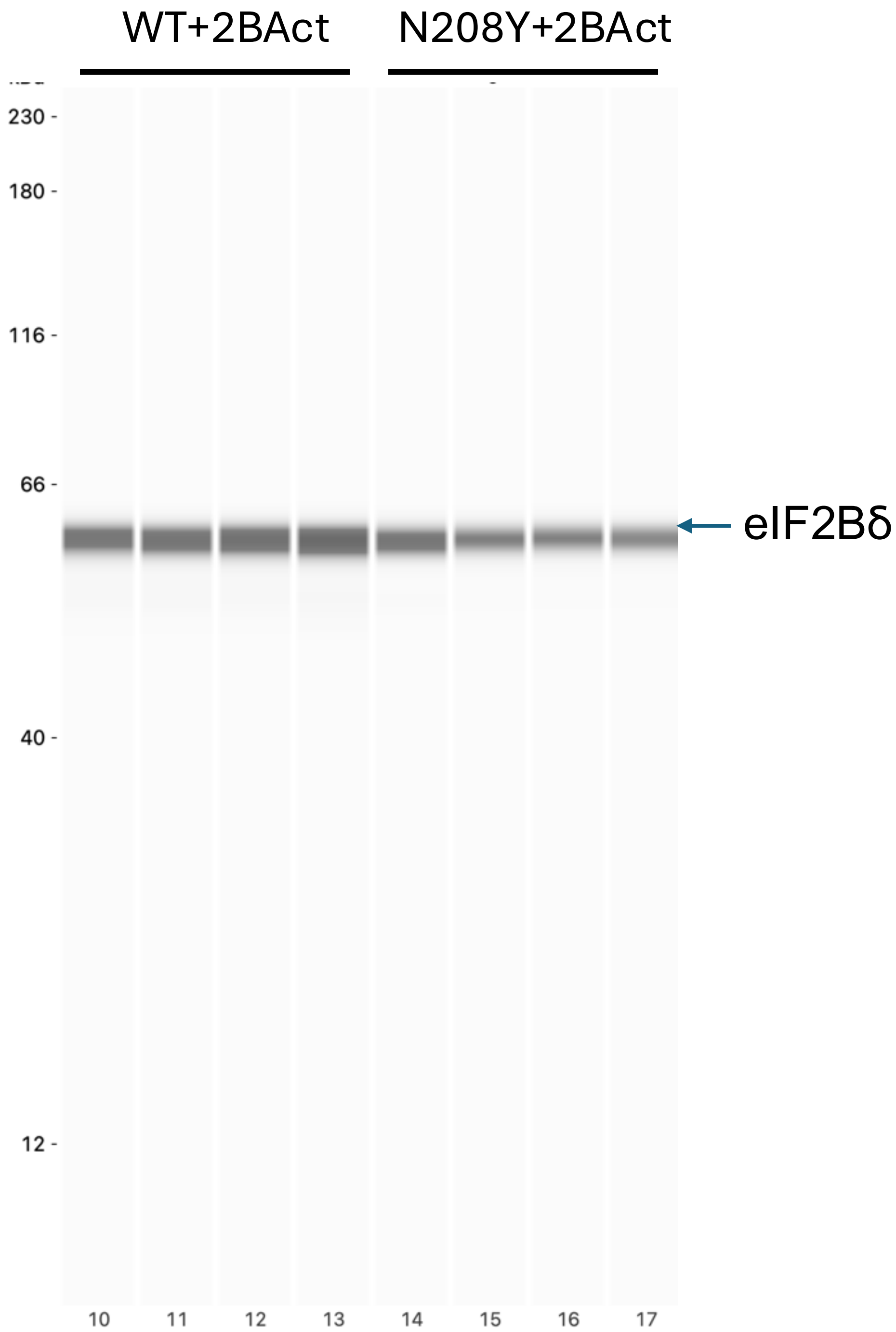

Full unedited blot for Supplement Figure 4D

Kidney

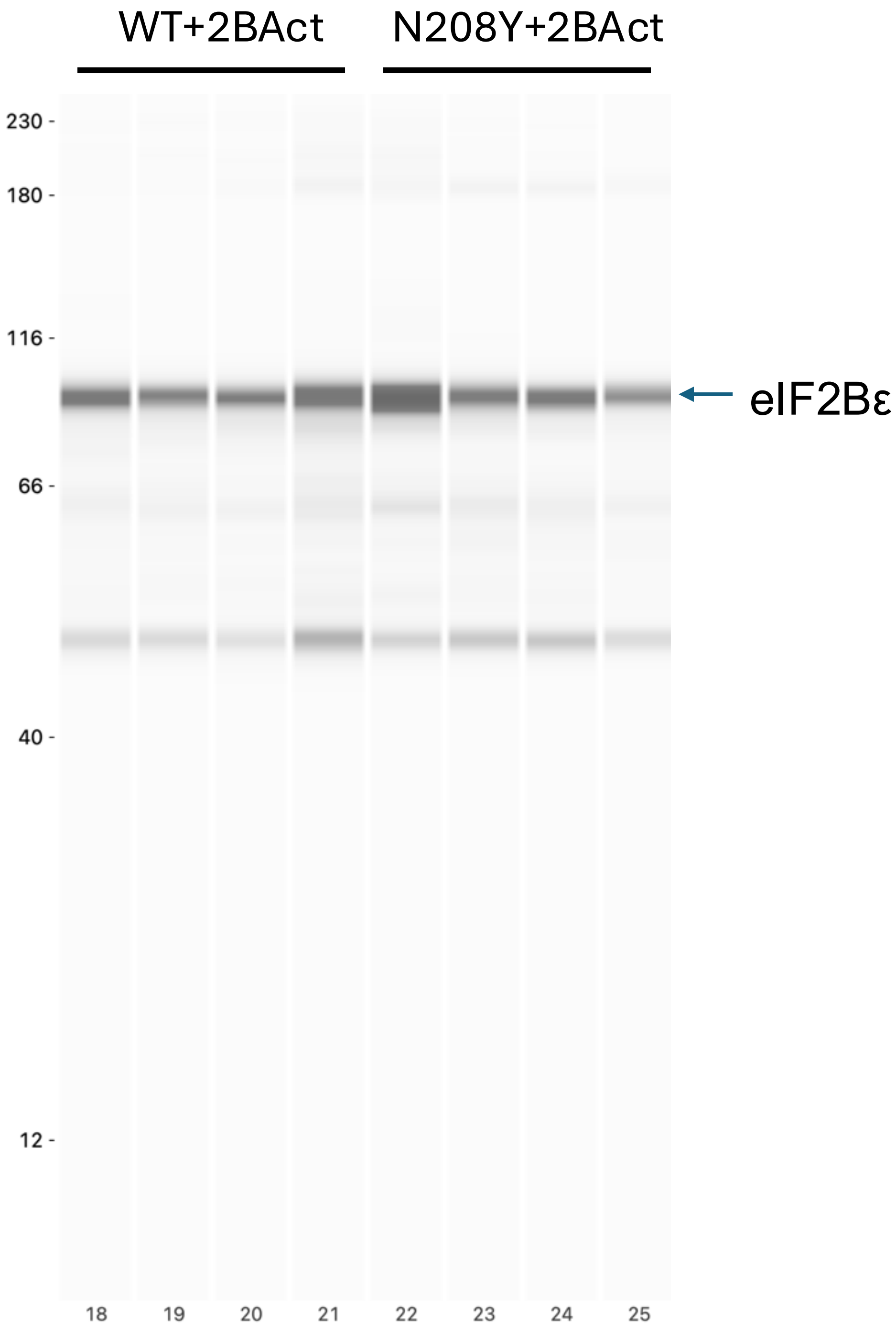

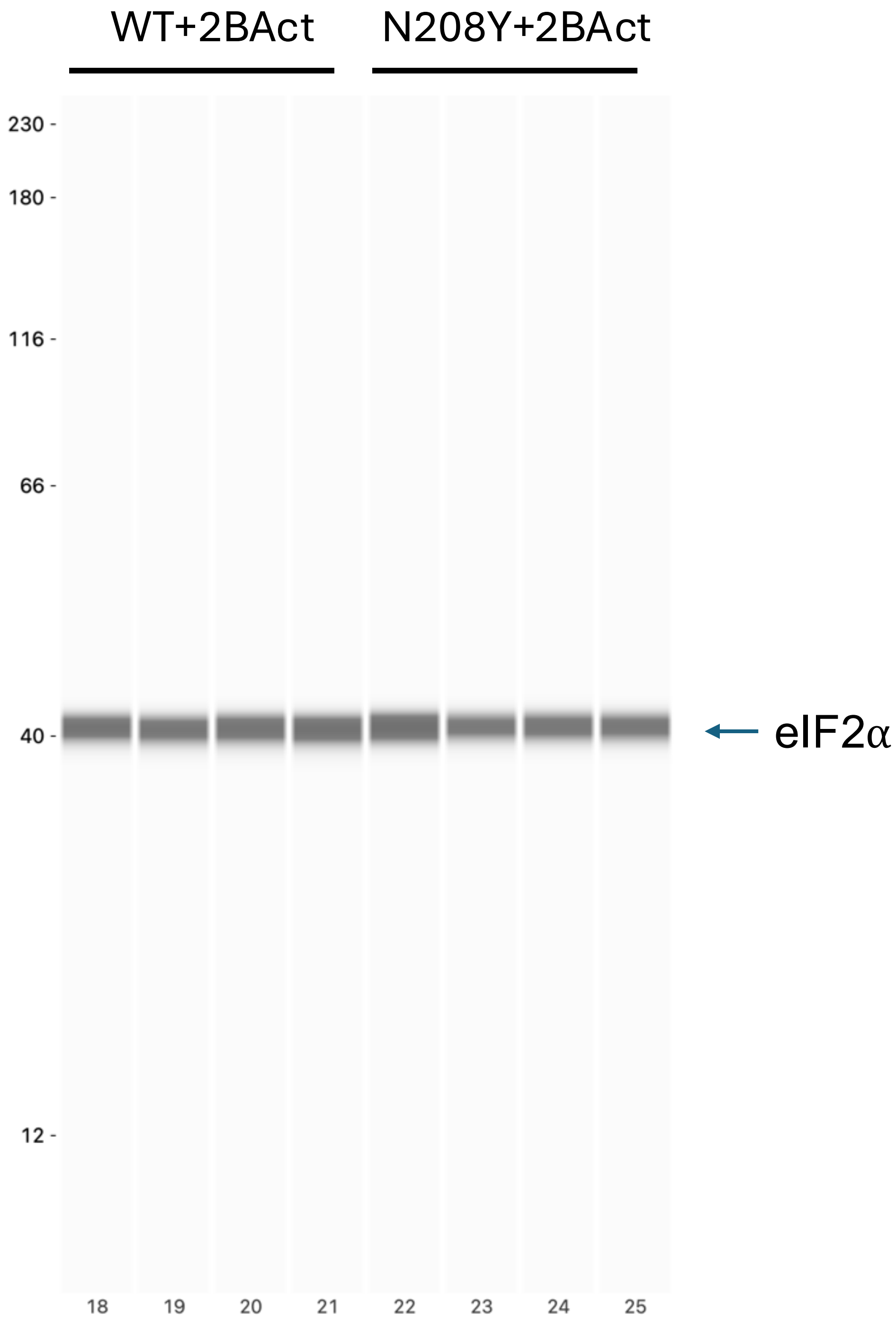

Full unedited blot for Supplement Figure 4D

Lung

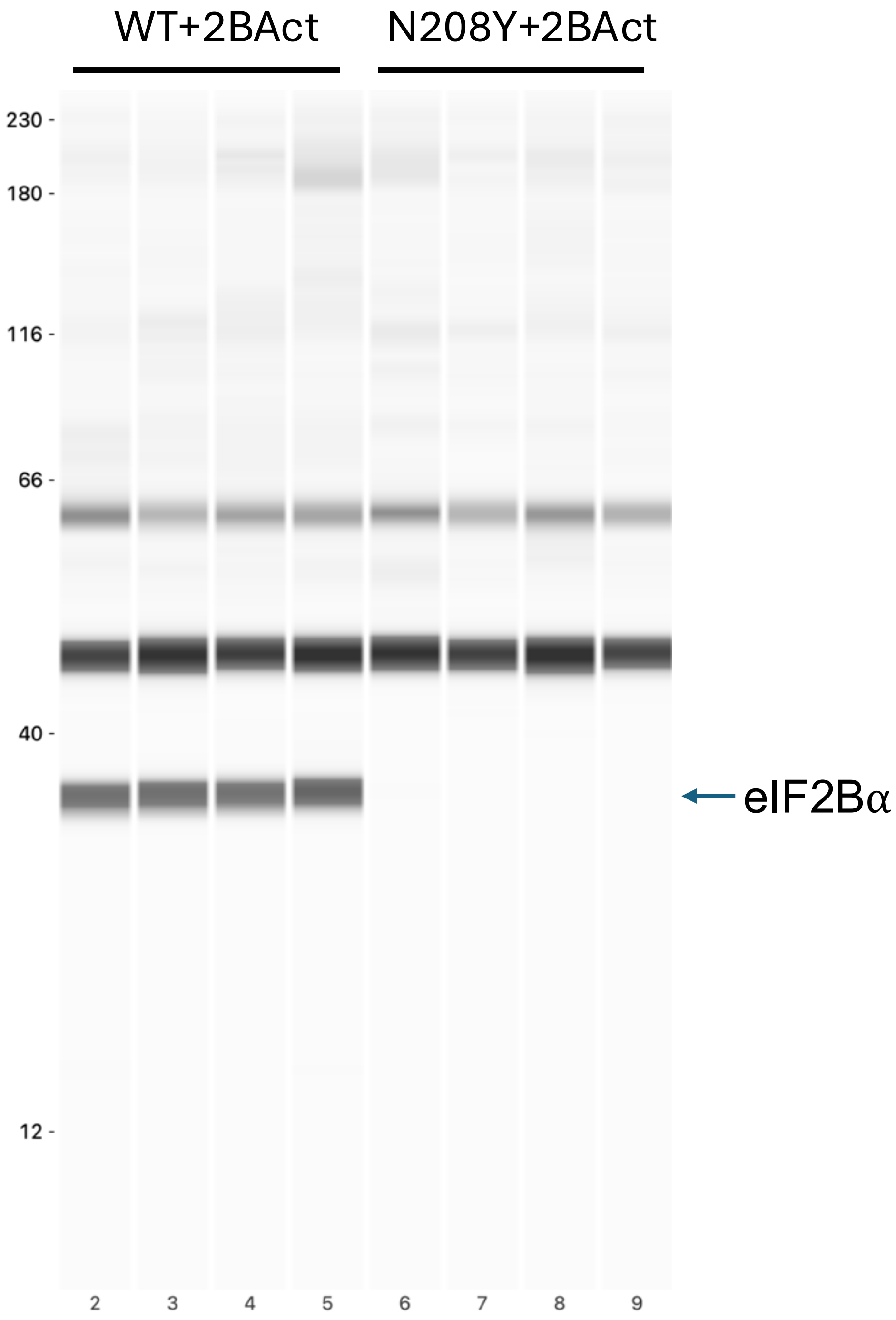

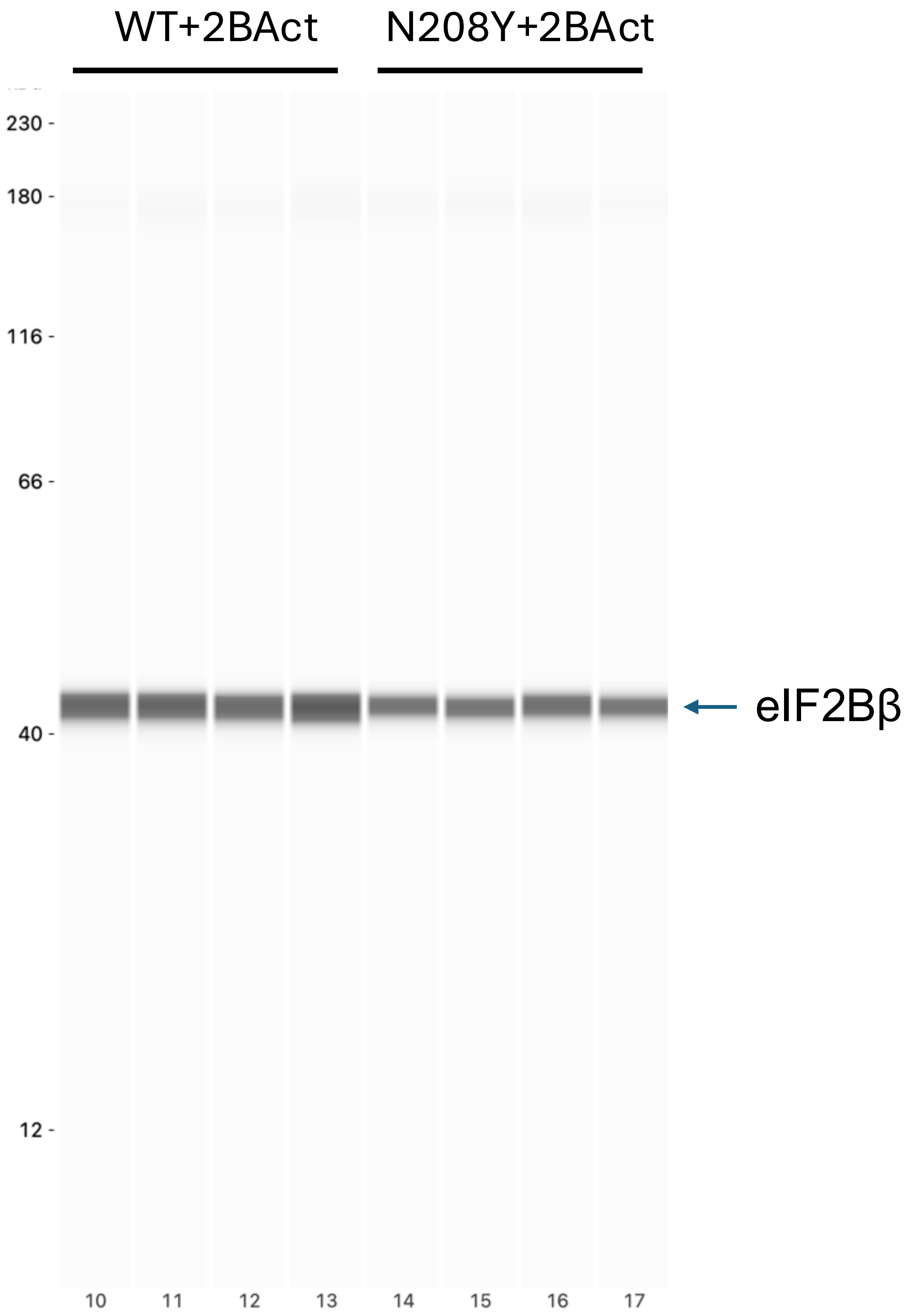

Full unedited blot for Supplement Figure 4D

Lung

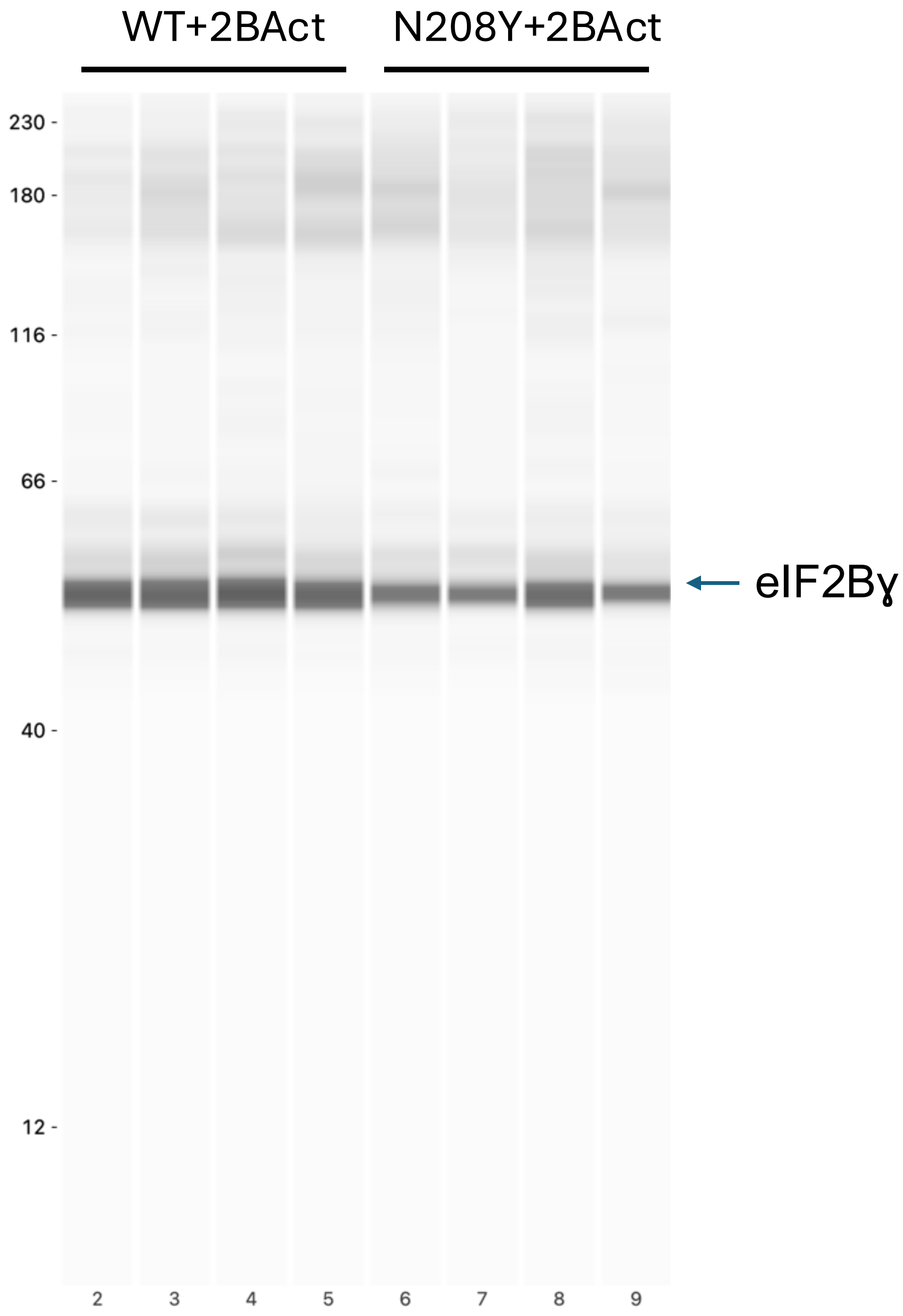

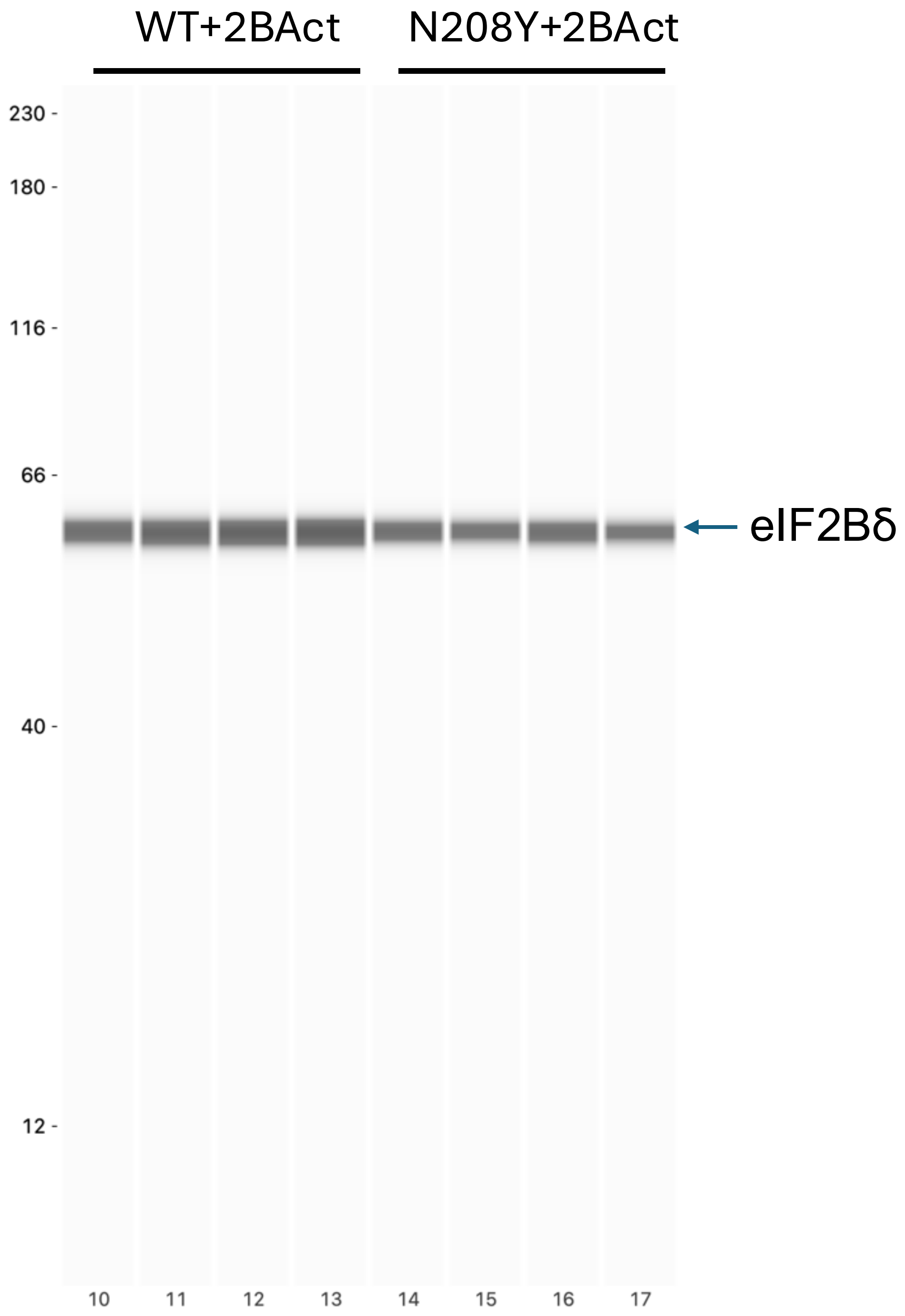

Full unedited blot for Supplement Figure 4D

Lung

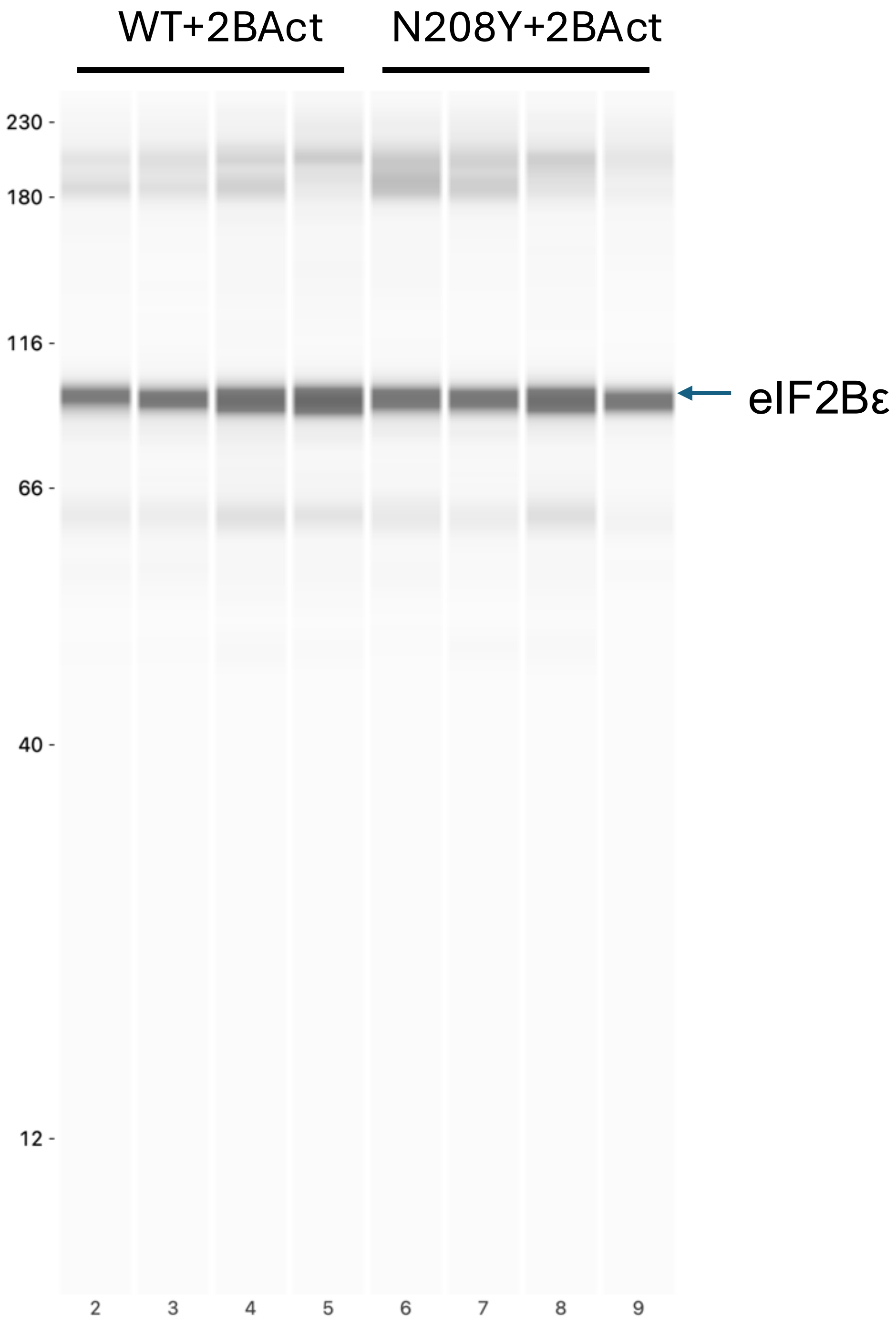

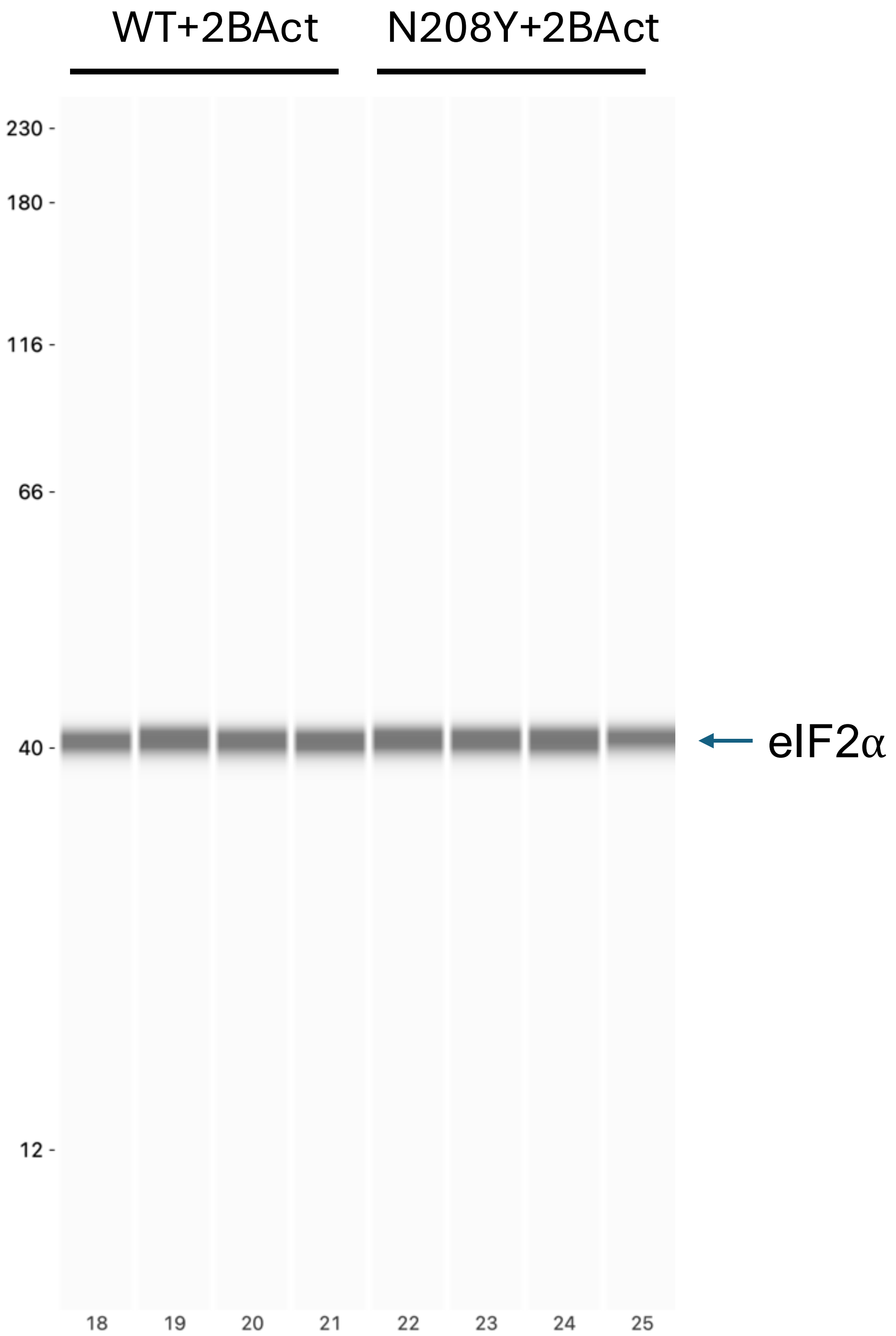

Full unedited blot for Supplement Figure10E

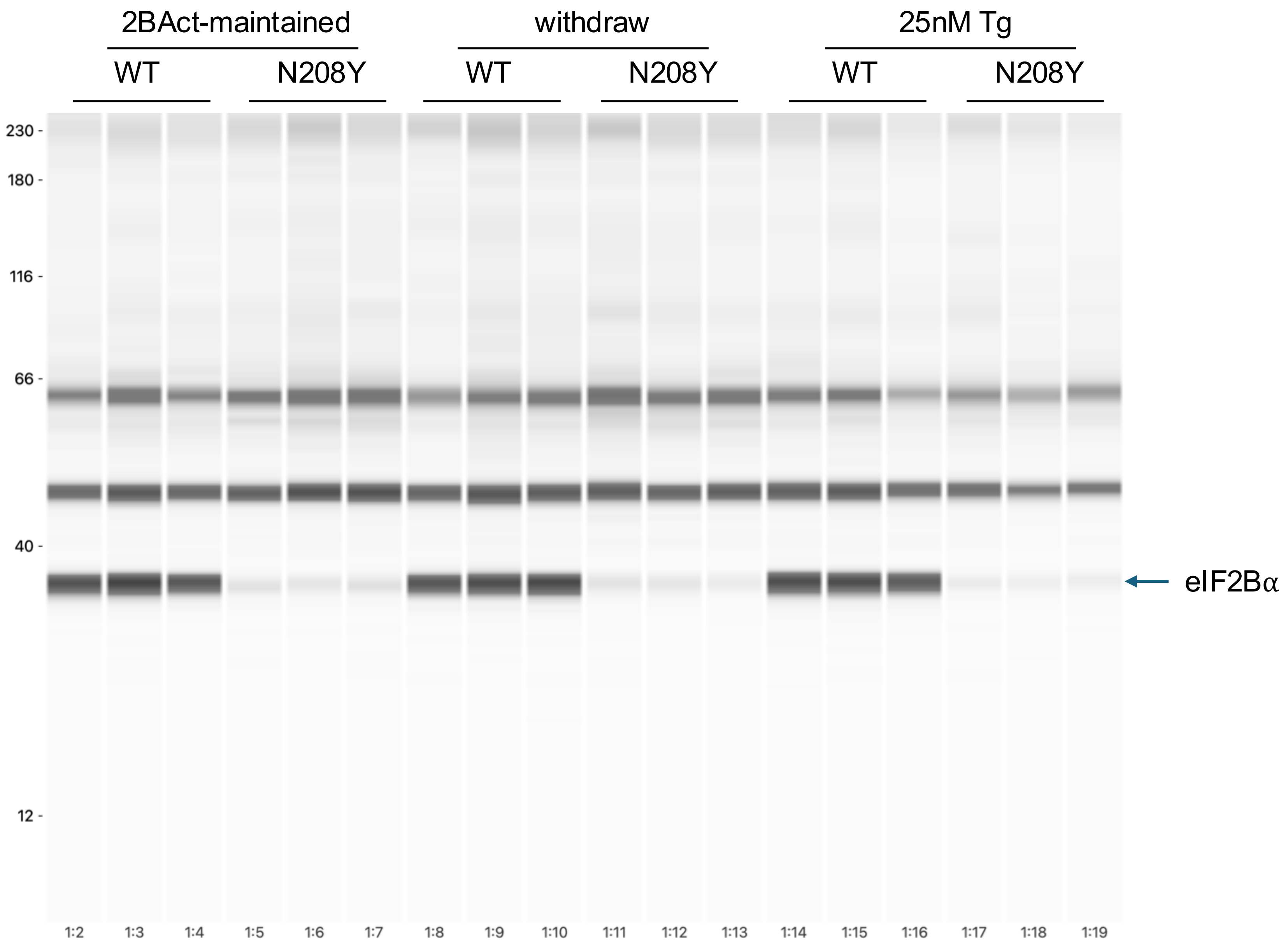

Full unedited blot for Supplement Figure10E

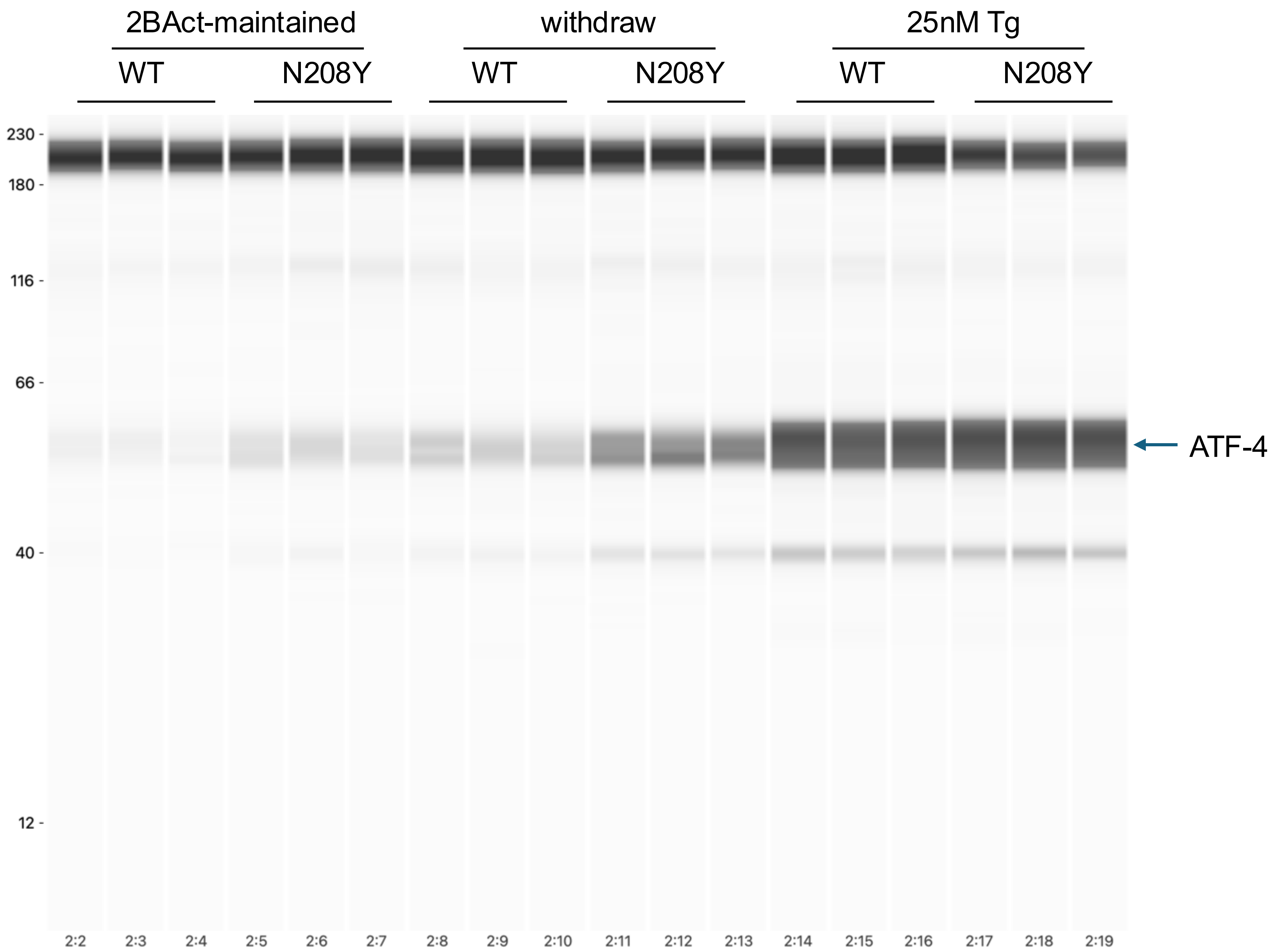

Full unedited blot for Supplement Figure10E

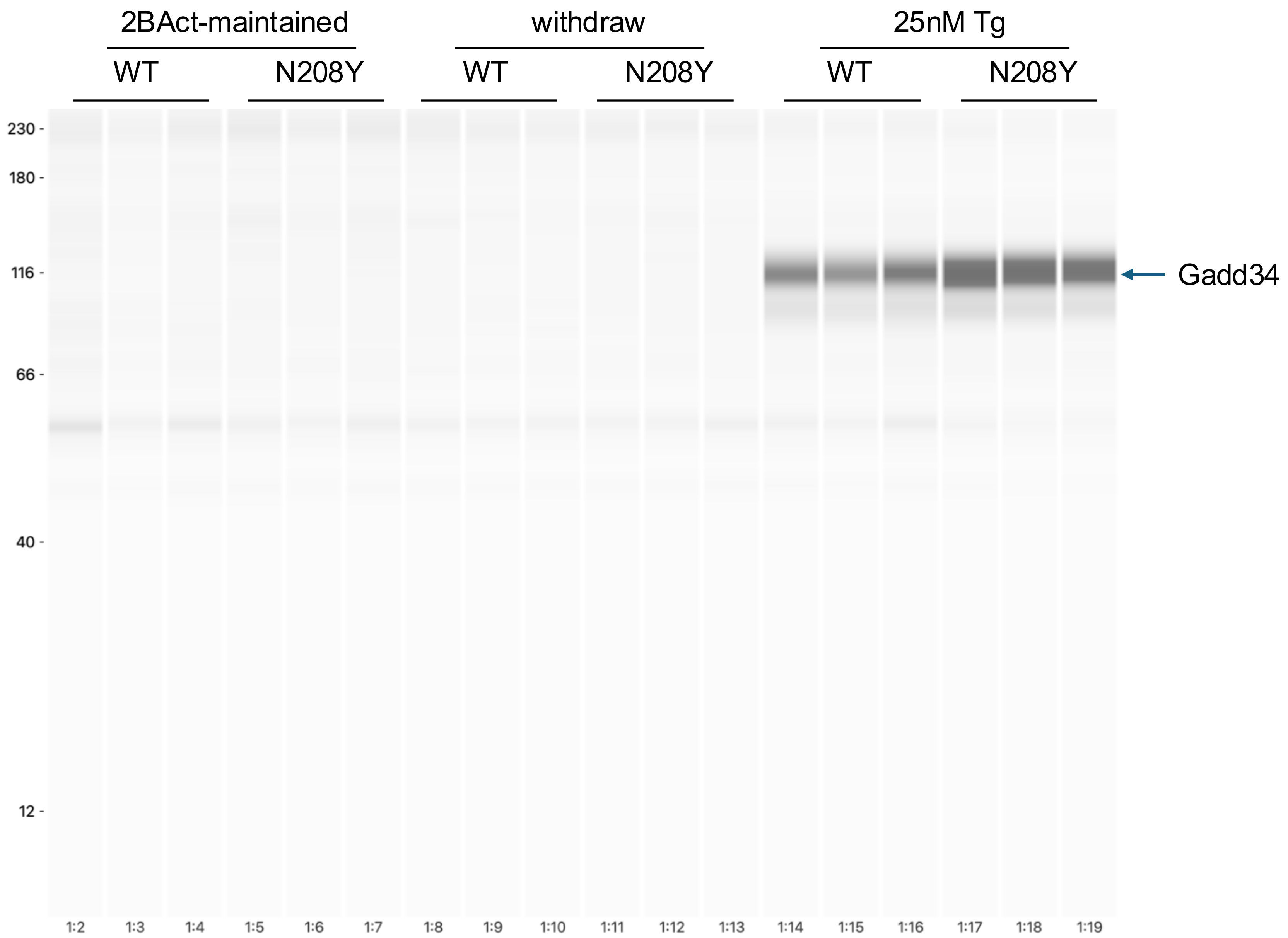

Full unedited blot for Supplement Figure10E

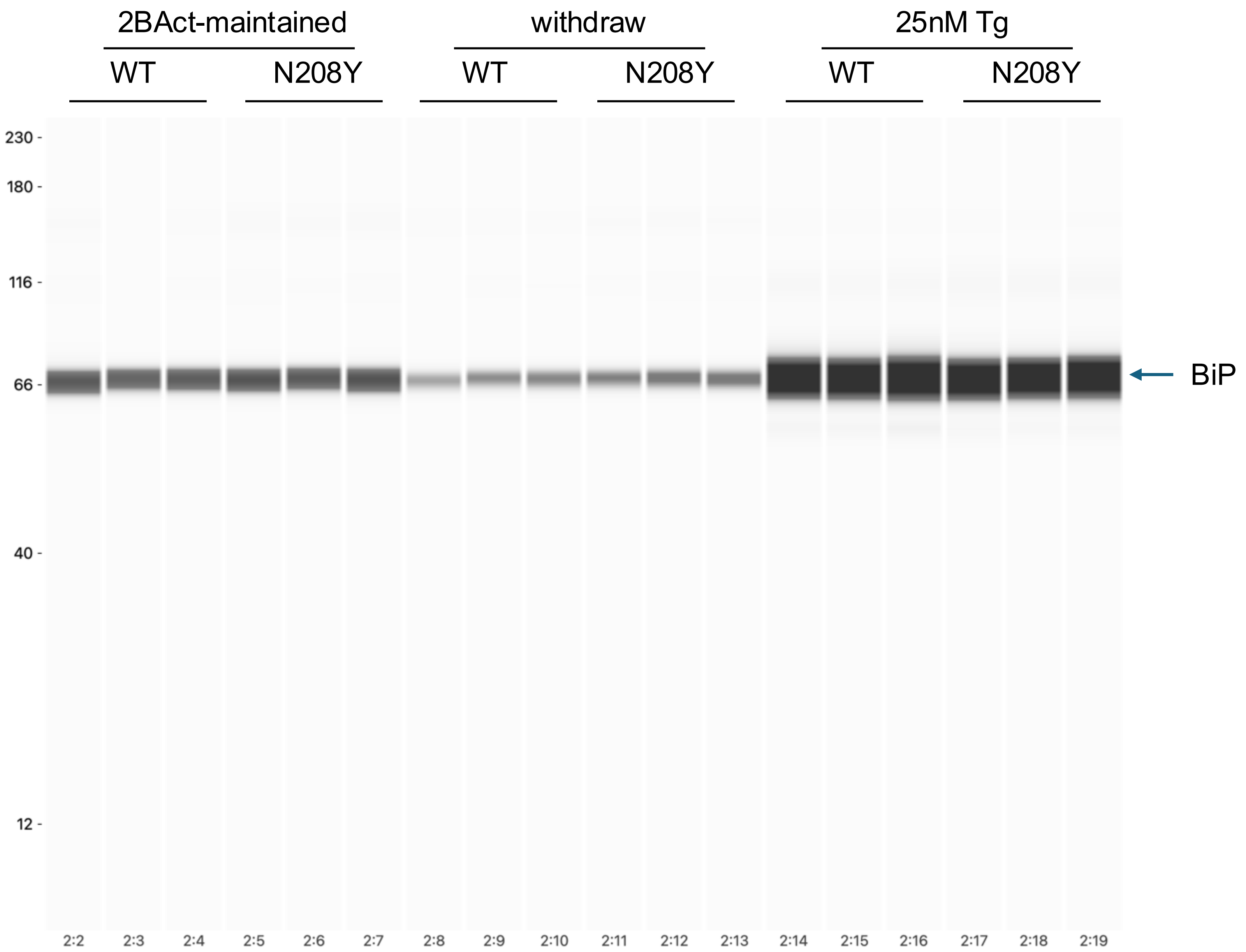

Full unedited blot for Supplement Figure10E

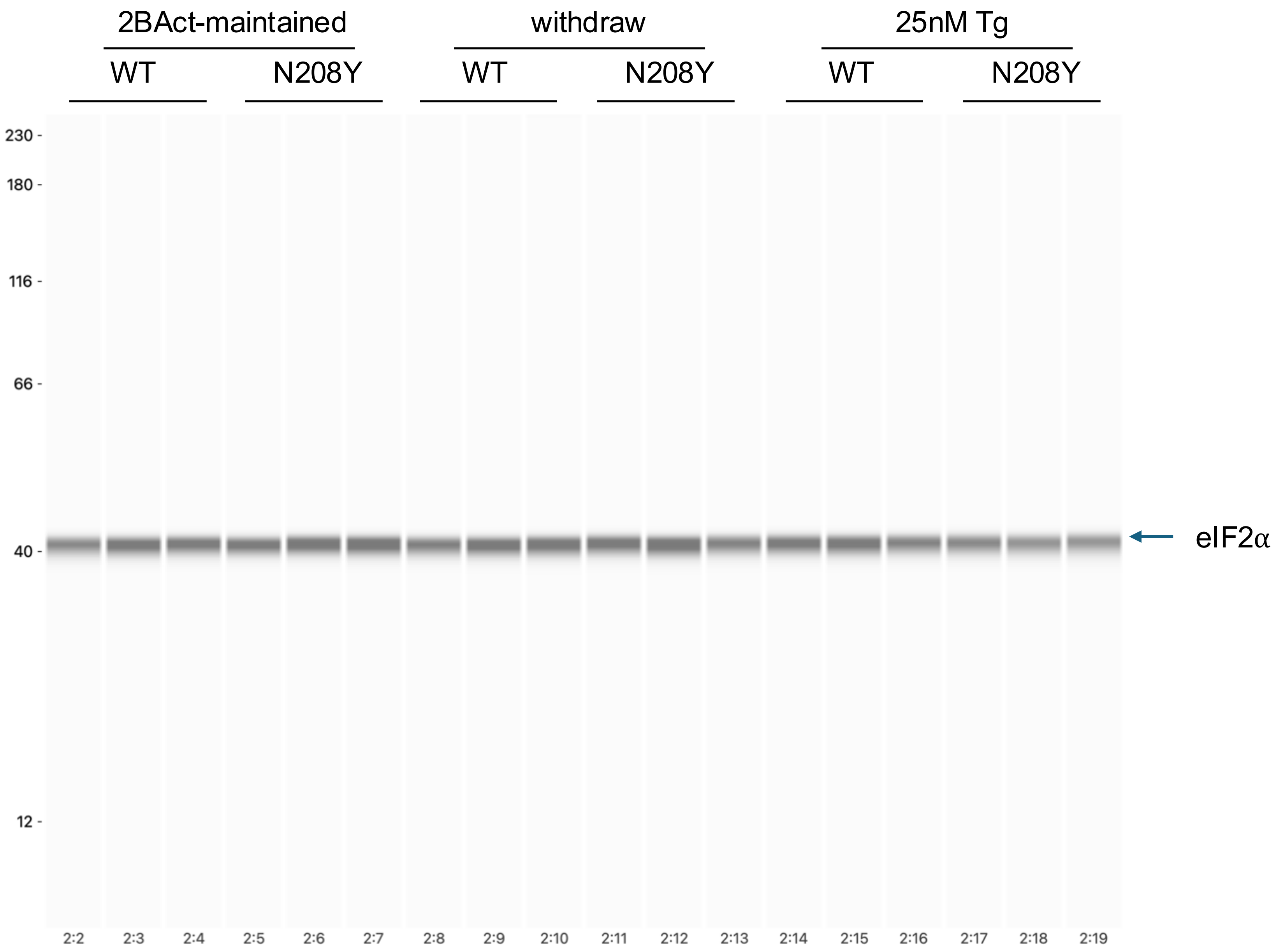

Supplement: Unedited blot and gel images [file jciinsight-10-188459-s209.pdf]
